# Total Synthesis of the Highly *N*-Methylated Peptides Carmabin A and Dragomabin

**DOI:** 10.3390/md16090338

**Published:** 2018-09-17

**Authors:** Baijun Ye, Peng Jiang, Tingrong Zhang, Yuanjun Sun, Xin Hao, Yingjun Cui, Liang Wang, Yue Chen

**Affiliations:** 1 The State Key Laboratory of Medicinal Chemical Biology, College of Pharmacy and Tianjin Key Laboratory of Molecular Drug Research, Nankai University, Tianjin 300350, China; yebaijunts@126.com (B.Y.); jiang1921372889@126.com (P.J.); nku2120181185@126.com (T.Z.); sunyuanjun7818@163.com (Y.S.); haoxinbit@126.com (X.H.); cyj10080@126.com (Y.C.); 2 Collaborative Innovation Center of Chemical Science and Engineering, Tianjin 300350, China

**Keywords:** total synthesis, stereochemical determination/revision, carmabin A, dragomabin

## Abstract

The first total synthesis of carmabin A and dragomabin was achieved at 52.3 mg and 43.8 mg scale, respectively. The synthesis led to determination of the configuration of carmabin A and reassignment of the configuration of dragomabin at the stereogenic centre on the alkyne-bearing fragment.

## 1. Introduction

Secondary metabolites produced by marine microorganisms are a rich source of valuable pharmaceuticals ranging from antimalarial agents to anticancer agents [1,2,3,4]. Marine cyanobacteria are widely distributed throughout the world [5] and produce a large number of structurally complex secondary metabolites containing alkynyl groups [6]. Due to their intriguing biological activities, these compounds have attracted the attention of many synthetic chemists [7,8,9,10,11,12,13], including us [14]. Carmabin A and dragomabin, two acetylene-containing lipopeptides, were discovered in 1998 [15,16] and 2007 [16] as secondary metabolites of cyanobacterium *Lyngbyamajuscula* in Panama by Gerwick et al. Structurally characterized with multiple *N*-methylated amino acids and a lipid chain (Mdya for carmabin A, Moya for dragomabin), carmabin A showed antimalarial activity (4.3 µM) towards the W2 chloroquine-resistant malaria strain and inhibitory activity towards mammalian Vero cells. Dragomabin, which has lost a propyl unit compared to carmabin A, showed antimalarial activity (6.0 µM) towards the W2 chloroquine-resistant malaria strain but very weak cytotoxicity to Vero cells. Thus, dragomabin possesses significant differential toxicity between parasite and mammalian cells. However, due to the scarceness of carmabin A and dragomabin, the medicinal chemistry as well as the mechanism of action have not been explored.

Without sufficient material, Gerwick et al. could not degrade carmabin A to obtain free Mdya to determine the configuration of the stereogenic centre by comparison with synthetic isomers [16]. They proposed a configuration of 35*S*, 37*R* or 35*R*, 37*S* for the Mdya fragment of carmabin A according to the strong correlations between H-35 and CH_3_-45 and between H-35 and CH_3_-33 [16]. The absolute stereochemistry of dragomabin was not clearly elucidated. Gerwick et al. proposed that dragomabin possesses a configuration of 35*S* for the Moya fragment according to the comparison of spectroscopic data between dragomabin and 35*S-*dragonamide A and 35*R-*dragonamide A [16].

In addition, the highly *N*-methylated amino acids of carmabin A and dragomabin led to two or more discernible conformers, which complicated the determination of absolute configuration (e.g., carmabin A: five conformers with a ratio of 23:6:5:1:1 in DMSO-*d*_6_^1^H NMR spectrum) [15]. Synthetic access to useful quantities of carmabin A and dragomabin has been hampered by their unknown stereochemical configurations and conformers caused by the *N*-methyl groups, which further undermines investigation of those lipopeptides as potential lead compounds as well as the study of their mechanism of action. Further medicinal research enabled by chemical synthesis of carmabin A and dragomabin may provide new leads for drug discovery. Herein, we report the first total synthesis and absolute stereochemical determination of carmabin A at C35, C37. In addition, we report the first total synthesis of dragomabin with a revision of the stereochemical configuration at C35.

## 2. Results

**Scheme 1:**
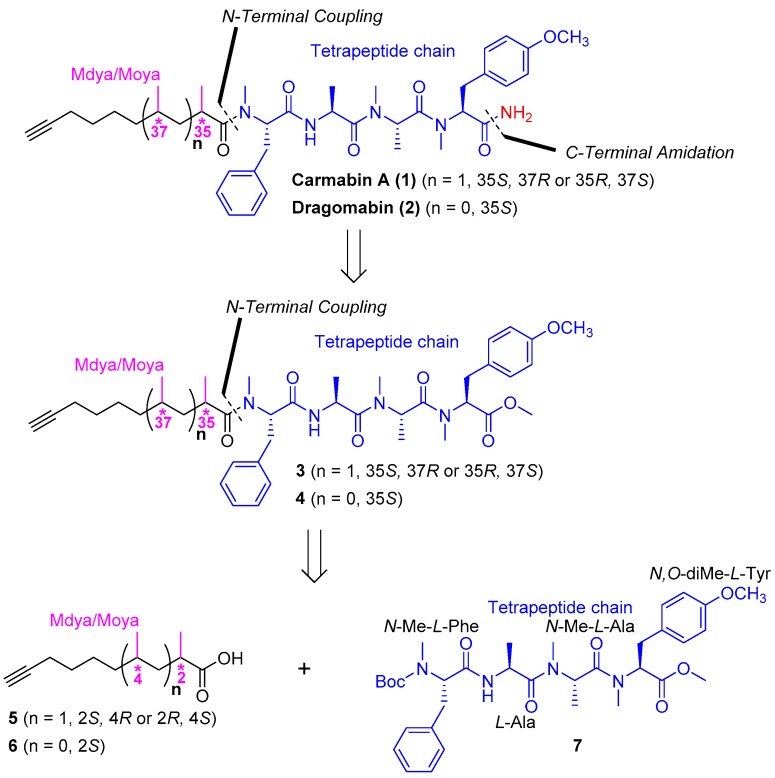
Retrosynthetic analysis of carmabin A (**1**) and dragomabin (**2**).

Scheme 1 illustrates our retrosynthetic analysis. The *C-*terminal amide of carmabin A and dragomabin could be prepared via amidation of the *C*-terminal methyl esters of compounds **3** and **4**. Compounds **3** and **4** were further disconnected into two parts: the Mdya/Moya fragment and protected tetrapeptide **7**. Tetrapeptide **7** could be prepared by repeated condensation reactions of amino acids. The methyl group of Mdya/Moya **5**/**6** could be stereoselectively installed following the methodology developed by Evans [17,18,19].

### 2.1. Synthesis of Mdya ***5*** of Carmabin A

**Scheme 2:**
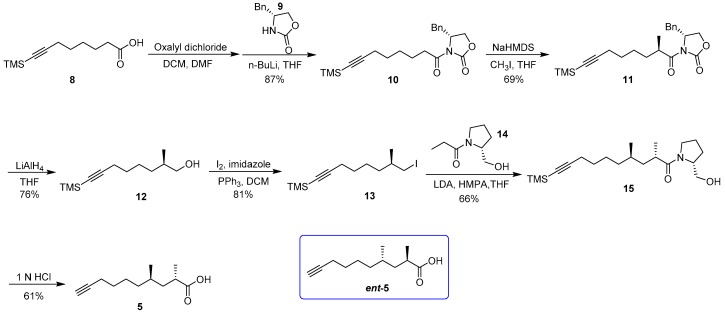
Synthesis of Mdya **5**.

The forward synthesis started with the synthesis of acid **5** (Scheme 2). Carboxylic acid **8** was prepared according to the literature. By employing the route we previously developed for the synthesis of ***ent*-5** [14], **8** was converted to corresponding alcohol **12** in a four-step sequence involving acyl chlorination, amidation with benzyl-2-oxazolidinone, diastereoselective α-methylation, and reduction in 46% overall yield (four steps). Alcohol **12** was converted to iodide **13** under Appel reaction conditions [20]. Iodide **13** was then subjected to asymmetric alkylation with the enolate generated from **14** to afford **15** [17] in 66% yield (d.r. > 20:1 according to ^1^H NMR), which constructed the 2*S*,4*R* stereochemistry on the Mdya fragment. Treatment of resulting **15** with 1 N HCl led to the hydrolysis of both the TMS group and the amide bond, providing **5** in 61% yield (2.1 g scale, one batch).

### 2.2. Synthesis of Tetrapeptide ***7***

**Scheme 3:**
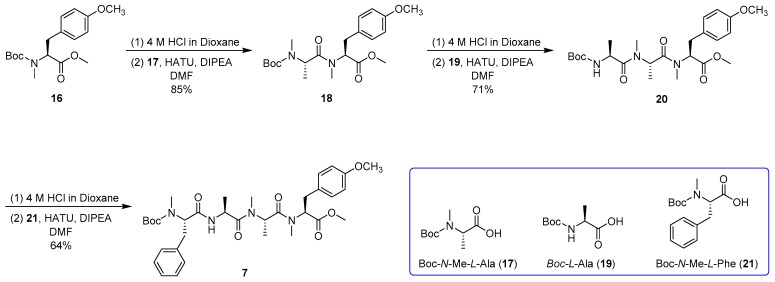
Synthesis of tetrapeptide **7**.

Scheme 3 depicts the construction of tetrapeptide **7**. Boc deprotection of **16** using TFA followed by condensation with **17** under the conventional coupling conditions (HATU/DIPEA) discovered by Louis A. Carpino [21] produced **18** in 85% yield. By repeating the condensation under the same conditions, Boc-*l*-Ala **19** and Boc-*N*-Me-*l*-Phe **21** were sequentially introduced, providing protected tetrapeptide **7**. The relatively low yield of the condensation can probably be attributed to the steric hindrance imposed by the additional methyl group. Problematic undesired diketopiperazine (DKP) formation [22,23] was not observed during the condensation reaction.

### 2.3. Total Synthesis of Carmabin A and Discussion

**Scheme 4:**
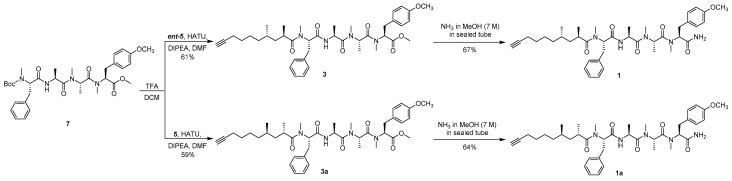
Total synthesis of carmabin A (**1**).

With **7**, **5**, ***ent*-5** in hand, we next aimed at completing the total synthesis of carmabin A and confirming its absolute stereochemistry (Scheme 4). The final steps involved coupling reactions between Boc-deprotected **7** with **5** and with ***ent*-5** followed by treatment with ammonia to produce **1** and **1a**. After deprotection of **7** under 4 M HCl, the resulting intermediate was subjected to coupling reaction directly, while only a trace amount of condensation product was observed. This could be explained by that **7** or Boc-deprotected **7** are unstable under 4 M HCl. By switching 4 M HCl for 10% TFA, the coupling reaction proceeded smoothly to provide **3** and **3a** in 61% and 59% yields, respectively. The transformation from **3** to **1** was first conducted in anhydrous NH_3_ solution (7.0 M in MeOH) at room temperature. With most of starting material left in the reaction system, only 10% yield of **1** was observed. Elevating the temperature to 40 °C improved the yield to 40%. When the reaction was transferred to a sealed tube in 60 °C, **1** and **1a** were obtained in 67% and 64% yield, respectively. After careful comparison, the spectroscopic data of synthetic **1** were consistent within the margin of error (0.02 ppm for ^1^H NMR and 0.2 ppm for ^13^C NMR) for the data originally reported for natural **1**. The NMR data of synthetic **1a** did not match the data of natural **1**. Consequently, the absolute configuration of carmabin A was established as shown in **1**.

### 2.4. Synthesis of Moya ***6*** of Dragomabin

**Scheme 5:**
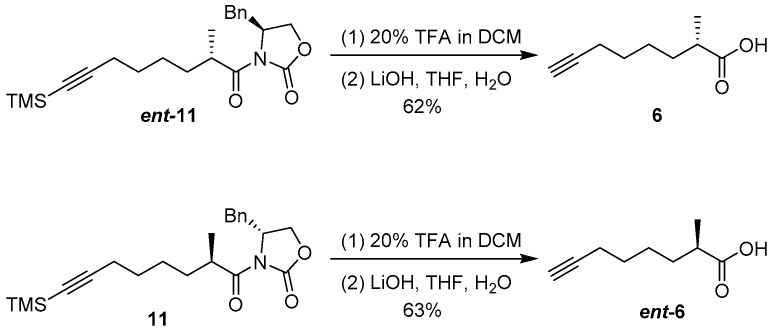
Synthesis of Moya **6** and ***ent*****-6**.

Next, we turned our attention to dragomabin. The Moya **6** fragment of dragomabin was obtained from ***ent*-11** via deprotection of TMS with TFA followed by removal of the chiral auxiliary (Scheme 5).

### 2.5. Total Synthesis of Dragomabin and Discussion

**Scheme 6:**
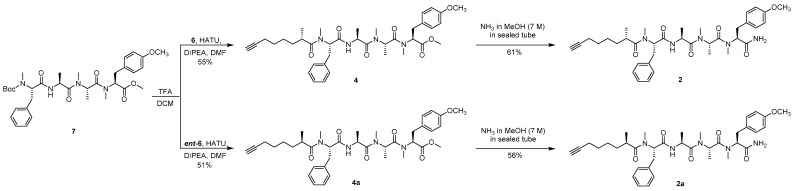
Total synthesis of dragomabin (**2**).

After deprotection of **7** under 4 M HCl, the resulting intermediate was subjected to a coupling reaction, which proceeded smoothly to provide **4** in 55% yield. Amidation of **4** with anhydrous NH_3_ solution afforded compound **2** in 61% yield (Scheme 6). Compound **2** was prepared with the same stereochemistry as that reported in the isolation paper. However, neither the ^1^HNMR data nor the optical rotation data are consistent with the reported values. The main concern regarding the true structure was the configuration at C35. By employing the same procedure described above, ***ent-*6** was obtained from **11** and then subjected to condensation with tetrapeptide **7** to give **2a**. It was indeed found that the data for **2a** were consistent with those of natural dragomabin. Thus, the correct structure for dragomabin was revised as shown in **2a**. As dragomabin and dragonamide differ in the stereochemistry on the Moya fragment, it appears that the stereochemistry on the alkyne fragment in these lipopeptides is variable and correlation with other natural products is not reliable.

## 3. Materials and Methods 

### 3.1. General Information

Reagents were purchased from commercial suppliers and used without purification unless otherwise stated: hexamethylphosphoric triamide (HMPA), ethyl acetate (EtOAc), *N*,*N*-dimethylformamide (DMF), lithium diisopropylamide (LDA), dichloromethane (DCM), hydrochloric acid (HCl), trifluoroacetic acid (TFA), 1-(Bis(dimethylamino)methylene)-1*H*-1,2,3-triazolo(4,5-b)pyridinium 3-oxid hexafluorophosphate (HATU), and *N*,*N*-Diisopropy-lethylamine (DIPEA).

All reactions were carried out under an argon atmosphere with dry solvents under anhydrous conditions, unless otherwise noted. Tetrahydrofuran (THF) was distilled immediately before use from sodium-benzophenone ketyl. Diisopropylamine was distilled from calcium hydride. Solvents for chromatography were used as supplied by Tianjin Reagents Chemical (Tianjin, China). Reactions were monitored by thin-layer chromatography (TLC) carried out on silica gel plates, using UV light as the visualizing agent and aqueous phosphomolybdic acid or basic aqueous potassium permanganate as the developing agent. A 200–300 mesh silica gel was used for column chromatography. 

Optical rotations were recorded on an Insmark IP 120 digital polarimeter (Insmark, Shanghai, China). IR spectra were recorded on a Bruker Tensor 27 instrument (Ettlingen, Germany). Only the strongest and/or most structurally important absorptions of IR spectra were reported in wavenumbers (cm^−1^). ^1^H NMR, ^13^C NMR, and 2D NMR were recorded on Bruker AV 400 and calibrated by using internal references and solvent signals CDCl_3_ (δ_H_ = 7.26 ppm, δ_C_ = 77.16 ppm), unless otherwise noted. ^1^H NMR data are reported as follows: chemical shift, multiplicity (s = singlet, d = doublet, t = triplet, q = quartet, p = quintet, br = broad, m = multiplet), coupling constants and integration. High-resolution mass spectra (HRMS) were detected on an IonSpec FT-ICR mass spectrometer by Varian 7.0T FTMS (Kuala Lumpur, Malaysia). 

### 3.2. Chemistry

*(2S,4R)-2,4-Dimethyldec-9-ynoic acid* (**5**) was obtained following the procedure for the preparation of compound (2*R*,4*S*)-2,4-dimethyldec-9-ynoic acid(***ent*-5**) [14].

*(R)-4-Benzyl-3-(8-(trimethylsilyl)oct-7-ynoyl)oxazolidin-2-one* (**10**) Yield: 87%; ${\left[\alpha \right]}_{D}^{21}$ −39.5 (*c* 1.0, CHCl_3_); IR (KBr) ν_max_ 2959, 2932, 2172, 1796, 1701, 1244, 1056, 759, 644 cm^−1^; ^1^H NMR (400 MHz, CDCl_3_) δ 7.35–7.20 (m, 5H), 4.70–4.64 (m, 1H), 4.22–4.16 (m, 2H), 3.30 (dd, *J* = 13.3, 3.1 Hz, 1H), 3.02–2.85 (m, 2H), 2.76 (dd, *J* = 13.3, 9.7 Hz, 1H), 2.25 (t, *J* = 7.0 Hz, 2H), 1.76–1.67 (m, 2H), 1.60–1.43 (m, 4H), 0.14 (s, 9H); ^13^C NMR (100 MHz, CDCl_3_) δ 173.3, 153.6, 135.4, 129.5, 129.1, 127.5, 107.4, 84.7, 66.3, 55.3, 38.1, 35.5, 28.5, 28.4, 23.9, 19.8, 0.3; HRESIMS *m*/*z* 394.1815 [M + Na]^+^ (calcd. for C_21_H_29_NNaO_3_Si^+^, 394.1809).

*(R)-4-Benzyl-3-((R)-2-methyl-8-(trimethylsilyl)oct-7-ynoyl)oxazolidin-2-one* (**11**) Yield: 69%; ${\left[\alpha \right]}_{D}^{20}$ −52.3 (*c* 1.0, CHCl_3_); IR (KBr) ν_max_ 2939, 2862, 2174, 1783, 1699, 1211, 761, 640 cm^−1^; ^1^H NMR (400 MHz, CDCl_3_) δ 7.35–7.20 (m, 5H), 4.67 (qd, *J* = 6.6, 2.8 Hz, 1H), 4.23–4.14 (m, 2H), 3.73–3.67 (m, 1H), 3.27 (dd, *J* = 13.3, 2.9 Hz, 1H), 2.77 (dd, *J* = 13.3, 9.6 Hz, 1H), 2.22 (t, *J* = 7.0Hz, 2H), 1.77–1.75 (m, 1H), 1.53–1.35 (m, 5H), 1.23 (d, *J* = 6.8 Hz, 3H), 0.14 (s, 9H); ^13^C NMR (100 MHz, CDCl_3_) δ 177.3, 153.2, 135.5, 129.6, 129.1, 127.5, 107.4, 84.7, 66.2, 55.5, 38.1, 37.8, 32.9, 28.7, 26.5, 19.9, 17.4, 0.3; HRESIMS *m*/*z* 408.1969 [M + Na]^+^ (calcd. for C_22_H_31_NNaO_3_Si^+^, 408.1965).

*(R)-2-Methyl-8-(trimethylsilyl)oct-7-yn-1-ol* (**12**) Yield: 76%; ${\left[\alpha \right]}_{D}^{20}$ +6.3 (*c* 1.0, CHCl_3_); IR (KBr) ν_max_ 3372, 2958, 2937, 2175, 1250, 1044, 760, 640 cm^−1^; ^1^H NMR (400 MHz, CDCl_3_) δ 3.54–3.40 (m, 2H), 2.23 (t, *J* = 6.9 Hz, 2H), 1.67–1.60 (m, 1H), 1.55–1.35 (m, 5H), 1.28 (t, *J* = 5.5 Hz, 1H), 1.19–1.06 (m, 1H), 0.92 (d, *J* = 6.7 Hz, 3H), 0.14 (s, 9H); ^13^C NMR (100 MHz, CDCl_3_) δ 107.7, 84.6, 68.4, 35.8, 32.6, 28.9, 26.2, 19.9, 16.6, 0.3; compound **12** was not observed by HRESIMS analysis due to complete fragmentation.

*(R)-(8-Iodo-7-methyloct-1-yn-1-yl)trimethylsilane* (**13**) Yield: 81%; ${\left[\alpha \right]}_{D}^{21}$ −2.8 (*c* 1.0, CHCl_3_); IR (KBr) ν_max_ 2959, 2935, 2174, 1249, 1195, 760, 640 cm^−1^; ^1^H NMR (400 MHz, CDCl_3_) δ 3.25–3.13 (m, 2H), 2.23 (t, *J* = 6.9 Hz, 2H), 1.53–1.32 (m, 6H), 1.28–1.18 (m, 1H), 0.98 (d, *J* = 6.4 Hz, 3H), 0.15 (s, 9H); ^13^C NMR (100 MHz, CDCl_3_) δ 107.5, 84.8, 36.0, 34.7, 28.6, 26.1, 20.7, 19.9, 17.8, 0.3; compound 13 was not observed by HRESIMS analysis due to complete fragmentation.

*(2S,4R)-1-((R)-2-(Hydroxymethyl)pyrrolidin-1-yl)-2,4-dimethyl-10-(trimethylsilyl)dec-9-yn-1-one* (**15**) Yield: 66%; ${\left[\alpha \right]}_{D}^{21}$ +30.2 (*c* 1.0, CHCl_3_); IR (KBr) ν_max_ 3431, 2959, 2935, 2174, 1620, 1463, 1249, 1052, 759, 640 cm^−1^; ^1^H NMR (400 MHz, CDCl_3_) δ 5.21 (dd, *J* = 7.7, 2.1 Hz, 1H), 4.23 (dd, *J* = 13.7, 5.8 Hz, 1H), 3.69–3.46 (m, 4H), 2.62 (dd, *J* = 13.4, 6.7 Hz, 1H), 2.20 (t, *J* = 6.9 Hz, 2H), 2.07–1.84 (m, 3H), 1.59–1.23 (m, 10H), 1.12 (d, *J* = 6.7 Hz, 3H), 0.86 (d, *J* = 5.8 Hz, 3H), 0.14 (s, 9H); ^13^C NMR (100 MHz, CDCl_3_) δ 178.7, 107.6, 84.5, 68.0, 61.3, 48.0, 40.7, 36.9, 35.9, 30.5, 28.9, 28.5, 26.1, 24.7, 19.9, 19.5, 17.4, 0.3; HRESIMS *m*/*z* 374.2490 [M + Na]^+^ (calcd. for C_20_H_37_NNaO_2_Si^+^, 374.2486).

*(2S,4R)-2,4-Dimethyldec-9-ynoic acid* (**5**) Yield: 61%; ${\left[\alpha \right]}_{D}^{22}$ +5.6 (*c* 1.0, CHCl_3_); IR (KBr) ν_max_ 3309, 2937, 2862, 2118, 1706, 1292, 633 cm^−1^; ^1^H NMR (400 MHz, CDCl_3_) δ 2.59–2.50 (m, 1H), 2.19 (td, *J* = 6.9, 2.4 Hz, 2H), 1.94 (t, *J* = 2.4 Hz, 1H), 1.59–1.31 (m, 9H), 1.16 (d, *J* = 6.9 Hz, 3H), 0.87 (d, *J* = 6.2 Hz, 3H); ^13^C NMR (100 MHz, CDCl_3_) δ 182.7, 84.8, 68.3, 40.9, 37.1, 36.5, 30.5, 28.8, 26.1, 19.4, 18.5, 17.0; HRESIMS *m*/*z* 195.1388 [M − H ]^−^ (calcd. for C_12_H_19_O^−^, 195.1391); the optical rotation of ***ent-5*** (${\left[\alpha \right]}_{D}^{20}$ −5.8 (*c* 1.0, CHCl_3_)).

*Boc*-*N*-*Me*-*l*-*Ala-N,O-diMe*-*l*-*Tyr* (**18**) Compound **16** (30.0 g, 92.8 mmol) was dissolved in 4.0 M HCl in dioxane (150 mL). After the mixture had been stirred for 3 h at room temperature, the whole mixture was concentrated to afford an HCl salt as a white solid and used directly for the next step. To the solution of the HCl salt, compound **17** (37.7 g, 185 mmol), and HATU (70.3 g, 185 mmol) in DMF (150 mL) was added DIPEA (48.0 g, 371 mmol) at 0 °C under argon, and then the mixture was warmed to room temperature and stirred overnight. After being diluted with EtOAc (800 mL), the whole mixture was washed with 1% HCl (3 × 150 mL), 5% aqueous NaHCO_3_ (3 × 150 mL), and brine (3 × 150 mL), and the organic layer was dried over Na_2_SO_4_ and filtered. The filtrate was concentrated and purified by silica gel column chromatography (10/1 to 3/1 petroleum ether/EtOAc) to afford compound **18** (32.2 g, 85%) as a colorless oil: ${\left[\alpha \right]}_{D}^{24}$ −121.2 (*c* 1.0, CHCl_3_); IR (KBr) ν_max_ 2978, 1745, 1657, 1515, 1249, 833, 561 cm^−1^; ^1^H NMR (400 MHz, CDCl_3_) mixture of rotamers δ 7.14, 7.09 (d, *J* = 8.3 Hz, 2H), 7.09 (d, *J* = 8.3 Hz, 1H), 6.84, 6.80 (d, *J* = 8.5 Hz, 2H), 5.37, 5.12, 5.05 (dd, *J* = 11.4, 4.8 Hz, 1H), 5.02, 4.80, 4.70, 4.63, 4.33 (q, *J* = 6.6 Hz, 1H), 3.76 (s, 3H), 3.73 (s, 3H), 3.35–3.28 (m, 1H), 3.03–2.93 (m, 1H), 2.85, 2.84, 2.80 (s, 3H), 2.58, 2.55, 2.23, 2.19 (s, 3H), 1.44, 1.43, 1.42 (s, 9H), 1.19, 1.09, 0.97, 0.90 (d, *J* = 6.7 Hz, 3H); ^13^C NMR (100 MHz, CDCl_3_) mixture of rotamers δ 171.9, 171.7, 171.3, 171.1, 158.7, 158.6, 155.3, 155.2, 130.1, 130.0, 129.8, 129.2, 128.5, 114.3, 114.1, 80.1, 80.0, 61.0, 60.6, 59.5, 58.6, 55.4, 52.5, 52.4, 51.7, 50.8, 49.5, 34.6, 33.8, 32.9, 31.9, 30.0, 29.8, 28.9, 28.6, 28.4, 28.1, 14.4; HRESIMS *m*/*z* 431.2155 [M + Na]^+^ (calcd. for C_21_H_32_N_2_NaO_6_^+^, 431.2153).

*Boc*-*l*-*Ala-N-Me-l-Ala-N,O-diMe*-*l*-*Tyr* (**20**) Compound **18** (31.0 g, 75.9 mmol) was dissolved in 4.0 M HCl in dioxane (150 mL). After the mixture had been stirred for 3 h at room temperature, the whole mixture was concentrated to afford an HCl salt as a white solid and used directly for the next step. To the solution of the HCl salt, compound **19** (37.7 g, 152 mmol), and HATU (57.7 g, 152 mmol) in DMF (150 mL) was added DIPEA (39.2 g, 303 mmol) at 0 °C under argon, and then the mixture was warmed to room temperature and stirred overnight. After being diluted with EtOAc (800 mL), the whole mixture was washed with 1% HCl (3 × 150 mL), 5% aqueous NaHCO_3_ (3 × 150 mL), and brine (3 × 150 mL), and the organic layer was dried over Na_2_SO_4_ and filtered. The filtrate was concentrated and purified by silica gel column chromatography (5/1 to 1/1 petroleum ether/EtOAc) to afford compound **20** (25.8 g, 71%) as a colorless oil: ${\left[\alpha \right]}_{D}^{24}$ −93.4 (*c* 1.0, CHCl_3_); IR (KBr) ν_max_ 3445, 2981, 1743, 1637, 1516, 1249, 1172, 614, 556 cm^–1^; ^1^H NMR (400 MHz, CDCl_3_) mixture of rotamers δ 7.09 (d, *J* = 8.5 Hz, 1H), 6.79 (d, *J* = 8.5 Hz, 1H), 5.47–5.33 (m, 2H), 4.85–4.37 (m, 1H), 3.77, 3.75 (s, 3H), 3.74, 3.71 (s, 3H), 3.34, 3.26 (dd, *J* = 14.8, 4.9 Hz, 1H), 2.97–2.88 (m, 1H), 2.92, 2.85, 2.71 (s, 3H), 2.83, 2.77, 2.28 (s, 3H), 1.41 (s, 9H), 1.27, 1.18, 1.11, 0.81 (d, *J* = 6.7 Hz, 1H); ^13^C NMR (100 MHz, CDCl_3_) mixture of rotamers δ 172.3, 171.3, 171.1, 170.7, 158.8, 155.1, 130.3, 129.9, 129.7, 128.8, 128.4, 114.3, 114.2, 114.1, 79.8, 58.2, 58.1, 55.4, 55.36, 52.6, 52.5, 49.7, 44.0, 46.6, 34.5, 33.8, 31.9, 31.8, 30.3, 30.1, 29.7, 29.2, 28.5, 19.0, 18.8, 14.1; HRESIMS *m*/*z* 502.2528 [M + Na]^+^ (calcd. for C_24_H_37_N_3_NaO_7_^+^, 502.2524).

*Boc-N-Me*-*l*-*Phe*-*l*-*Ala-N-Me*-*l*-*Ala-N,O-diMe*-*l*-*Tyr* (**7**) Compound **20** (25.0 g, 52.1 mmol) was dissolved in 4.0 M HCl in dioxane (125 mL). After the mixture had been stirred for 3 h at room temperature, the whole mixture was concentrated to afford an HCl salt as a white solid and used directly for the next step. To the solution of the HCl salt, compound **21** (29.1 g, 104 mmol), and HATU (39.6 g, 104 mmol) in DMF (125 mL) was added DIPEA (26.9 g, 208 mmol) at 0 °C under argon, and then the mixture was warmed to room temperature and stirred overnight. After being diluted with EtOAc (600 mL), the whole mixture was washed with 1% HCl (3 × 120 mL), 5% aqueous NaHCO_3_ (3 × 120 mL), and brine (3 × 120 mL), and the organic layer was dried over Na_2_SO_4_ and filtered. The filtrate was concentrated and purified by silica gel column chromatography (2/1 to 1/2 petroleum ether/EtOAc) to afford compound 7 (21.3 g, 64%) as a colorless oil: ${\left[\alpha \right]}_{D}^{24}$ −100.6 (*c* 1.0, CHCl_3_); IR (KBr) ν_max_ 3402, 2924, 1744, 1644, 1515, 1248, 1174, 615, 556 cm^−1^; ^1^H NMR (400 MHz, CDCl_3_) mixture of rotamers δ 7.27–7.15 (m, 5H), 7.14, 7.12 (d, *J* = 8.8 Hz, 2H), 6.96 (br, 1H), 6.85, 6.81 (d, *J* = 8.6 Hz, 2H), 5.40, 4.82 (m, 1H), 5.39–5.31 (m, 1H), 4.81–4.64 (m, 2H), 3.77 (s, 3H), 3.75, 3.72 (s, 3H), 3.42–3.26 (m, 2H), 3.00–2.89 (m, 2H), 2.86, 2.75, 2.72 (s, 3H), 2.72, 2.32 (s, 3H), 1.46, 1.37, 1.32 (s, 9H), 1.25, 1.14 (d, *J* = 6.8 Hz, 3H), 1.19, 0.81 (d, *J* = 6.8 Hz, 3H); ^13^C NMR (100 MHz, CDCl_3_) mixture of rotamers δ 171.7, 171.7, 171.6, 171.3, 171.2, 171.1, 158.9, 158.8, 130.3, 129.9, 129.2, 128.9, 128.6, 126.6, 114.4, 114.2, 58.4, 55.5, 52.5, 49.7, 48.1, 45.7, 34.3, 33.8, 32.0, 29.7, 29.2, 28.3, 18.4, 14.2, 14.1; HRESIMS *m*/*z* 663.3368 [M + Na]^+^ (calcd. for C_34_H_48_N_4_NaO_8_^+^, 663.3364).

*Methyl (2S,5S,8S,11S,14R,16S)-11-benzyl-2-(4-methoxybenzyl)-3,5,6,8,12,14,16-heptamethyl-4,7,10,13-tetraoxo-3,6,9,12-tetraazadocos-21-ynoate* (**3**) To the solution of compound **7** (200 mg, 0.312 mmol) in DCM (1.8 mL) was added TFA (0.2 mL). After the mixture had been stirred for 6 h at room temperature, toluene (2 mL) was added and then the whole mixture was concentrated under reduced pressure. The residue was dissolved with water (3 mL) and extracted with petroleum ether (3 × 1 mL). The aqueous phase was adjusted to pH 8–9 with Na_2_CO_3_ and extracted with DCM (3 × 10 mL), and the combined organic layer was dried over Na_2_SO_4_ and filtered. The filtrate was concentrated to afford a free amine as a colorless oil and used directly for the next step. To the solution of the free amine, ***ent*-5** (122 mg, 0.622 mmol), and HATU (237 mg, 0.624 mmol) in DMF (1 mL) was added DIPEA (84.7 mg, 0.655 mmol) at 0 °C under argon, and then the mixture was warmed to room temperature and stirred overnight. After being diluted with EtOAc (10 mL), the whole mixture was washed with 1% HCl (3 × 2 mL), 5% aqueous NaHCO_3_ (3 × 2 mL), and brine (3 × 2 mL), and the organic layer was dried over Na_2_SO_4_ and filtered. The filtrate was concentrated and purified by silica gel column chromatography (100/1 to 50/1 DCM/MeOH) to afford compound **3** (137 mg, 61%) as a colorless oil: ${\left[\alpha \right]}_{D}^{24}$ −150.6 (*c* 0.10, CHCl_3_); IR (KBr) ν_max_ 3446, 2934, 2115, 1743, 1636, 1515, 1248, 1179, 620, 560 cm^−1^; ^1^H NMR (400 MHz, CDCl_3_) mixture of rotamers δ 7.26–7.16 (m, 5H), 7.12, 7.09 (d, *J* = 8.5 Hz, 2H), 6.91 (d, *J* = 7.3 Hz, 1H), 6.85, 6.80 (d, *J* = 8.6 Hz, 2H), 5.48 (dd, *J* = 10.5, 6.2 Hz, 1H), 5.40, 4.86 (q, *J* = 6.6 Hz, 1H), 5.34, 4.81 (dd, *J* = 12.0, 4.8 Hz, 1H), 4.73, 4.62 (p, *J* = 6.8 Hz, 1H), 3.77 (s, 3H), 3.75, 3.71 (s, 3H), 3.38–3.23 (m, 2H), 3.04–2.84 (m, 2H), 2.90, 2.89 (s, 3H), 2.85, 2.71 (s, 3H), 2.80, 2.31 (s, 3H), 2.63–2.58 (m, 1H), 2.19–2.12 (m, 2H), 1.94 (t, *J* = 2.5 Hz, 1H), 1.47–1.39 (m, 2H), 1.31–1.22 (m, 2H), 1.21–0.96 (m, 5H), 1.18, 0.80 (d, *J* = 6.7 Hz, 3H), 1.17, 1.11 (d, *J* = 6.8 Hz, 3H), 1.05, 1.03 (d, *J* = 6.7 Hz, 3H), 0.72, 0.70 (d, *J* = 6.9 Hz, 3H); ^13^C NMR (100 MHz, CDCl_3_) mixture of rotamers δ 178.2, 178.2, 171.4, 171.4, 171.2, 171.1, 170.8, 170.7, 169.6, 169.6, 158.8, 158.7, 137.1, 137.0, 130.3, 129.8, 129.0, 128.9, 128.5, 128.3, 126.7, 114.3, 114.1, 84.7, 68.3, 61.0, 58.4, 57.0, 56.7, 55.4, 52.4, 49.6, 48.0, 45.6, 45.5, 40.9, 40.8, 36.5, 34.4, 33.9, 33.8, 33.7, 32.0, 31.2, 31.0, 30.2, 30.1, 29.6, 29.1, 28.8, 25.8, 19.6, 18.4, 18.3, 18.1, 17.0, 14.1, 14.0; HRESIMS *m*/*z* 741.4203 [M + Na]^+^ (calcd. for C_41_H_58_N_4_NaO_7_^+^, 741.4198).

*(2R,4S)-N-((S)-1-(((S)-1-(((S)-1-(((S)-1-Amino-3-(4-methoxyphenyl)-1-oxopropan-2-yl)(methyl)amino)-1-oxopropan-2-yl)(methyl)amino)-1-oxopropan-2-yl)amino)-1-oxo-3-phenylpropan-2-yl)-N,2,4-trimethyldec-9-ynamide* (carmabin A (**1**)) Compound **3** (80.0 mg, 0.111 mmol) was added to a sealed tube followed by anhydrous NH_3_ solution (7.0 M in MeOH, 1 mL). After the mixture had been stirred for 20 h at 60 °C, the whole mixture was concentrated and purified by silica gel column chromatography (50/1 to 20/1 DCM/MeOH) to afford a colorless oil; n-pentane (2.5 mL) was added, and then the mixture was stirred for 1 h at room temperature and filtered to afford compound **1** (52.3 mg, 67%) as a white powder: ${\left[\alpha \right]}_{D}^{24}$ −175.6 (c 0.44, CHCl_3_); IR (KBr) ν_max_ 3448, 2935, 2115, 1634, 1513, 1249, 1179, 670, 619 cm^−1^; HRESIMS *m*/*z* 726.4205 [M + Na]^+^ (calcd. for C_40_H_57_N_5_NaO_6_^+^, 726.4201). ^1^H and ^13^C NMR data in the Supporting Information.

*(S)-2-Methyloct-7-ynoic acid* (**6**) To the solution of compound ***ent*-11** (1.00 g, 2.59 mmol) in DCM (8 mL) was added TFA (2 mL). After the mixture had been stirred for 3 h at room temperature, toluene (10 mL) was added and then the whole mixture was concentrated under reduced pressure. The residue was dissolved with water (10 mL) and adjusted to pH 8–9 with Na_2_CO_3_ and extracted with DCM (3 × 20 mL), and the combined organic layer was dried over Na_2_SO_4_ and filtered. The filtrate was concentrated to afford a crude oil and used directly for the next step. To the crude oil in THF (10 mL) and water (2.5 mL) was added LiOH·H_2_O (435 mg, 10.4 mmol) and H_2_O_2_ (2.90 mL, 20.7 mmol, 30% in water) at 0 °C. After the mixture had been stirred for 3 h at room temperature. The whole mixture was adjusted to pH 2–3 with 1 N HCl and extracted with EtOAc (3 × 30 mL), and the combined organic layer was dried over Na_2_SO_4_ and filtered. The filtrate was concentrated and purified by silica gel column chromatography (petroleum ether/EtOAc = 50/1 − 30/1) to afford compound **6** (248 mg, 62%) as a colorless oil: ${\left[\alpha \right]}_{D}^{25}$ +13.2 (*c* 0.90, CHCl_3_); IR (KBr) ν_max_3304, 2942, 2865, 2118, 1706, 1236, 638 cm^−1^; ^1^H NMR (400 MHz, CDCl_3_) δ 2.52–2.42 (m, 1H), 2.20 (td, *J* = 6.9, 2.5 Hz, 2H), 1.94 (t, *J* = 2.5 Hz, 1H), 1.75–1.65 (m, 1H), 1.58–1.42 (m, 5H), 1.19 (d, *J* = 7.0 Hz, 3H); ^13^C NMR (100 MHz, CDCl_3_) δ 182.9, 84.4, 68.5, 39.3, 33.1, 28.4, 26.4, 18.4, 17.0; compound **6** was not observed by HRESIMS analysis due to complete fragmentation.

*(R)-2-methyloct-7-ynoic acid* (***ent*-6**) Compound ***ent*-6** was obtained from compound **11** (800 mg, 2.07 mmol) following the same procedure for the preparation of compound **6** (200 mg, 63%) colorless oil: ${\left[\alpha \right]}_{D}^{25}$ −13.0 (c 0.90, CHCl_3_); ^1^H NMR (400 MHz, CDCl_3_) δ 2.52–2.43 (m, 1H), 2.20 (td, *J* = 6.9, 2.5 Hz, 2H), 1.94 (t, *J* = 2.5 Hz, 1H), 1.74–1.65 (m, 1H), 1.58–1.42 (m, 5H), 1.19 (d, *J* = 7.0 Hz, 3H). The spectroscopic data are in agreement with those reported in the literature [9].

*Methyl(2S,5S,8S,11S,14S)-11-benzyl-2-(4-methoxybenzyl)-3,5,6,8,12,14-hexamethyl-4,7,10,13-tetraoxo-3,6,9,12-tetraazaicos-19-ynoate* (**4**) Compound **4** was obtained from compound 7 (200 mg, 0.312 mmol) and compound **6** (95.9 mg, 0.622 mmol) following the procedure for the preparation of compound **3**, (116 mg, 55%), colorless oil: ${\left[\alpha \right]}_{D}^{24}$ −59.8 (*c* 0.10, CHCl_3_); IR (KBr) ν_max_ 3444, 2935, 2115, 1744, 1642, 1515, 1248, 1179, 701, 618 cm^−1^; ^1^H NMR (400 MHz, CDCl_3_) mixture of rotamers δ 7.27–7.14 (m, 5H), 7.12, 7.09 (d, *J* = 8.9 Hz, 2H), 6.94 (d, *J* = 7.2 Hz, 1H), 6.86, 6.80 (d, *J* = 8.6 Hz, 2H), 5.47 (dd, *J* = 10.1, 6.2 Hz, 1H), 5.41, 4.84 (dd, *J* = 12.9, 6.2 Hz, 1H), 5.33, 4.76 (dd, *J* = 12.1, 7.3 Hz, 1H), 4.73, 4.62 (p, *J* = 6.9 Hz, 1H), 3.77 (s, 3H), 3.75, 3.72 (s, 3H), 3.39–3.24 (m, 2H), 3.02–2.91 (m, 2H), 2.90, 2.89 (s, 3H), 2.85, 2.78, 2.71 (s, 3H), 2.80, 2.34, 2.31 (s, 3H), 2.60–2.52 (m, 1H), 2.20–2.13 (m, 2H), 1.94–1.91 (m, 1H), 1.72–1.64 (m, 1H), 1.53–1.47 (m, 2H), 1.40–1.30 (m, 3H), 1.18, 1.10, 0.81 (d, *J* = 6.7 Hz, 3H), 1.18, 1.11, 1.05 (d, *J* = 6.8 Hz, 3H), 0.79 (d, *J* = 6.8 Hz, 3H); ^13^C NMR (100 MHz, CDCl_3_) mixture of rotamers δ 177.7, 177.6, 171.4, 171.3, 171.1, 170.9, 170.7, 170.5, 169.4, 169.4, 168.4, 158.7, 158.6, 137.4, 137.0, 136.9, 130.1, 129.7, 129.6, 129.1, 128.8, 128.7, 128.3, 128.1, 127.0, 126.5, 114.2, 114.0, 113.9, 84.4, 84.3, 68.5, 68.3, 68.2, 62.9, 60.9, 58.2, 56.9, 56.8, 55.3, 55.2, 52.4, 52.3, 49.5, 47.9, 45.8, 45.5, 45.3, 35.8, 35.4, 34.5, 34.3, 33.7, 33.6, 33.3, 33.2, 32.9, 31.9, 31.3, 31.2, 30.0, 29.6, 29.5, 29.0, 28.5, 28.4, 26.7, 26.6, 26.2, 18.2, 18.1, 18.0, 17.3, 14.0, 13.9; HRESIMS *m*/*z* 699.3732 [M + Na]^+^ (calcd. for C_38_H_52_N_4_NaO_7_^+^, 699.3728.

*(S)-N-((S)-1-(((S)-1-(((S)-1-(((S)-1-Amino-3-(4-methoxyphenyl)-1-oxopropan-2-yl)(methyl)amino)-1-oxopropan-2-yl)(methyl)amino)-1-oxopropan-2-yl)amino)-1-oxo-3-phenylpropan-2-yl)-N,2-dimethyloct-7-ynamide* (**2**) Compound **2** was obtained from compound **4** (80.0 mg, 0.118 mmol) following the procedure for the preparation of compound **1**, (47.7 mg, 61%), white powder: ${\left[\alpha \right]}_{D}^{25}$ −163.8 (*c* 0.50, CHCl_3_); IR (KBr) ν_max_ 3447, 2935, 2114, 1634, 1514, 1249, 1081, 700, 617; HRESIMS *m*/*z* 684.3736 [M + Na]^+^ (calcd. for C_37_H_51_N_5_NaO_6_^+^, 684.3732). ^1^H and ^13^C NMR data are in the Supplementary Materials.

*Methyl (2S,5S,8S,11S,14R)-11-benzyl-2-(4-methoxybenzyl)-3,5,6,8,12,14-hexamethyl-4,7,10,13-tetraoxo-3,6,9,12-tetraazaicos-19-ynoate* (**4a**) Compound **4a** was obtained from compound 7 (200 mg, 0.312 mmol) and compound ***ent*-6** (95.9 mg, 0.622 mmol) following the procedure for the preparation of compound **3**, (108 mg, 51%), colorless oil: ${\left[\alpha \right]}_{D}^{23}$ −105.0 (*c* 0.10, CHCl_3_); IR (KBr) ν_max_ 3447, 2931, 2115, 1636, 1385, 1271, 1179, 670, 615 cm^−1^; ^1^H NMR (400 MHz, CDCl_3_) mixture of rotamers δ 7.27–7.16 (m, 5H), 7.12, 7.09 (d, *J* = 8.7 Hz, 2H), 6.91 (d, *J* = 7.1 Hz, 1H), 6.85, 6.80 (d, *J* = 8.7 Hz, 2H), 5.45 (dd, *J* = 10.1, 6.2 Hz, 1H), 5.40, 4.83 (dd, *J* = 12.9, 6.4 Hz, 1H), 5.34, 4.76 (dd, *J* = 11.9, 4.8 Hz, 1H), 4.73, 4.62 (p, *J* = 6.9 Hz, 1H), 3.77 (s, 3H), 3.75, 3.72 (s, 3H), 3.38–3.26 (m, 2H), 3.05–2.92 (m, 2H), 2.91, 2.89 (s, 3H), 2.85, 2.77, 2.71 (s, 3H), 2.81, 2.34, 2.31 (s, 3H), 2.55–2.50 (m, 1H), 2.08–2.03 (m, 2H), 1.94–1.91 (m, 1H), 1.49–1.41 (m, 1H), 1.39–1.31 (m, 2H), 1.21–1.06 (m, 3H), 1.19, 1.10, 0.81 (d, *J* = 6.8 Hz, 3H), 1.17, 1.11, (d, *J* = 6.8 Hz, 3H), 1.07, 0.44 (d, *J* = 6.7 Hz, 3H); ^13^C NMR (100 MHz, CDCl_3_) mixture of rotamers δ 177.7, 177.6, 171.4, 171.2, 171.0, 170.8, 170.6, 169.5, 169.5, 158.8, 158.7, 137.5, 137.2, 137.1, 130.2, 129.8, 129.2, 128.9, 128.8, 128.5, 126.6, 114.3, 114.1, 84.5, 68.3, 63.2, 61.0, 58.4, 57.2, 56.8, 55.3, 52.4, 52.4, 49.6, 47.9, 45.5, 45.4, 36.0 34.4, 33.7, 33.7, 33.3, 32.0, 31.4, 31.1, 30.1, 29.6, 29.1, 28.5, 26.3, 18.3, 18.2, 18.1, 17.4, 17.4, 14.1, 14.0; HRESIMS *m*/*z* 699.3732 [M + Na]^+^ (calcd. for C_38_H_52_N_4_NaO_7_^+^, 699.3728).

*(R)-N-((S)-1-(((S)-1-(((S)-1-(((S)-1-Amino-3-(4-methoxyphenyl)-1-oxopropan-2-yl)(methyl)amino)-1-oxopropan-2-yl)(methyl)amino)-1-oxopropan-2-yl)amino)-1-oxo-3-phenylpropan-2-yl)-N,2-dimethyloct-7-ynamide* (dragomabin (**2a**)) Compound **2a** was obtained from compound **4a** (80.0 mg, 0.118 mmol) following the procedure for the preparation of compound **1**, (43.8 mg, 56%), white powder: ${\left[\alpha \right]}_{D}^{25}$ −118.3 (*c* 0.50, CHCl_3_); IR (KBr) ν_max_ 3448, 2936, 2114, 1636, 1515, 1249, 1179, 668, 616; HRESIMS *m*/*z* 684.3736 [M + Na]^+^ (calcd. for C_37_H_51_N_5_NaO_6_^+^, 684.3732). ^1^H and ^13^C NMR data are in the Supplementary Materials.

## 4. Conclusions

In summary, carmabin A and dragomabin have been synthesized in an efficient and stereoselective fashion (52.3 mg and 43.8 mg obtained, respectively). The absolute stereochemistry at C35 and C37 of carmabin A has been assigned as 35*R, 37S* and the absolute stereochemistry at C35 of dragomabin has been revised as 35*R*. It is anticipated that this work will lead to further investigations of carmabin A and dragomabin as well as other acetylene-containing lipopeptides in both medicinal chemistry and chemical biology.

## Supplementary Materials

The following are available online at http://www.mdpi.com/1660-3397/16/9/338/s1, Figures S1–S3: NMR comparison of natural and synthetic carmabin A (**1**), Figures S4–S6: NMR comparison of natural and synthetic dragomabin (**2a**), Figures S7–S64: NMR, COSYS, HSQC, NOESY and HMBC Spectra, Tables S1 and S2: NMR data of natural and synthetic carmabin A (**1**); Tables S3 and S4: NMR data of natural and synthetic dragomabin (**2a**).
