# Supplementary material for: Total Synthesis of the Highly N-Methylated Peptides Carmabin A and Dragomabin
Source: Mar Drugs. 2018 Sep 17;16(9):338. doi: 10.3390/md16090338 (PMC6164609; doi:10.3390/md16090338)
Supplement: Supplementary file 1 [file marinedrugs-16-00338-s001.pdf]

Supporting Information

Total Synthesis of the Highly *N*-Methylated Peptides Carmabin A and Dragomabin

Baijun Ye [1], Peng Jiang [1], Tingrong Zhang [1], Yuanjun Sun [1], Xin Hao [1], Yingjun Cui [1], Liang Wang [1,*] and Yue Chen [1,2,*]

[1] The State Key Laboratory of Medicinal Chemical Biology, College of Pharmacy and Tianjin Key Laboratory of Molecular Drug Research, Nankai University, Tianjin 300350, China; yebaijunts@126.com (B.Y.), jiang1921372889@126.com (P.J.); nku2120181185@126.com (T.Z.); sunyuanjun7818@163.com (Y.S.); haoxinbit@126.com (X.H.); cyj10080@126.com (Y.C.)

[2] Collaborative Innovation Center of Chemical Science and Engineering, Tianjin 300350, PR China; yuechen@nankai.edu.cn

* Correspondence: lwang@nankai.edu.cn (L.W.); yuechen@nankai.edu.cn (Y.C.); Tel.: +86-22-85358387 (Y.C.)

Table of contents

NMR Comparison of natural and synthetic **carmabin A (1)**......................................S1

NMR Comparison of natural and synthetic **dragomabin (2a)**....................................S5

NMR Spectra S9

NMR Comparison of natural and synthetic carmabin A (1)

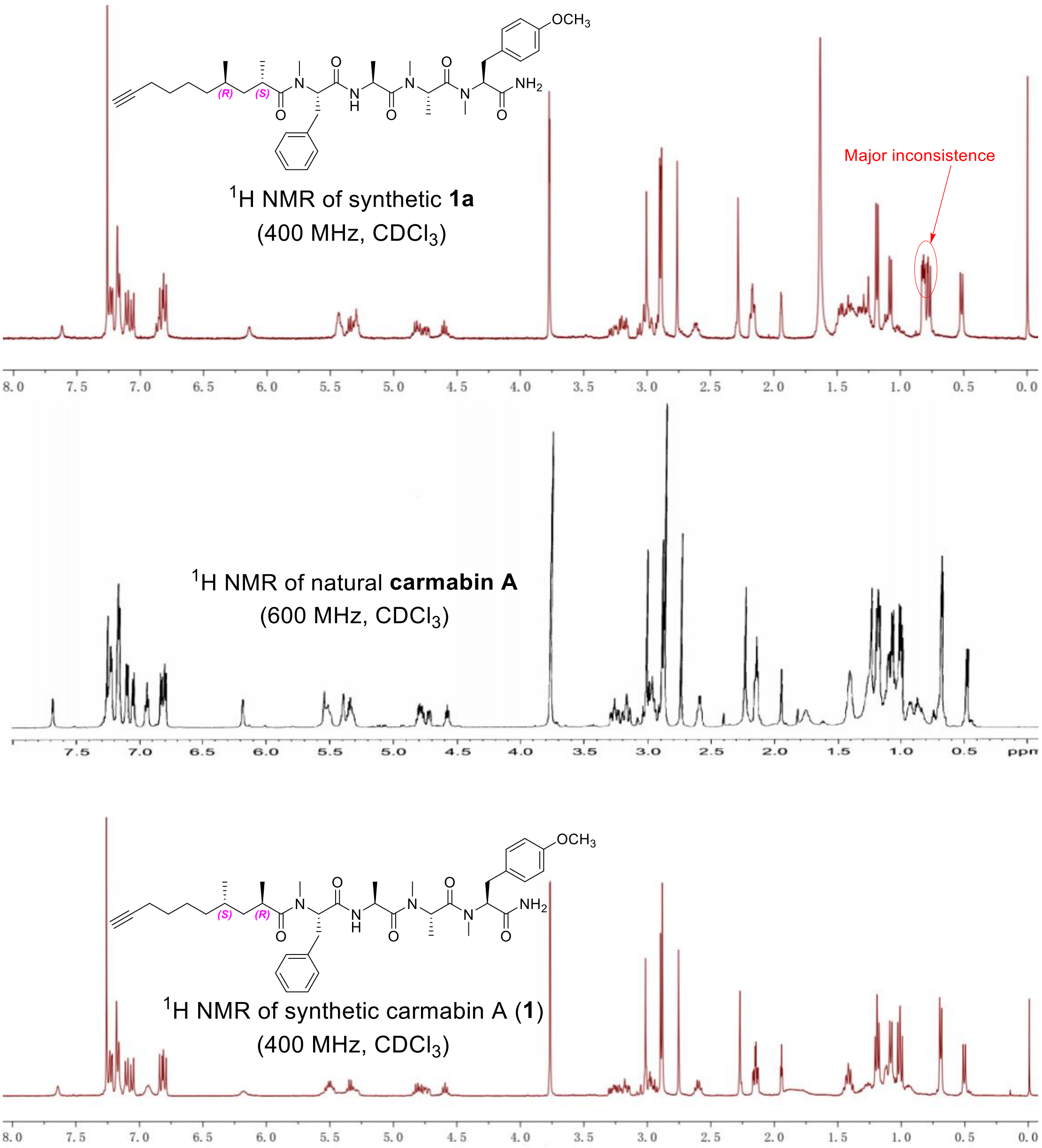

Figure S1. ^{1}H NMR comparison of natural and synthetic **carmabin A (1)**.

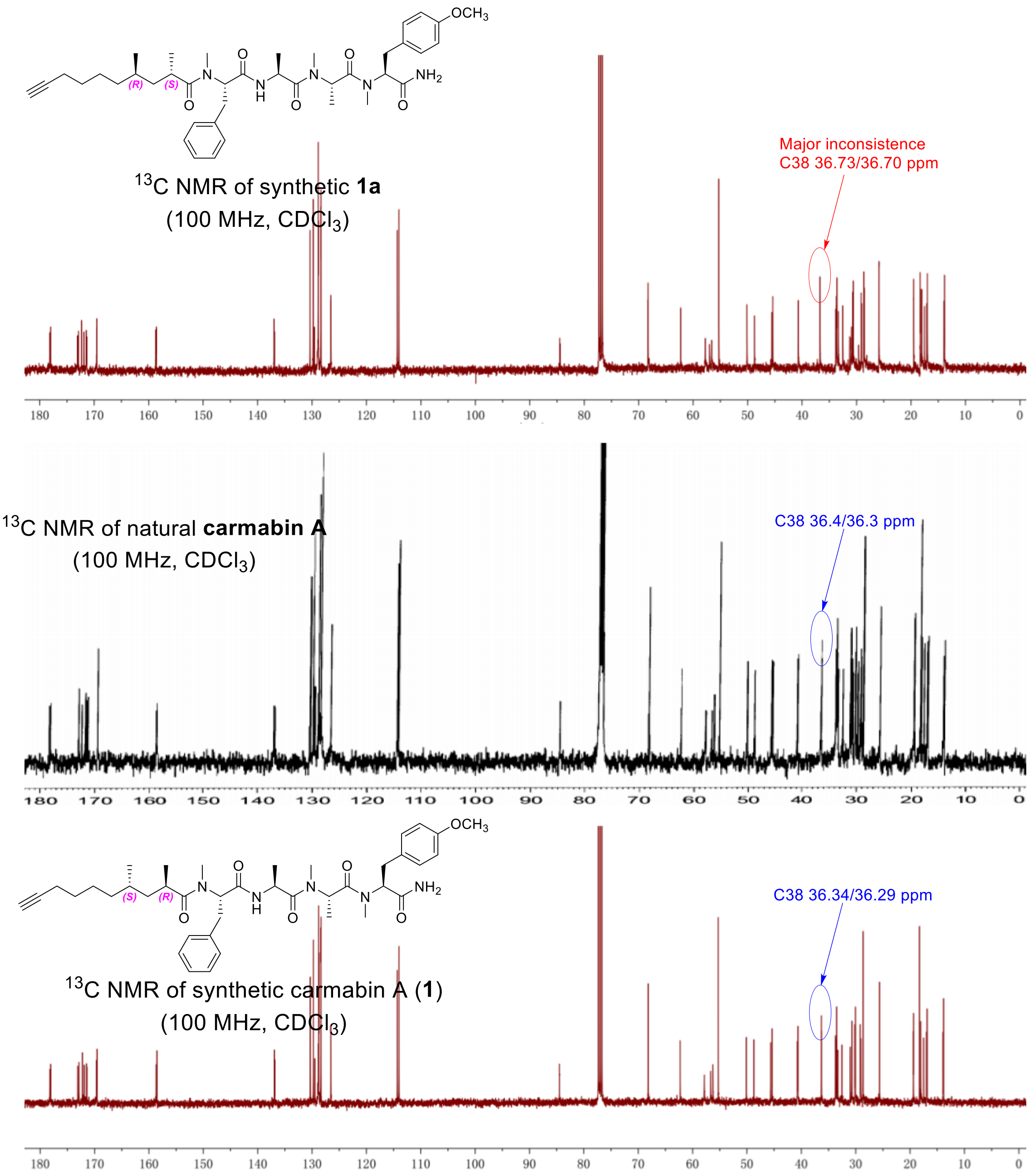

Figure S2. ^{13}C NMR comparison of natural and synthetic **carmabin A (1)**.

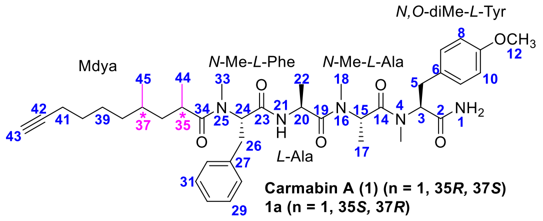

Figure S3. Carmabin A (1) and **1a** with atom numbering.

Table S1. ^{1}H NMR data of natural and synthetic **carmabin A (1)**

Unit	Position	Synthetic Carmabin A (1)		Natural Carmabin A		Synthetic 1a
		^{1}H (400 MHz, $CDCl_3$) δ_H (mult, *J* in Hz)	Δ δ (ppm)	^{1}H (600 MHz, $CDCl_3$) δ_H (mult, *J* in Hz)	Δ δ (ppm)	^{1}H (400 MHz, $CDCl_3$) δ_H (mult, *J* in Hz)
N,*O*-diMeTyr	1	7.64/6.17/5.54/5.40, br s, NH_2	-	5.54/5.40, br s, NH_2[a]	-	7.62/6.14/5.40/5.30, br s, NH_2
	2					
	3	5.32, ob/4.73, dd (10.6, 3.9), CH	0.00/0.00	5.32, ob/4.73, dd (11, 2), CH	-0.02/0.01	5.30, ob/4.74, dd (10.8, 4.0), CH
	4					
	5	3.19/3.01, ob, CH_2	-	3.19/3.16, ob, CH_2[b]	-	3.18/3.02, ob, CH_2
	6					
	7	7.10/7.06, d (8.5), CH	0.00/0.01	7.10/7.05, d (8), CH	0.00/0.01	7.10/7.06, d (8.5), CH
	8	6.83/6.80, d (8.5), CH	0.00/0.00	6.83/6.80, d (8), CH	0.00/0.01	6.83/6.81, d (8.5), CH
	9					
	10	6.83/6.80, d (8.5), CH	0.00/0.00	6.83/6.80, d (8), CH	0.00/0.01	6.83/6.81, d (8.5), CH
	11	7.10/7.06, d (8.5), CH	0.00/0.01	7.10/7.05, d (8), CH	0.00/0.01	7.10/7.06, d (8.5), CH
	12	3.77/3.76, s, CH_3	-[c]	3.77, s, CH_3	-[c]	3.77/3.76, s, CH_3
	13	2.90/2.75, s, CH_3	0.00/-0.01	2.90/2.76, s, CH_3	0.00/0.00	2.90/2.76, s, CH_3
N-MeAla	14					
	15	5.35/4.81, ob, CH	0.00/0.00	5.35/4.81, ob, CH	0.00/0.01	5.35/4.82, ob, CH
	16					
	17	1.18/0.50, d (7.1), CH_3	0.00/0.02	1.18/0.48, d (7), CH_3	0.00/0.04[d]	1.18/0.52, d (7.0), CH_3
	18	3.01, 2.27, s, CH_3	-	2.24, s, CH_3[a]	-	3.00, 2.28, s, CH_3
Ala	19					
	20	4.78, ob/4.59, p (7.0), CH	0.00/0.01	4.78/4.58, p (7), CH	0.00/0.02	4.78/4.60, p (7.0), CH
	21	6.94, br, NH	0.00	6.94, t (7), NH	-0.09	6.85, ob, NH
	22	1.20/1.08, d (6.7), CH_3	0.00/0.00	1.20/1.08, d (7), CH_3	-0.02/0.00	1.18/1.08, d (7.0), CH_3
N-MePhe	23					
	24	5.52, ob, CH	0.00	5.52, ob, CH	-0.09[d]	5.43, ob, CH
	25					
	26	3.27/2.98, ob, CH_2	-	3.28/3.25, ob, CH_2[b]	-	3.26/2.98, ob, CH_2
	27					
	28	7.18, ob, CH	0.00	7.18, ob, CH	0.00	7.18, ob, CH
	29	7.17, ob, CH	0.00	7.17, ob, CH	-0.01	7.16, ob, CH
	30	7.24, m, CH	0.00	7.24, m, CH	0.00	7.24, m, CH
	31	7.17, ob, CH	0.00	7.17, ob, CH	-0.01	7.16, ob, CH
	32	7.18, ob, CH	0.00	7.18, d (ob), CH	0.00	7.18, ob, CH
	33	2.88, s, CH_3	0.00	2.88, s, CH_3	-[c]	2.89/2.88, s, CH_3
Mdya	34					
	35	2.60, sextet (6.6), CH	0.00	2.60, sextet (6), CH	0.00	2.60, m, CH
	36	1.13/1.10, m, CH_2	0.00	1.13/1.10, m, CH_2	0.28/0.20[d]	1.41/1.30, m, CH_2
	37	1.12, m, CH	0.00	1.12, m, CH	0.26[d]	1.38, m, CH
	38	1.04/0.93, m, CH_2	-	0.93, m, CH_2[a]	-	1.24/1.11, m, CH_2
	39	1.29/1.21, m, CH_2	0.00/0.00	1.29/1.21, m, CH_2	0.07/0.06[d]	1.36/1.27, m, CH_2
	40	1.42, m, CH_2	0.00	1.42, m, CH_2	0.05[d]	1.47, m, CH_2
	41	2.15, td (7.1, 2.6), CH_2	0.00	2.15, t (6.6), CH_2	0.02	2.17, td (7.1, 2.6), CH_2
	42					
	43	1.94, t (2.5), CH	-0.01	1.95, br s, CH	-0.01	1.94, br s, CH
	44	1.02/1.00, d (7.0), CH_3	-0.05/-0.02[e]	1.07/1.02, d (6.8), CH_3	-0.28/-0.25[d]	0.79/0.77, d (6.9), CH_3
	45	0.68, d (6.2), CH_3	0.00	0.68, d (6.1), CH_3	-	0.82/0.81, d (6.0), CH_3

[a] The assignment of chemical shifts of natural **carmabin A** is incomplete according to the corresponding ^{1}H NMR and 2D NMR spectra.
[b] The assignment of chemical shifts of natural **carmabin A** is inaccurate according to the corresponding ^{1}H NMR and 2D NMR spectra.
[c] The difference of splitting pattern probably caused by rotamers.
[d] Obvious difference in the chemical shifts could be observed between the natural **carmabin A** and synthetic **1a** labelled in red.
[e] The difference of chemical shifts probably caused by impurities in natural **carmabin A**.

Table S2. ^{13}C NMR data of natural and synthetic **carmabin A (1)**[a]

Unit	Position	Synthetic Carmabin A (1)		Natural Carmabin A		Synthetic 1a
		^{13}C (100 MHz, $CDCl_3$)	Δ δ (ppm)	^{13}C (100 MHz, $CDCl_3$)	Δ δ (ppm)	^{13}C (100 MHz, $CDCl_3$)
N,*O*-diMeTyr	1					
	2	171.9/171.5, qC	0.20/0.10	171.7/171.4, qC	0.20/0.00	171.9/171.4, qC
	3	62.3/57.8, CH	0.00/-0.10	62.3/57.9, CH	0.00/-0.10	62.3/57.8, CH
	4	N	-	N	-	N
	5	33.3/32.5, CH_2	-0.20/0.00	33.5/32.5, CH_2	-0.20/0.00	33.3/32.5, CH_2
	6	129.5, qC	-0.10	129.6, qC	0.00	129.6, qC
	7	130.3/129.8, CH	-0.10/0.00	130.4/129.8, CH	0.00/0.00	130.4/129.8, CH
	8	114.3/114.0, CH	-0.10/-0.10	114.4/114.1, CH	0.00/-0.10	114.4/114.0, CH
	9	158.7/158.5, qC	0.00/-0.10	158.7/158.6, qC	0.00/0.00	158.7/158.6, qC
	10	114.3/114.0, CH	-0.10/-0.10	114.4/114.1, CH	0.00/-0.10	114.4/114.0, CH
	11	130.3/129.8, CH	-0.10/0.00	130.4/129.8, CH	0.00/0.00	130.4/129.8, CH
	12	55.3, CH_3	0.00	55.3, CH_3	0.00	55.3, CH_3
	13	31.0/29.0, CH_3	0.00/0.00	31.0/29.0, CH_3	0.00/0.00	31.0/29.0, CH_3
N-MeAla	14	173.07/172.23, qC	0.09/-0.17	172.98/172.4, qC	0.09/-0.1	173.07/172.30, qC
	15	50.1/48.7, CH	-0.10/-0.10	50.2/48.8, CH	-0.10/-0.10	50.1/48.7, CH
	16	N	-	N	-	N
	17	14.0/13.9, CH_3	0.00/0.00	14.0/13.9, CH_3	0.00/0.00	14.0/13.9, CH_3
	18	30.7/29.2, CH_3	-[b]	29.2, CH_3	-[b]	30.68/29.15, CH_3
Ala	19	171.4, qC	0.10	171.3, qC	0.00	171.3, qC
	20	45.6/45.4, CH	-0.10/0.00	45.7/45.4, CH	-0.10/0.00	45.6/45.4, CH
	21	NH	-	NH	-	NH
	22	18.0/17.5, CH_3	-0.10/-0.20	18.1/17.7, CH_3	-0.10/-0.20	18.0/17.5, CH_3
N-MePhe	23	169.65/169.56 qC	-[b]	169.5, qC	-[b]	169.54/169.49 qC
	24	56.7/56.3, CH	0.00/0.00	56.7/56.3, CH	0.30/0.30[c]	57.0/56.6, CH
	25	N	-	N	-	N
	26	33.7/33.55, CH_2	-[b]	33.7, CH_2	-[b]	33.83/33.68, CH_2
	27	136.92/136.85, qC	-0.08/-0.05	137.0/136.9, qC	0.00/0.00	137.0/136.9, qC
	28	128.86/128.79, CH	0.00/-0.01	128.86/128.80, CH	-0.01/0.01	128.85/128.81, CH
	29	126.54/126.52, CH	-0.06/-0.06	126.60/126.58, CH	-0.06/-0.07	126.54/126.51, CH
	30	128.37/128.35, CH	-[b]	128.4, CH	-[b]	128.36/128.33, CH
	31	126.54/126.52, CH	-0.06/-0.06	126.60/126.58, CH	-0.06/-0.07	126.54/126.51, CH
	32	128.86/128.79, CH	0.00/-0.01	128.86/128.80, CH	-0.01/0.01	128.85/128.81, CH
	33	30.99, CH_3	0.19	30.8, CH_3	0.21	31.01, CH_3
Mdya	34	178.2/178.1, qC	-0.01/0.00	178.3/178.1, qC	-0.10/-0.10	178.2/178.0, qC
	35	33.52/33.49, CH	-0.08/-0.06	33.60/33.55, CH	0.06/0.07	33.66/33.62, CH
	36	40.7/40.6, CH_2	-0.10/-0.10	40.8/40.7, CH_2	-0.08/-0.02	40.72/40.68, CH_2
	37	30.14/30.07, CH	-[b]	30.2, CH	0.40[c]	30.6, CH
	38	36.34/36.29, CH_2	-0.06/-0.01	36.4/36.3, CH_2	0.33/0.40[c]	36.73/36.70, CH_2
	39	25.6, CH_2	-0.10	25.7, CH_2	-[b]	25.90/25.89, CH_2
	40	28.7, CH_2	0.00	28.7, CH_2	-[b]	28.67/28.58, CH_2
	41	18.3, CH_2	0.00	18.3, CH_2	-[b]	18.31/18.27, CH_2
	42	84.5, qC	-0.10	84.6, qC	-[b]	84.5/84.4, qC
	43	68.2, CH	0.00	68.2, CH	-[b]	68.32/68.27, CH
	44	17.0/16.9, CH_3	-0.10/-0.10	17.1/17.0, CH_3	0.00/0.00	17.1/17.0, CH_3
	45	19.41/19.38, CH_3	-0.09/-0.07	19.50/19.45, CH_3	0.01/0.05	19.51/19.50, CH_3

[a] ^{13}C NMR spectra were calibrated by using internal references and solvent signals $CDCl_3$ (δ_C =77.00 ppm).

[b] The difference of splitting pattern probably caused by rotamers.

[c] Obvious difference in the chemical shifts could be observed between the natural **carmabin A** and Synthetic **1a** labelled in red.

NMR Comparison of natural and synthetic dragomabin (2a)

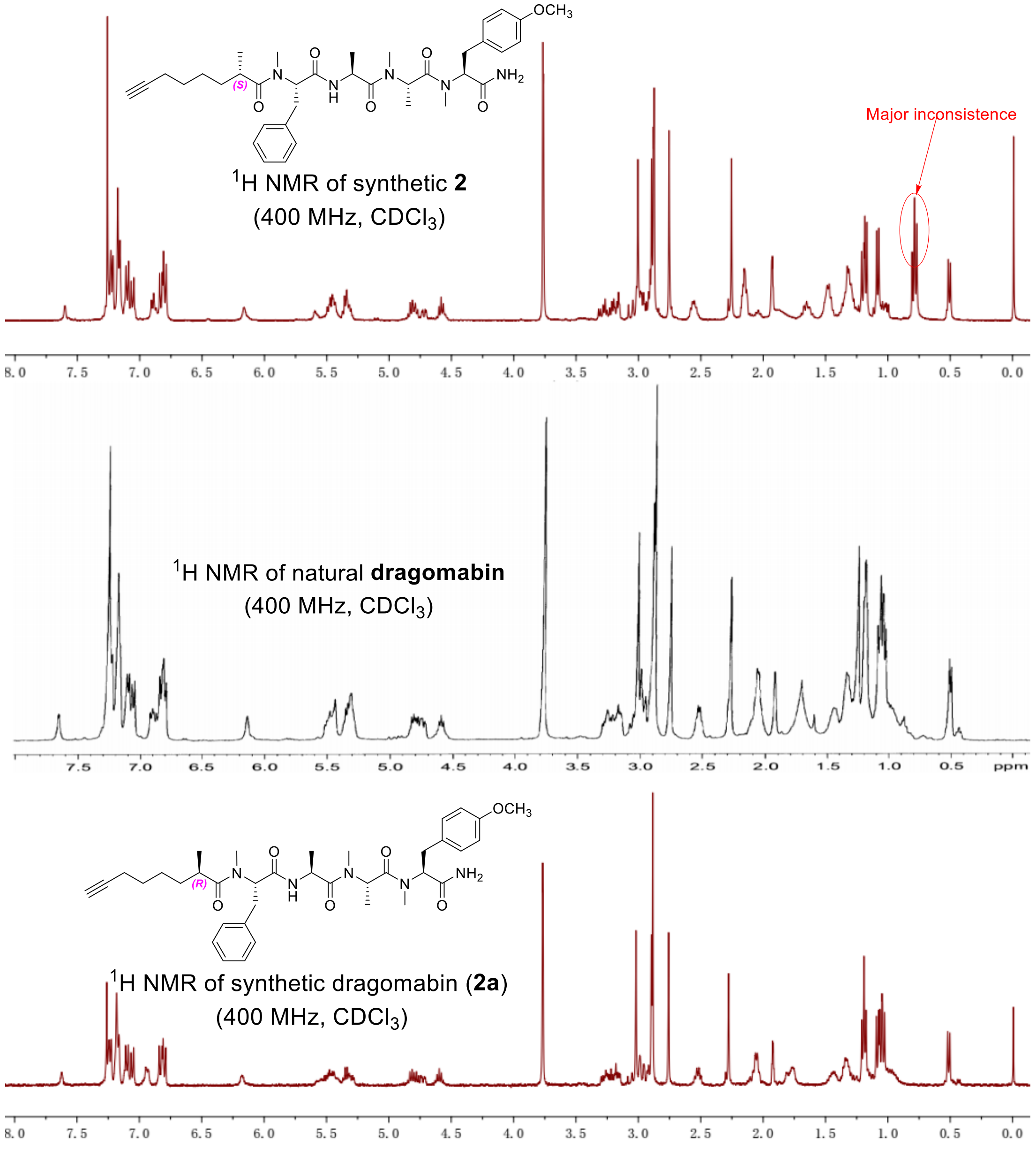

Figure S4. ^{1}H NMR comparison of natural and synthetic **dragomabin (2a)**.

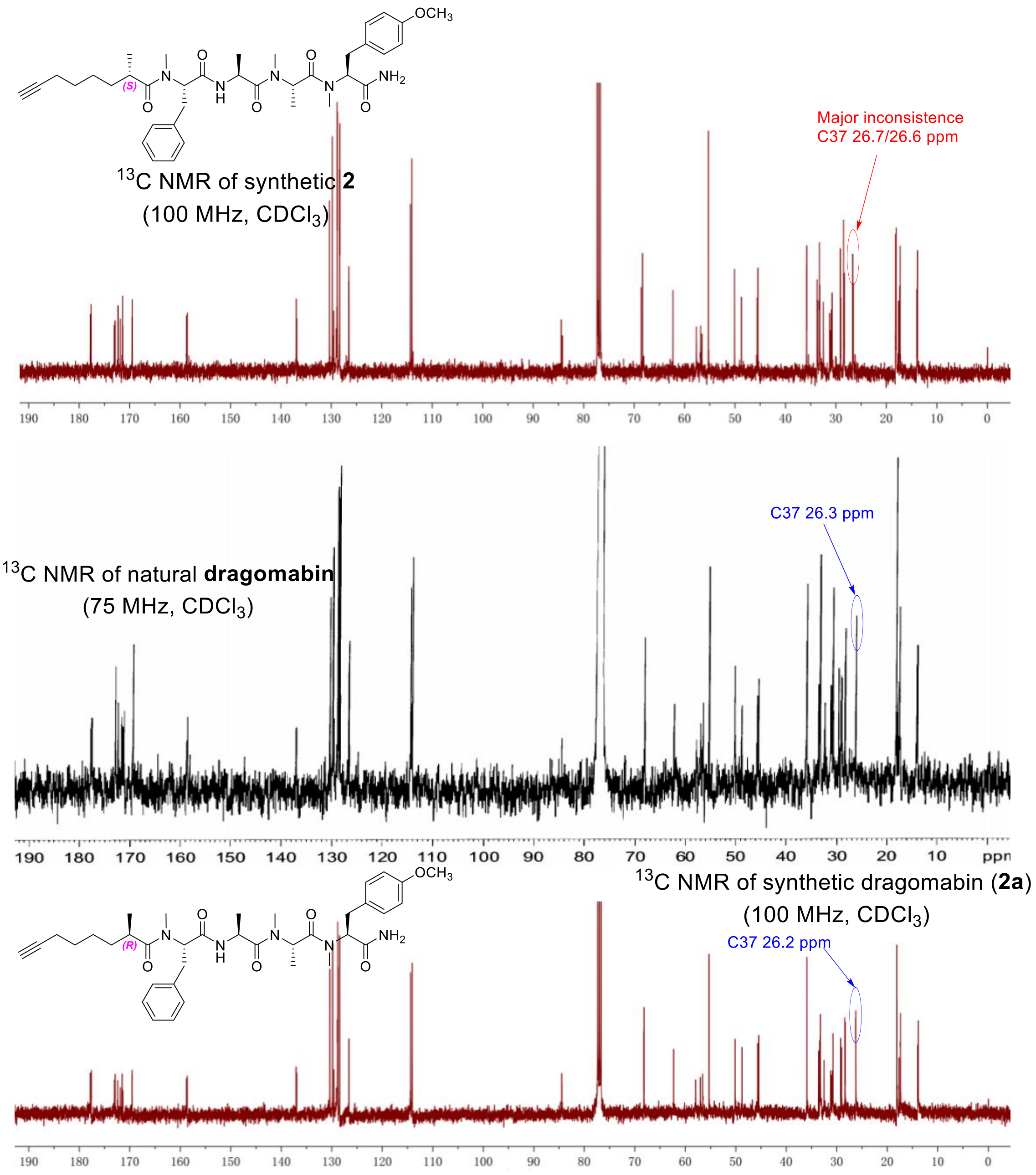

Figure S5. ^{13}C NMR comparison of natural and synthetic **dragomabin (2a)**.

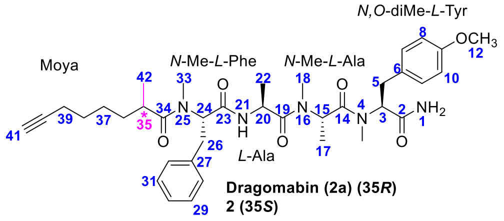

Figure S6. Dragomabin (2a) and **2** with atom numbering.

Table S3. ^{1}H NMR data of natural and synthetic **dragomabin (2a)**

Unit	Position	Synthetic Dragomabin (2a)		Natural Dragomabin		Synthetic 2
		^{1}H (400 MHz, $CDCl_3$) δ_H (mult, *J* in Hz)	Δ δ (ppm)	^{1}H (400 MHz, $CDCl_3$) δ_H (mult, *J* in Hz)	Δ δ (ppm)	^{1}H (400 MHz, $CDCl_3$) δ_H (mult, *J* in Hz)
N,*O*-diMeTyr	1	7.62/6.18/5.55/5.41, br s, NH_2	-	5.44, br s, NH_2[a]	-	7.60/6.16/5.59/5.43, br s, NH_2
	2					
	3	5.30, ob/4.72, ob, CH	-0.02/-0.02	5.32, ob/4.74, ob, CH	0.00/-0.02	5.32, ob/4.72, dd (10.7, 3.8) , CH
	4					
	5	3.19/3.01, m, CH_2	-	3.17, m, CH_2[b]	-	3.19/3.01, m, CH_2
	6					
	7	7.10/7.06, d (8.5) , CH	0.00/0.00	7.10/7.06, d (8) , CH	0.00/0.00	7.10/7.06, d (8.5) , CH
	8	6.83/6.80, d (8.7) , CH	0.00/0.00	6.83/6.80, d (8) , CH	0.00/0.00	6.83/6.80, d (8.6) , CH
	9					
	10	6.83/6.80, d (8.7) , CH	0.00/0.00	6.83/6.80, d (8) , CH	0.00/0.00	6.83/6.80, d (8.6) , CH
	11	7.10/7.06, d (8.5) , CH	0.00/0.00	7.10/7.06, d (8) , CH	0.00/0.00	7.10/7.06, d (8.5) , CH
	12	3.77/3.76, s, CH_3	-[c]	3.77, s, CH_3	-[c]	3.77/3.76, s, CH_3
	13	2.90/2.76, s, CH_3	0.00/0.00	2.90/2.76, s, CH_3	-0.01/-0.01	2.89/2.75, s, CH_3
N-MeAla	14					
	15	5.34/4.81, ob, CH	-0.01/0.00	5.35/4.81, ob, CH	0.00/0.00	5.35/4.81, ob, CH
	16					
	17	1.18/0.51, d (7.1), CH_3	0.00/0.00	1.18/0.51, d (7), CH_3	0.00/0.00	1.18/0.51, d (7.1), CH_3
	18	3.02/2.28, s, CH_3	-	2.28, s, CH_3[a]	-	3.00/2.25, s, CH_3
Ala	19					
	20	4.78, ob/4.60, p (7.1) , CH	0.00/0.00	4.78/4.60, p (7) , CH	0.00/-0.02	4.78, ob/4.58, p (6.9) , CH
	21	6.95, br, NH	0.05	6.90, ob, NH	-0.01	6.89, br, NH
	22	1.20/1.08, d (6.9), CH_3	0.00/0.00	1.20/1.08, ob, CH_3	0.00/0.00	1.20/1.08, d (6.9), CH_3
N-MePhe	23					
	24	5.49, m, CH	-0.01	5.50, m, CH	-0.01	5.49, m, CH
	25					
	26	3.25/2.97, m, CH_2	-	3.28/3.25, m, CH_2[b]	-	3.28/2.96, m, CH_2
	27					
	28	7.18, ob, CH	0.00	7.18, d (ob), CH	0.00	7.18, ob, CH
	29	7.17, ob, CH	0.00	7.17, ob, CH	0.00	7.17, ob, CH
	30	7.24, m, CH	0.00	7.24, m, CH	0.00	7.24, m, CH
	31	7.17, ob, CH	0.00	7.17, ob, CH	0.00	7.17, ob, CH
	32	7.18, ob, CH	0.00	7.18, d (ob) , CH	0.00	7.18, ob, CH
	33	2.88, s, CH_3	-0.01	2.89, s, CH_3	-[c]	2.88/2.87, s, CH_3
Moya	34					
	35	2.53, sextet (6.1), CH	0.00	2.53, sextet (6), CH	0.02	2.55, sextet (6), CH
	36	1.45/1.18, m, CH_2	-0.01/-0.02	1.46/1.20, m, CH_2	0.18/0.09[d]	1.64/1.29, m, CH_2
	37	1.00, m, CH_2	0.00	1.00, m, CH_2	0.32[d]	1.32, m, CH_2
	38	1.34, m, CH_2	0.00	1.34, m, CH_2	0.14[d]	1.48, m, CH_2
	39	2.06, m, CH_2	0.00	2.06, m, CH_2	-0.02	2.04, m, CH_2
	40					
	41	1.93, br s, CH	0.00	1.93, br s, CH	0.00	1.93, br s, CH
	42	1.06/1.03, d (7.0), CH_3	-[e]	1.04, d (7), CH_3	-[d]	0.80/0.71, d (7.1), CH_3

[a] The assignment of chemical shifts of **natural dragomabin** is incomplete according to the corresponding ^{1}H NMR spectra.
[b] The assignment of chemical shifts of **natural dragomabin** is inaccurate according to the corresponding 2D NMR spectra.
[c] The difference of splitting pattern probably caused by rotamers.
[d] Obvious difference in the chemical shifts could be observed between the **natural dragomabin** and synthetic **2** labelled in red.
[e] The difference probably caused by impurities in natural **natural dragomabin**.

Table S4. ^{13}C NMR data of natural and synthetic **dragomabin (2a)** [a]

Unit	Position	Synthetic Dragomabin (2a)		Natural Dragomabin		Synthetic 2
		^{13}C (100 MHz, $CDCl_3$)	Δ δ (ppm)	^{13}C (75 MHz, $CDCl_3$)	Δ δ (ppm)	^{13}C (100 MHz, $CDCl_3$)
N,*O*-diMeTyr	1	NH_2	-	NH_2	-	NH_2
	2	171.8/171.5, qC	0.10/0.00	171.7/171.5, qC	0.20/-0.10	171.9/171.4, qC
	3	62.3/57.9, CH	0.00/0.00	62.3/57.9, CH	0.00/-0.20	62.3/57.7, CH
	4	N	-	N	-	N
	5	33.28/32.5, CH_2	-0.02/0.10	33.3/32.4, CH_2	-0.02/0.10	33.28/32.5, CH_2
	6	129.6, qC	0.00	129.6, qC	-0.10	129.5, qC
	7	130.4/129.8, CH	0.00/0.00	130.4/129.8, CH	0.00/0.00	130.4/129.8, CH
	8	114.4/114.1, CH	0.00/0.00	114.4/114.1, CH	-0.10/-0.10	114.3/114.0, CH
	9	158.8/158.6, qC	0.01/0.00	158.7/158.6, qC	0.00/0.00	158.7/158.6, qC
	10	114.4/114.1, CH	0.00/0.00	114.4/114.1, CH	-0.10/-0.10	114.3/114.0, CH
	11	130.4/129.8, CH	0.00/0.00	130.4/129.8, CH	0.00/0.00	130.4/129.8, CH
	12	55.3, CH_3	-0.10	55.4, CH_3	-0.10	55.3, CH_3
	13	31.1/29.1, CH_3	0.00/0.00	31.1/29.1, CH_3	0.00/0.00	31.1.9/29.1, CH_3
N-MeAla	14	172.9/172.4, qC	0.00/0.00	172.9/172.4, qC	0.00/-0.10	172.9/172.3, qC
	15	50.2/48.8, CH	0.00/0.00	50.2/48.8, CH	-0.10/-0.10	50.1/48.7, CH
	16	N	N	N	N	N
	17	14.0/13.9, CH_3	0.00/0.00	14.0/13.9, CH_3	0.00/0.00	14.0/13.9, CH_3
	18	30.8/29.2, CH_3	-[b]	29.2, CH_3	-[b]	30.8/29.1, CH_3
Ala	19	171.3, qC	0.00/0.00	171.3, qC	0.00/0.00	171.3, qC
	20	45.7/45.4, CH	0.00/-0.10	45.7/45.5, CH	-0.10/0.00	45.6/45.5, CH
	21	NH	-	NH	-	NH
	22	18.1/17.7, CH_3	-0.10/-0.10	18.2/17.8, CH_3	-0.10/-0.20	18.1/17.6, CH_3
N-MePhe	23	169.54/169.52, qC	-[b]	169.5, qC	-[b]	169.5/169.4, qC
	24	57.0/56.5, CH	0.00/0.00	57.0/56.5, CH	-0.20/0.10	56.8/56.6, CH
	25	N	-	N	-	N
	26	33.6/33.4, CH_2	-[b]	33.6, CH_2	-[b]	33.7/33.6, CH_2
	27	137.1/137.0, qC	0.00/0.00	137.1/137.0, qC	-0.10/-0.10	137.0/136.9, qC
	28	128.83/128.78, CH	-0.02/-0.02	128.85/128.80, CH	-0.01/0.00	128.84/128.80, CH
	29	126.61/126.58, CH	-[b]	126.6, CH	-[b]	126.55/126.51, CH
	30	128.44/128.42, CH	-[b]	128.5, CH	-[b]	128.36/128.33, CH
	31	126.61/126.58, CH	-[b]	126.6, CH	-[b]	126.55/126.51, CH
	32	128.83/128.78, CH	-0.02/-0.02	128.85/128.80, CH	-0.01/0.00	128.84/128.80, CH
	33	31.2/30.85, CH_3	-[b]	30.8, CH_3	-[b]	31.2/30.89, CH_3
Moya	34	177.8/177.6, qC	0.00/0.00	177.8/177.6, qC	0.00/0.00	177.8/177.6, qC
	35	35.9, CH	0.00/0.00	35.9, CH	-[b]	35.84/35.80, CH
	36	33.37/33.23, CH_2	0.04/-0.02	33.33/33.25, CH_2	-0.03/-0.05	33.30/33.20, CH_2
	37	26.2, CH_2	-0.10	26.3, CH_2	-[c]	26.7/26.6, CH_2
	38	28.39/28.35, CH_2	-0.01/-0.01	28.40/28.36, CH_2	0.09/-0.02	28.49/28.34, CH_2
	39	18.1/17.7, CH_2	-0.10/-0.10	18.2/17.8, CH_2	0.00/0.38	18.2/18.18, CH_2
	40	84.46/84.41, qC	-0.03/-0.04	84.49/84.45, qC	-0.06/-0.24	84.43/84.21, qC
	41	68.23/68.17, CH	-0.01/-0.01	68.24/68.18, CH	0.38/0.13[c]	68.62/68.31, CH
	42	17.4, CH_3	0.00	17.4, CH_3	-[b]	17.4/17.3, CH_3

[a] ^{13}C NMR spectra were calibrated by using internal references and solvent signals $CDCl_3$ (δ_C =77.00 ppm).

[b] The difference of splitting pattern probably caused by rotamers.

[c] Obvious difference in the chemical shifts could be observed between the **natural dragomabin** and Synthetic **2** labelled in red.

NMR Spectra

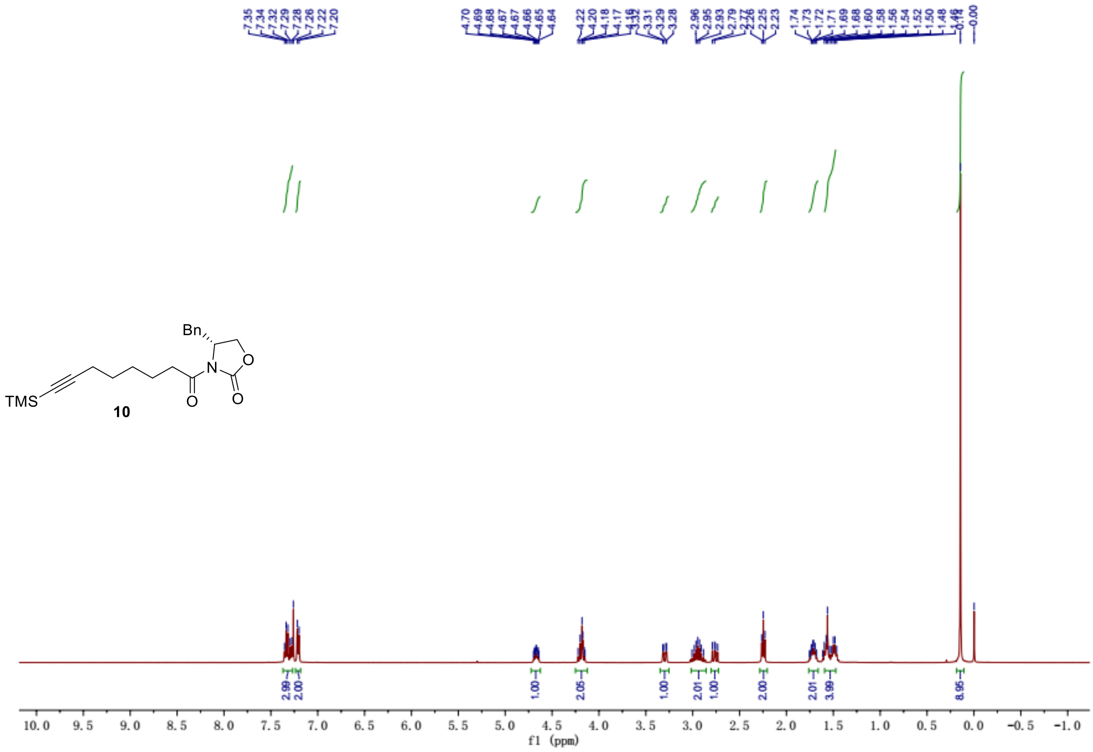

Figure S7. ^{1}H NMR (400 MHz, $CDCl_3$) spectrum of compound **10**.

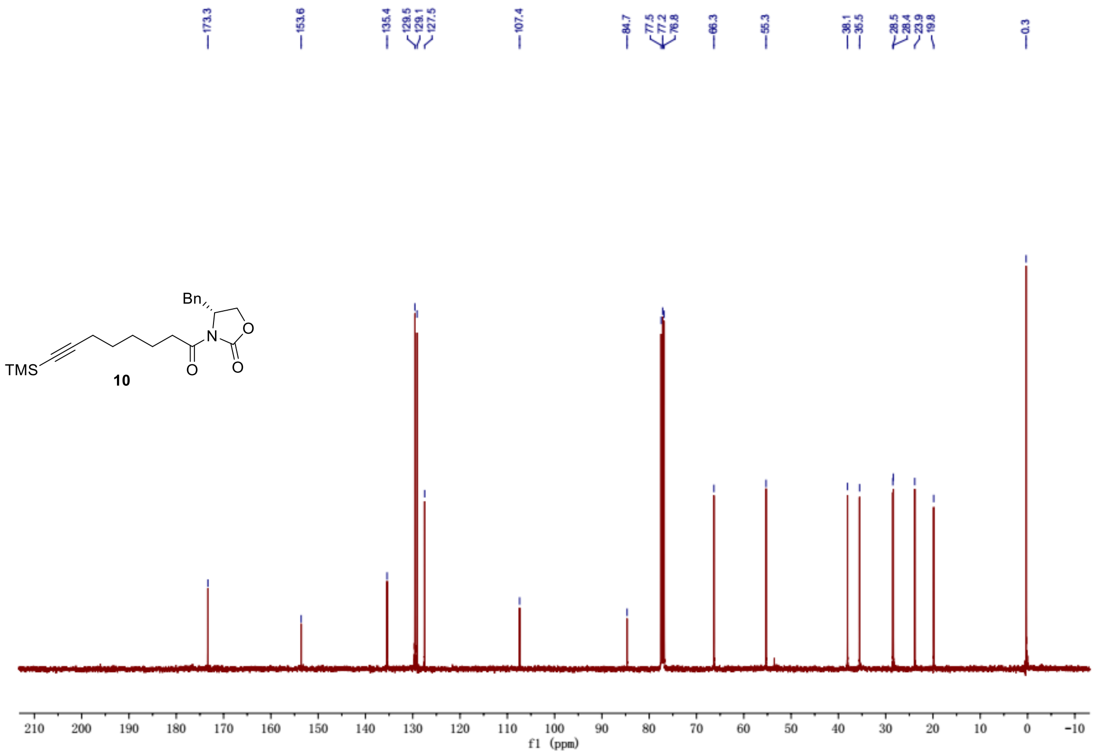

Figure S8. ^{13}C NMR (100 MHz, $CDCl_3$) spectrum of compound **10**.

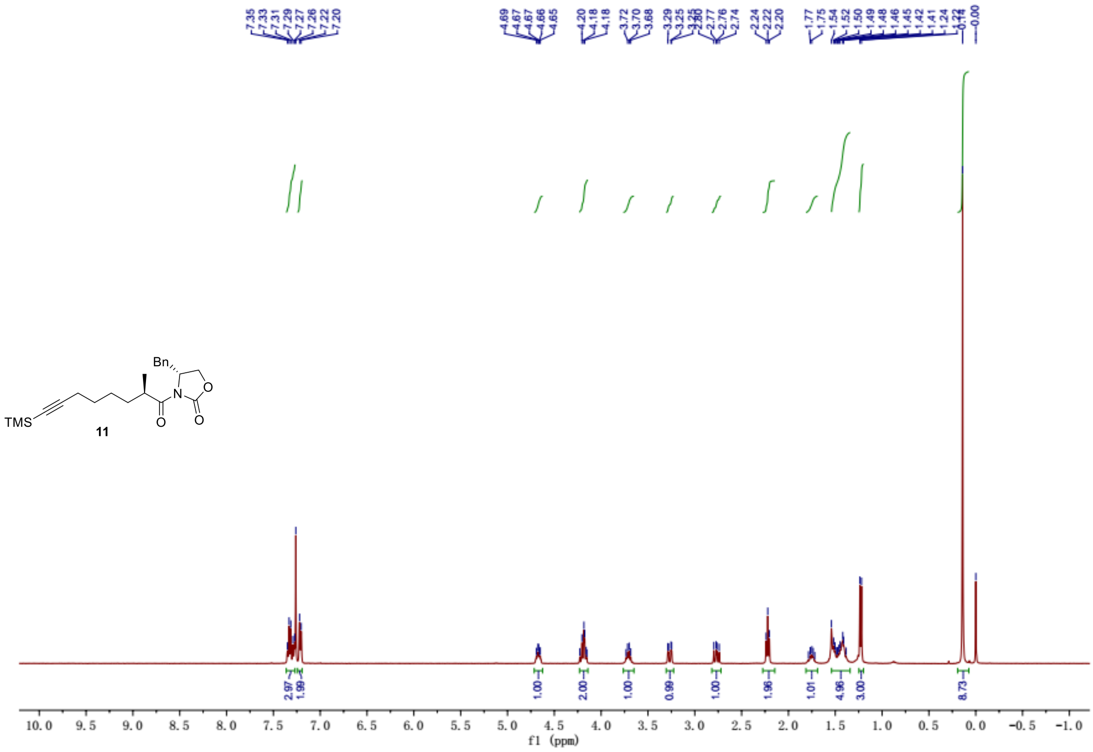

Figure S9. ^{1}H NMR (400 MHz, $CDCl_3$) spectrum of compound **11**.

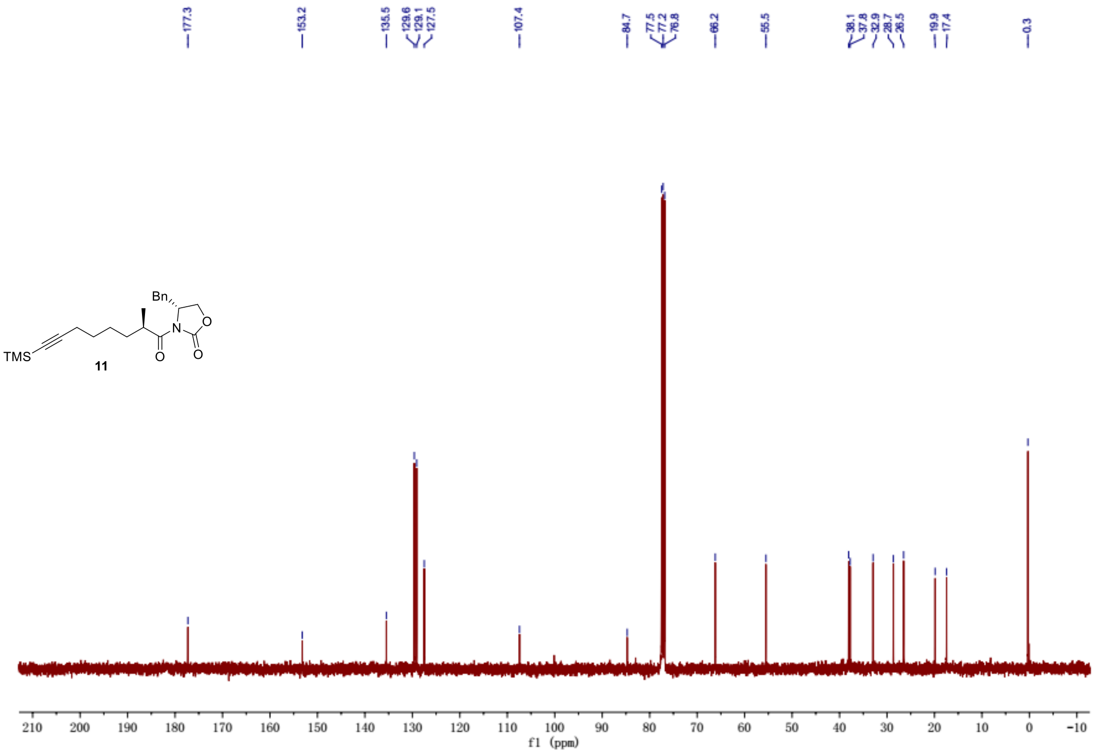

Figure S10. ^{13}C NMR (100 MHz, $CDCl_3$) spectrum of compound **11**.

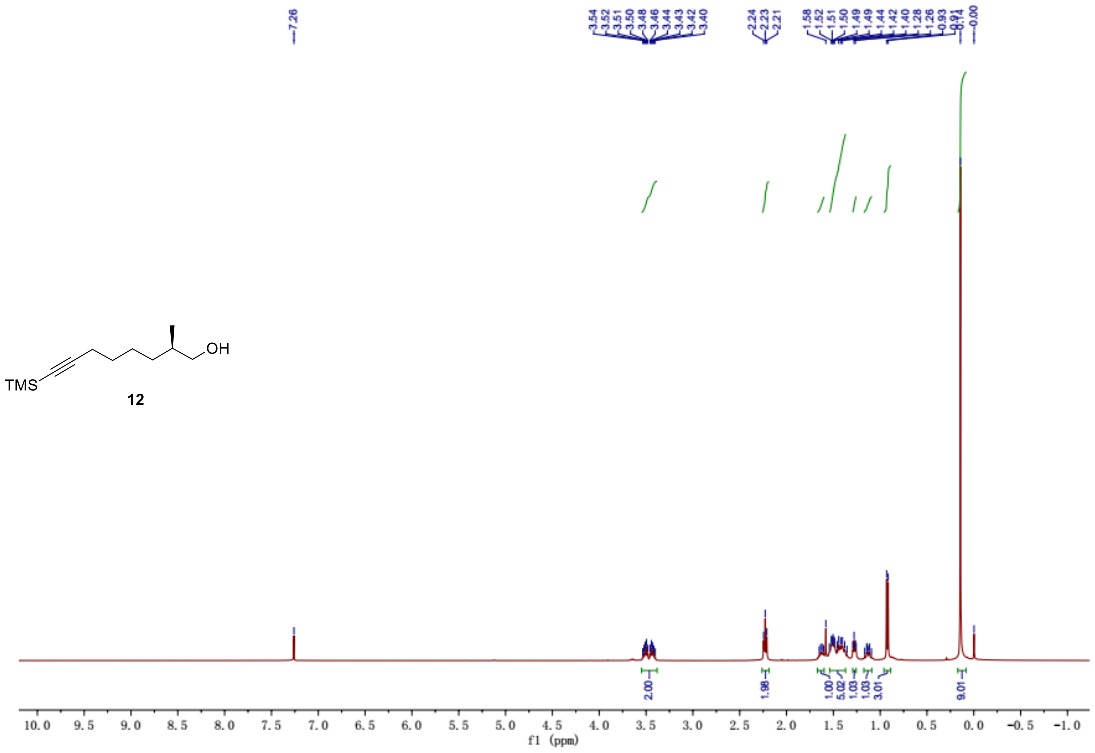

Figure S11. ^{1}H NMR (400 MHz, $CDCl_3$) spectrum of compound **12**.

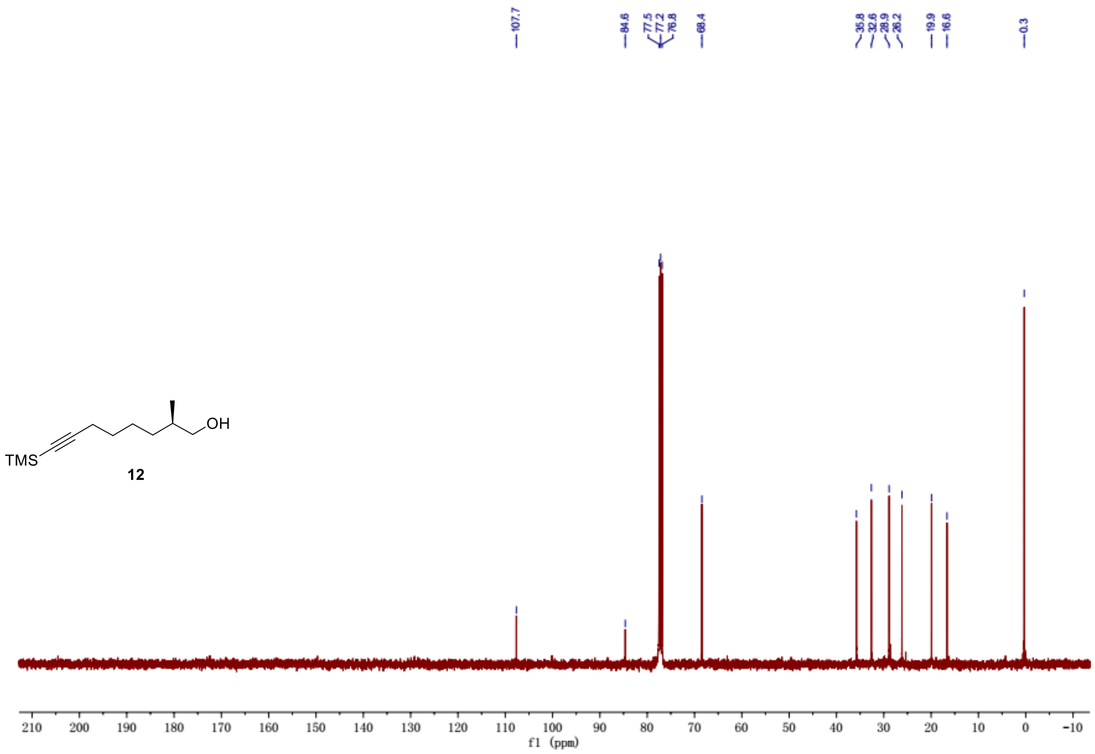

Figure S12. ^{13}C NMR (100 MHz, $CDCl_3$) spectrum of compound **12**.

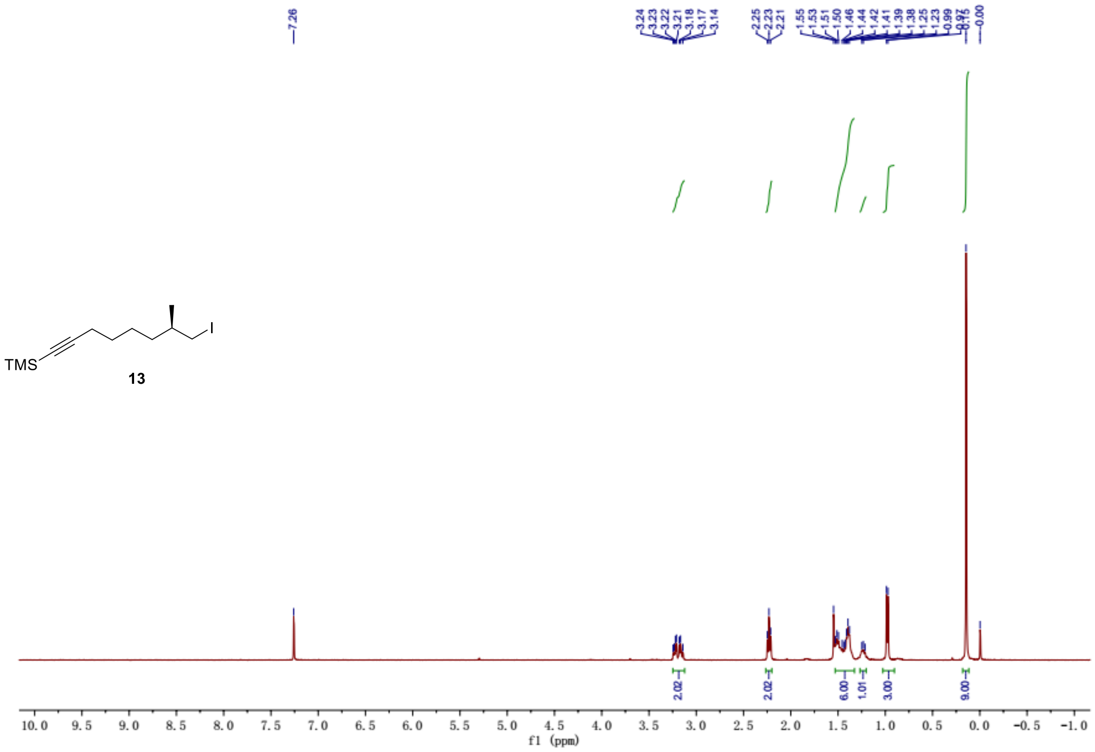

Figure S13. ^{1}H NMR (400 MHz, $CDCl_3$) spectrum of compound **13**.

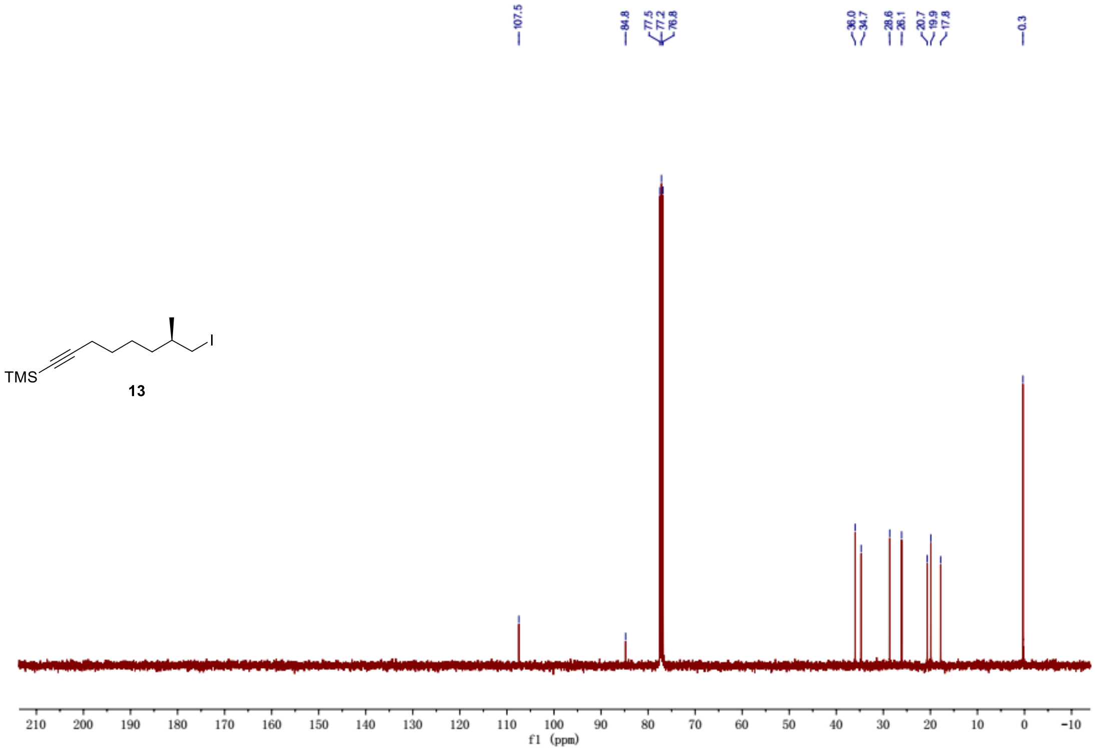

Figure S14. ^{13}C NMR (100 MHz, $CDCl_3$) spectrum of compound **13**.

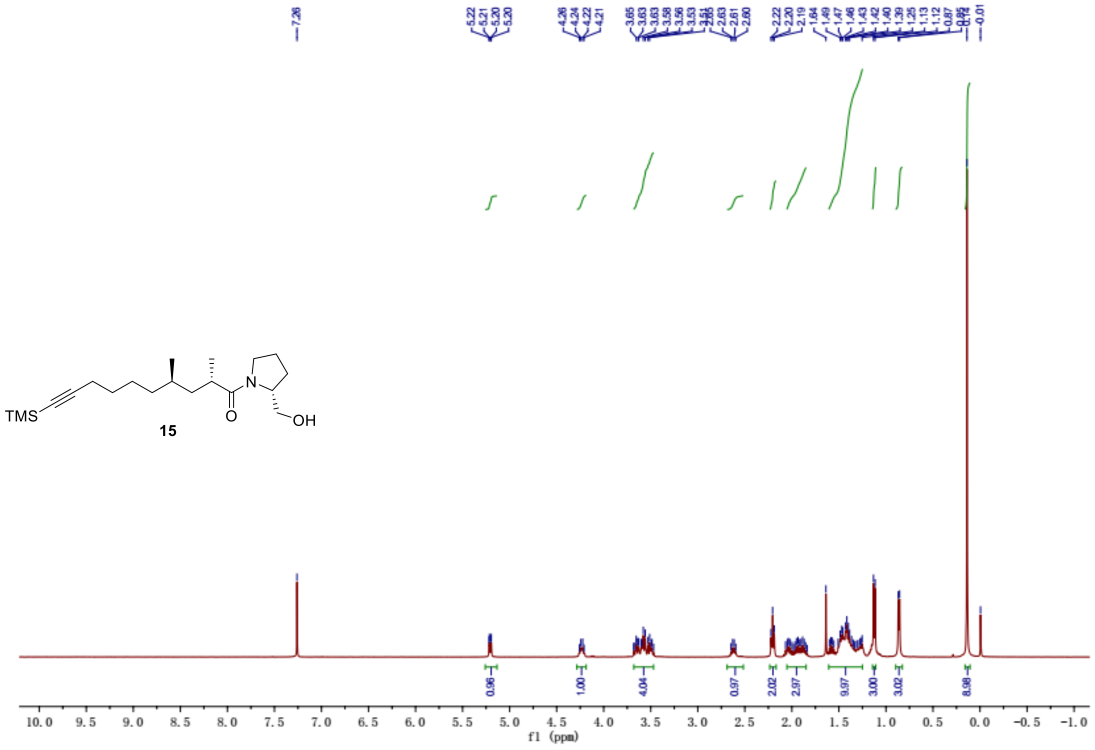

Figure S15. ^{1}H NMR (400 MHz, $CDCl_3$) spectrum of compound **15**.

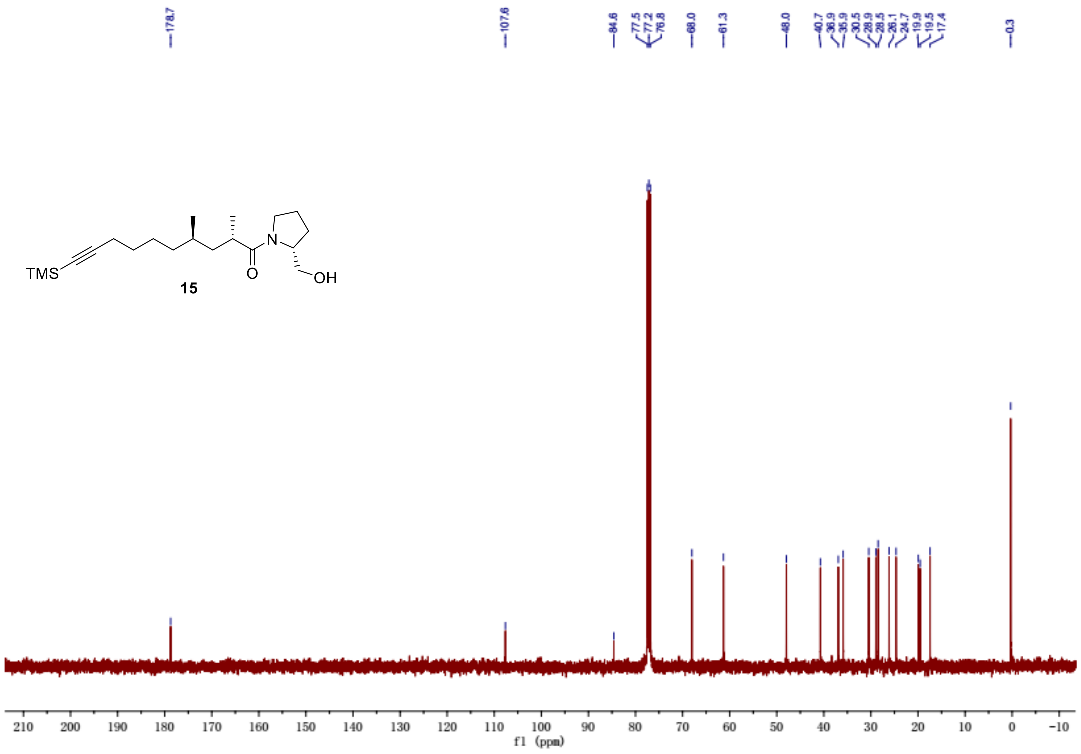

Figure S16. ^{13}C NMR (100 MHz, $CDCl_3$) spectrum of compound **15**.

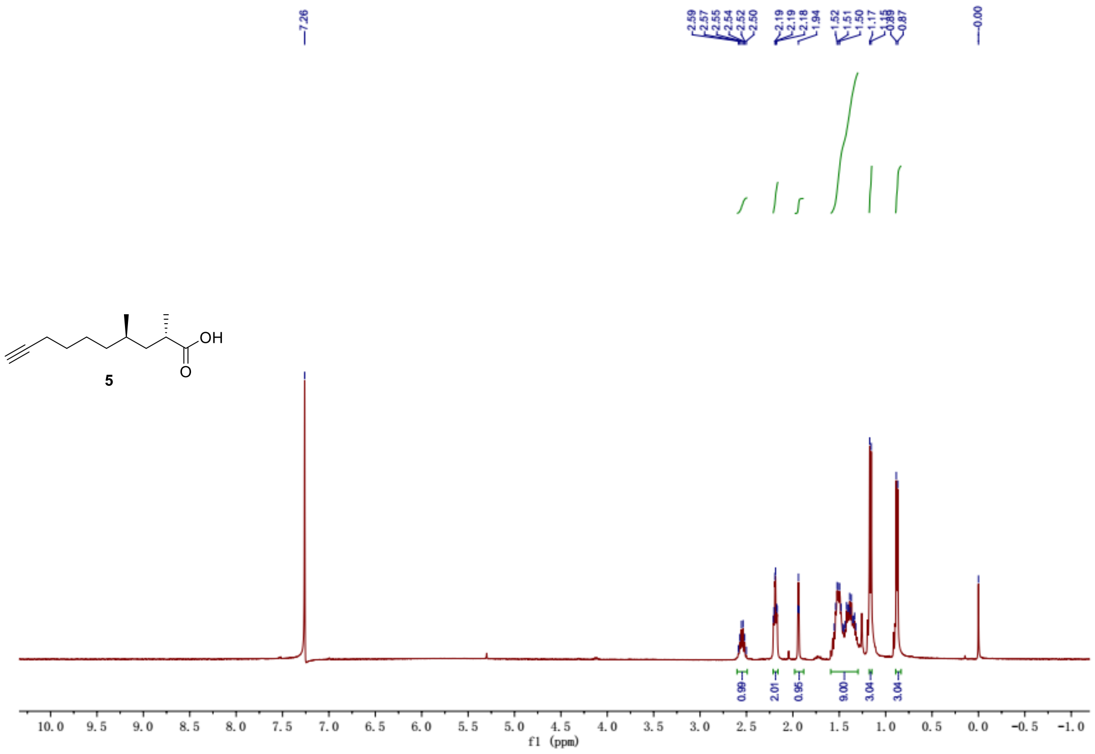

Figure S17. ^{1}H NMR (400 MHz, $CDCl_3$) spectrum of compound **5**.

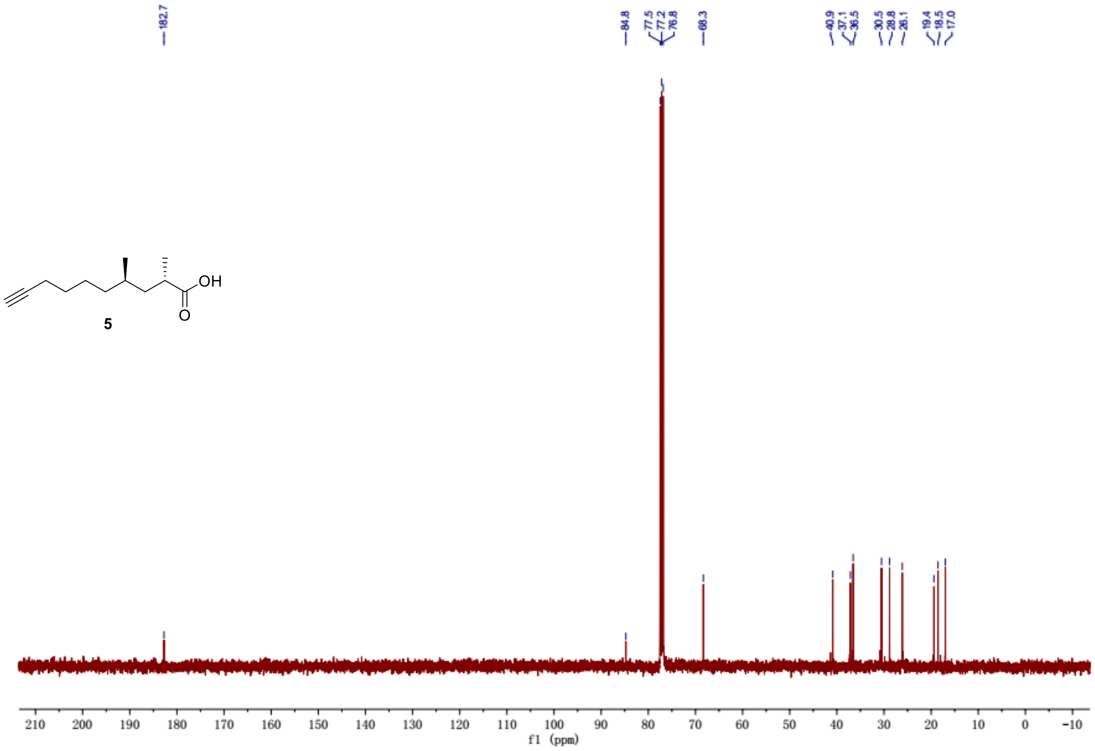

Figure S18. ^{13}C NMR (100 MHz, $CDCl_3$) spectrum of compound **5**.

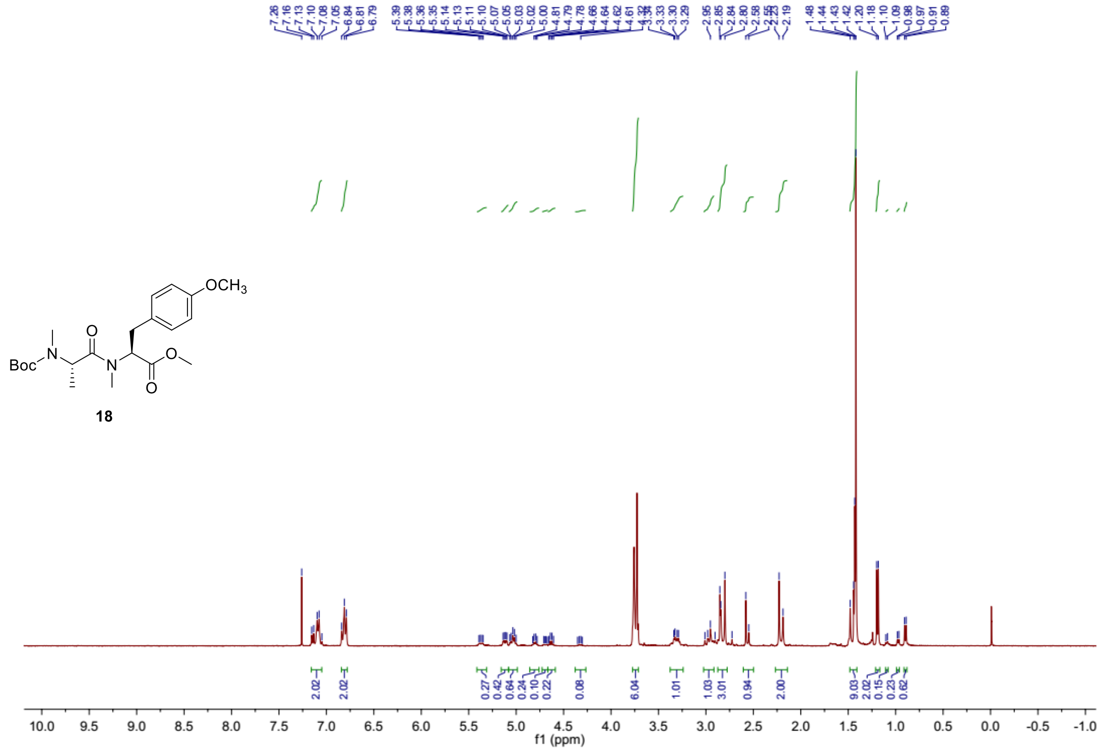

Figure S19. ^{1}H NMR (400 MHz, $CDCl_3$) spectrum of compound **18**.

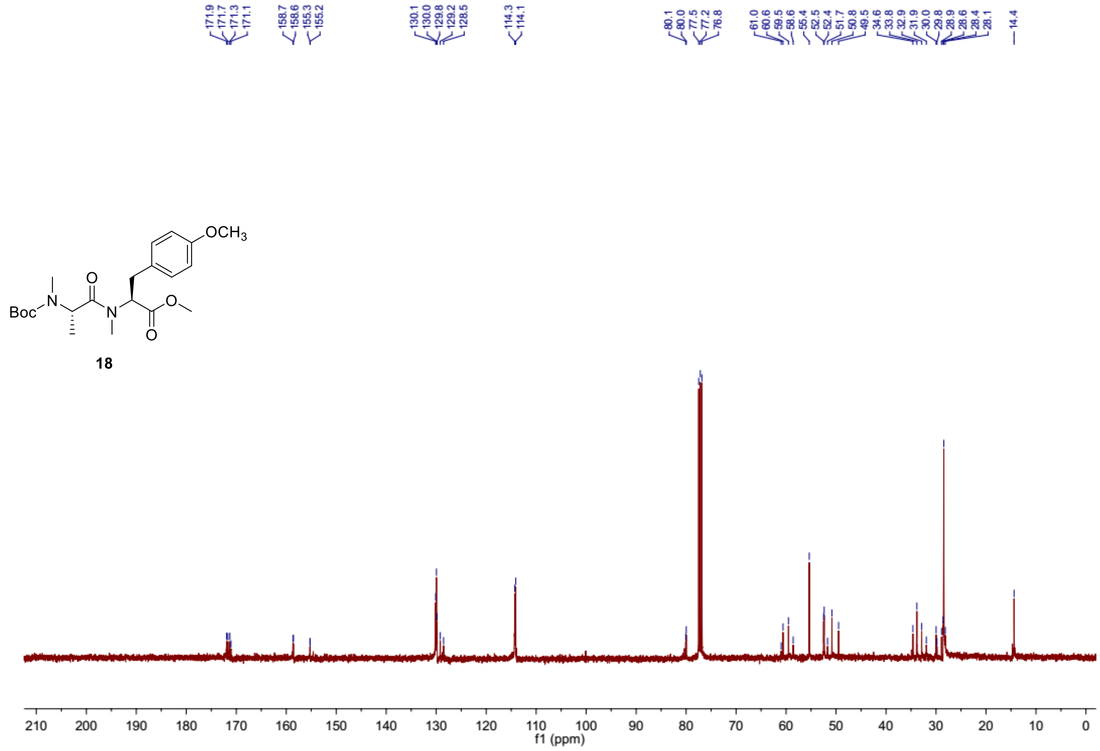

Figure S20. ^{13}C NMR (100 MHz, $CDCl_3$) spectrum of compound **18**.

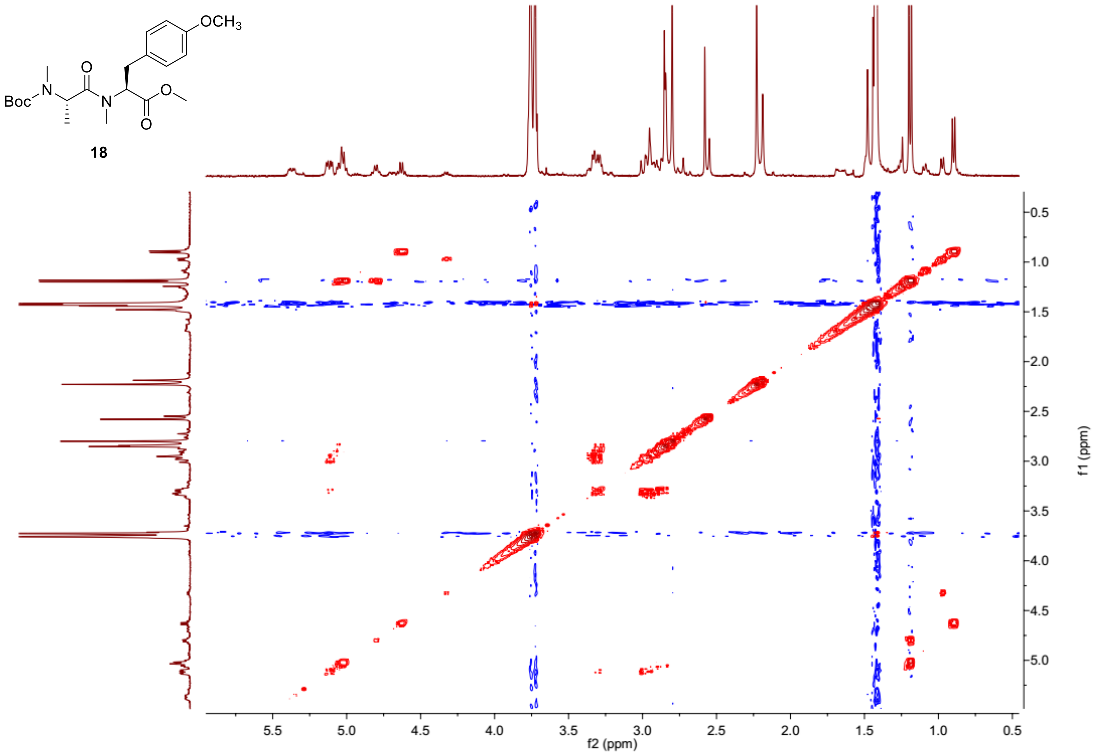

Figure S21. COSY (^{1}H, 400 MHz, $CDCl_3$) spectrum of compound **18**.

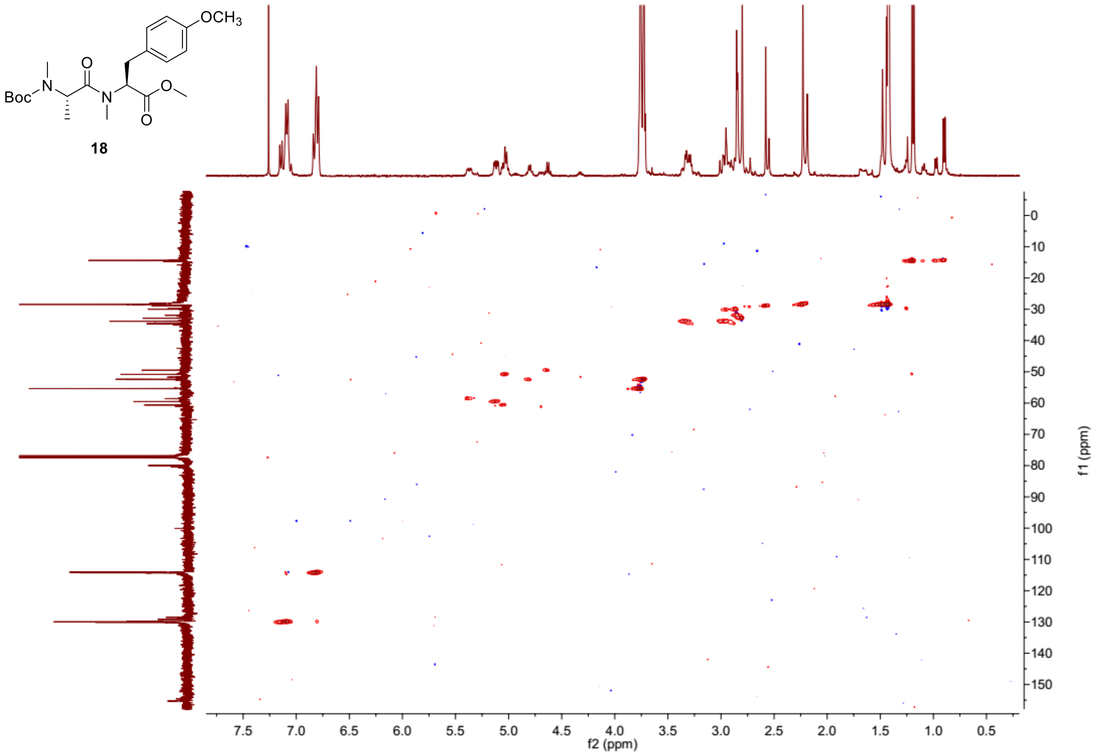

Figure S22. HSQC (^{1}H, 400 MHz, ^{13}C, 100 MHz, $CDCl_3$) spectrum of compound **18**.

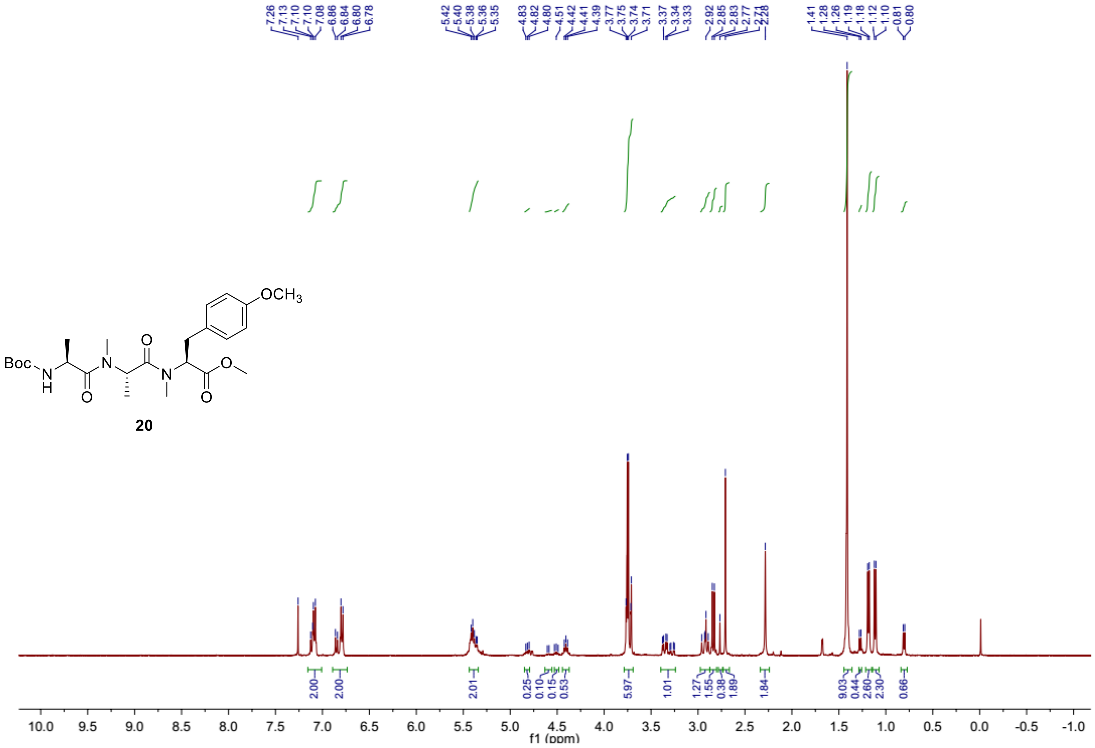

Figure S23. ^{1}H NMR (400 MHz, $CDCl_3$) spectrum of compound **20**.

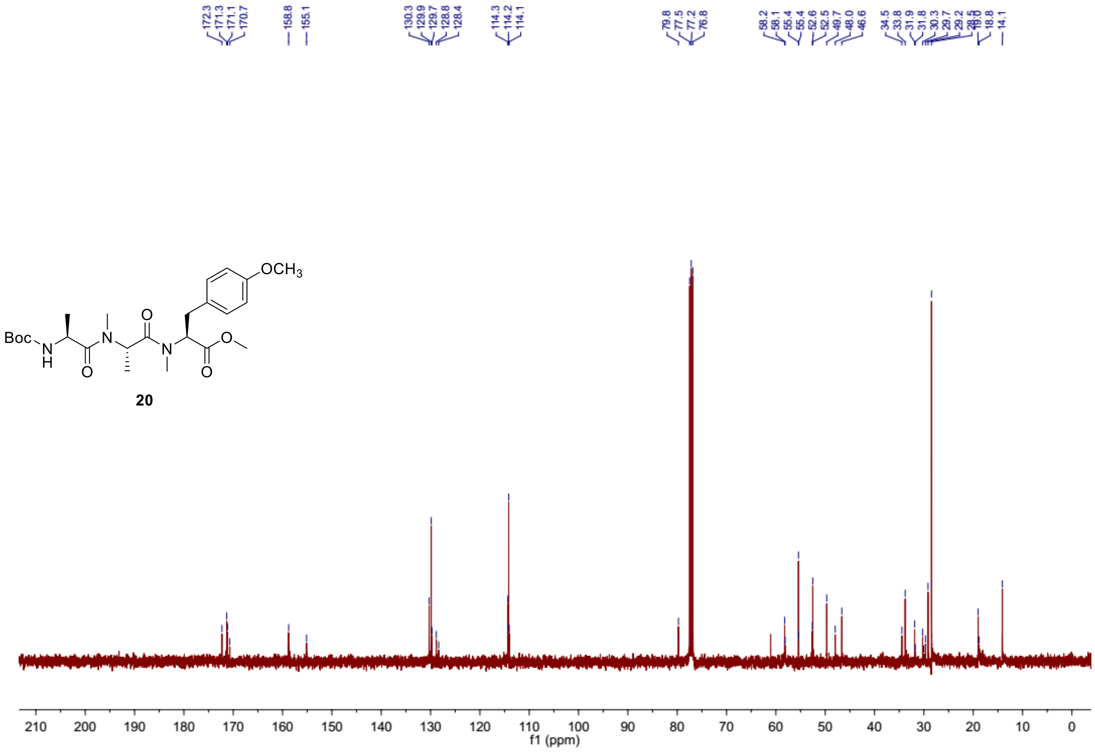

Figure S24. ^{13}C NMR (100 MHz, $CDCl_3$) spectrum of compound **20**.

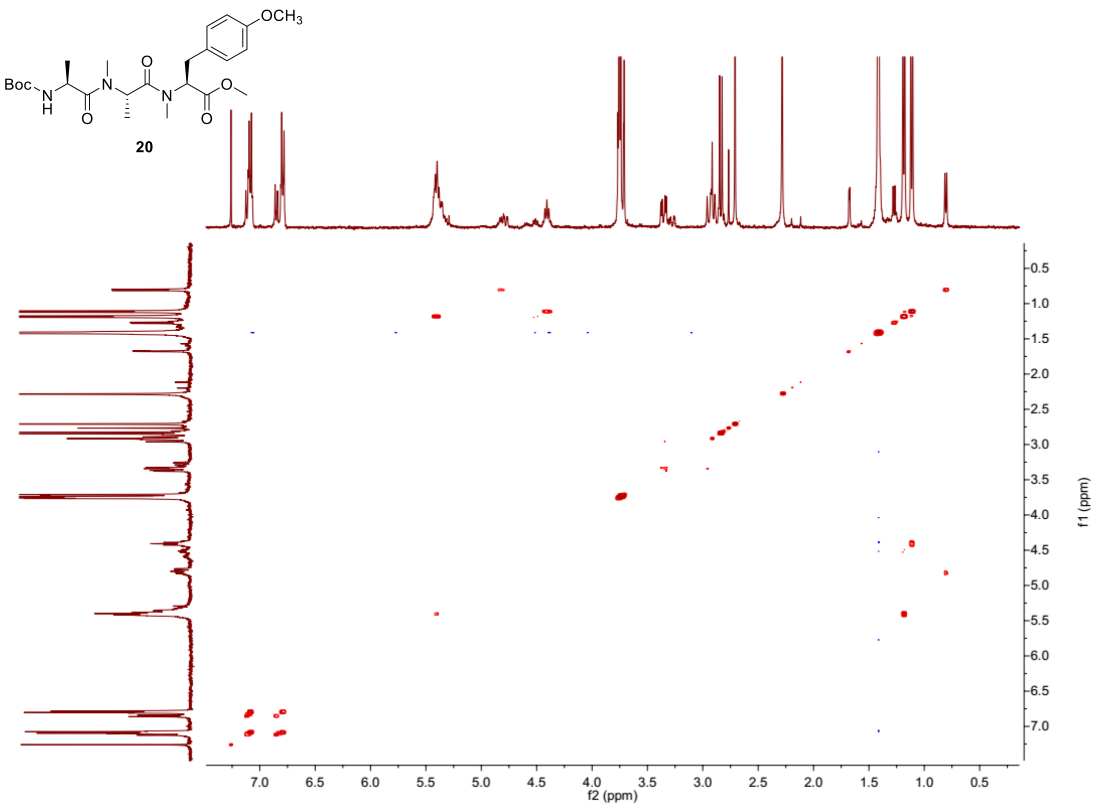

Figure S25. COSY (^{1}H, 400 MHz, $CDCl_3$) spectrum of compound **20**.

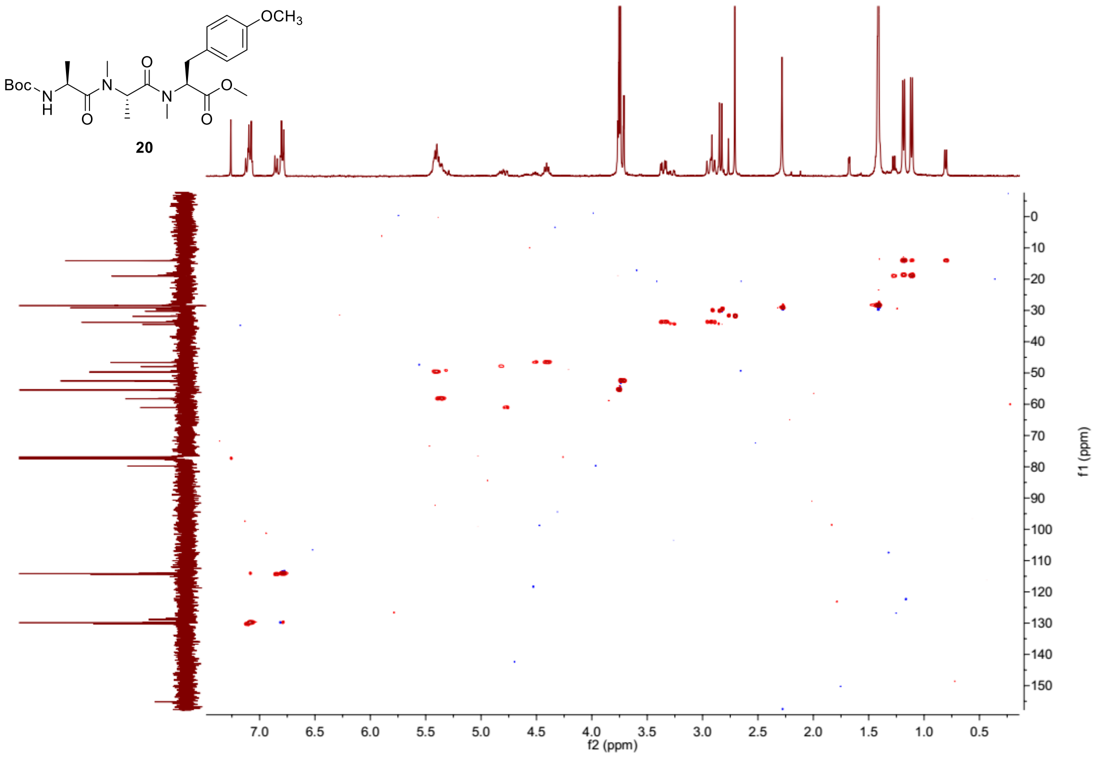

Figure S26. HSQC (^{1}H, 400 MHz, ^{13}C, 100 MHz, $CDCl_3$) spectrum of compound **20**.

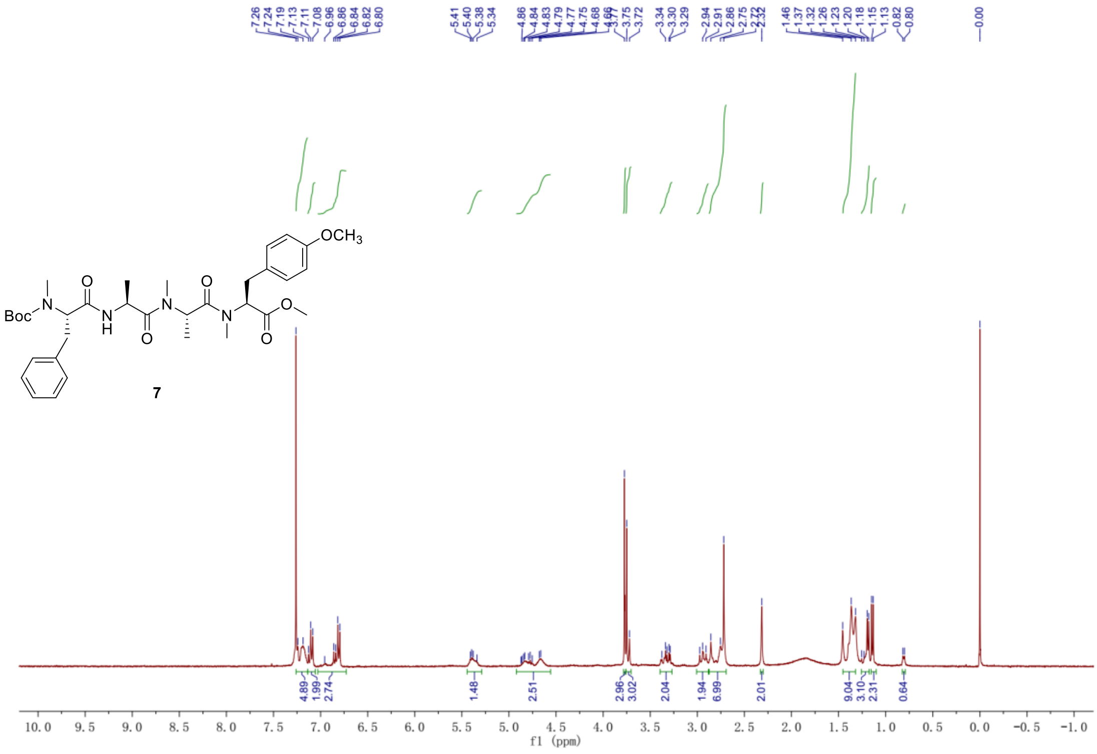

Figure S27. ^{1}H NMR (400 MHz, $CDCl_3$) spectrum of compound **7**.

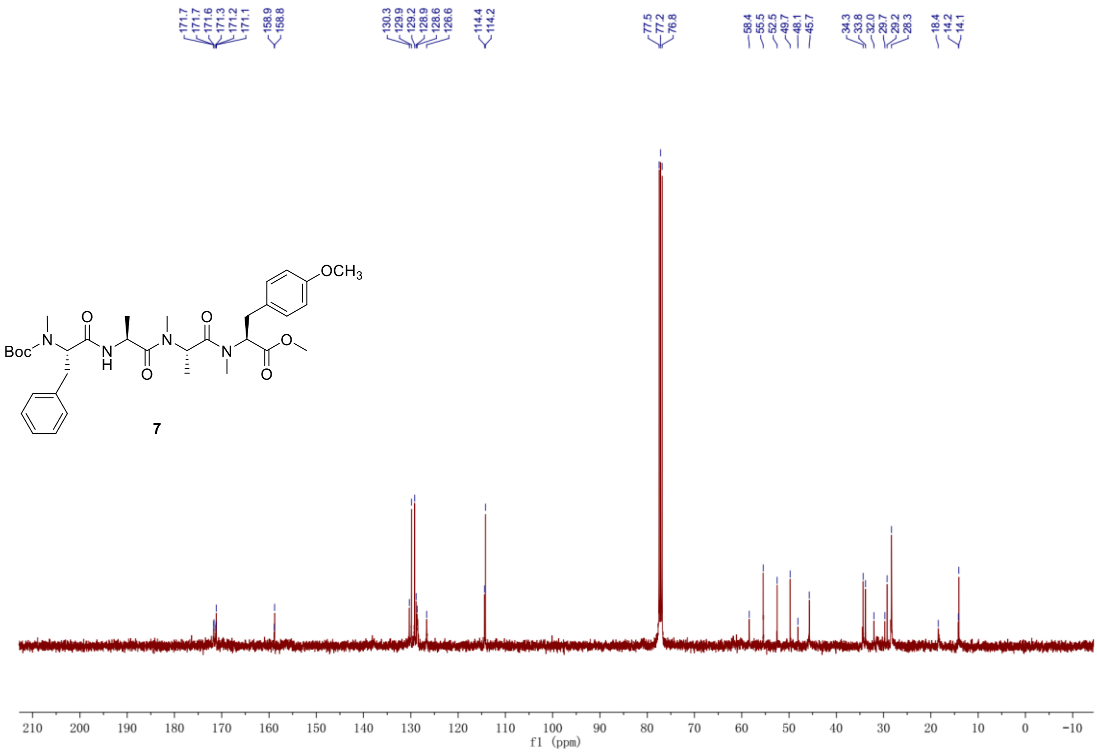

Figure S28. ^{13}C NMR (100 MHz, $CDCl_3$) spectrum of compound **7**.

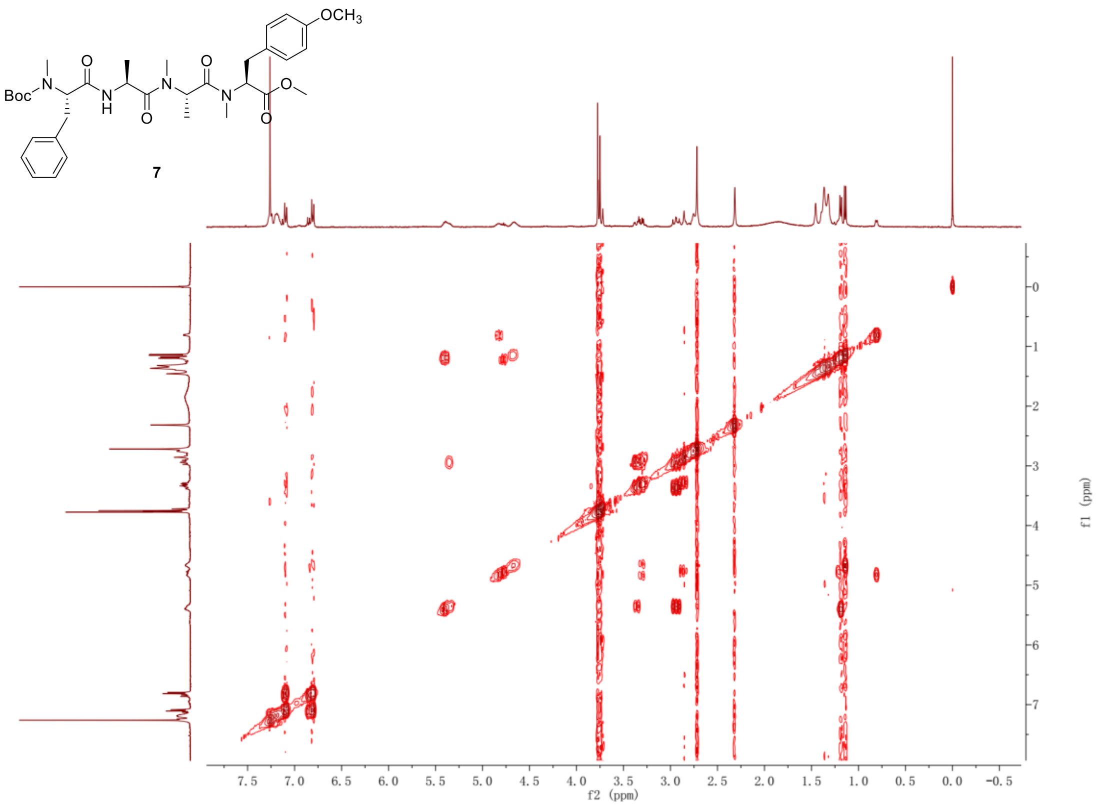

Figure S29. COSY (^{1}H, 400 MHz, $CDCl_3$) spectrum of compound **7**.

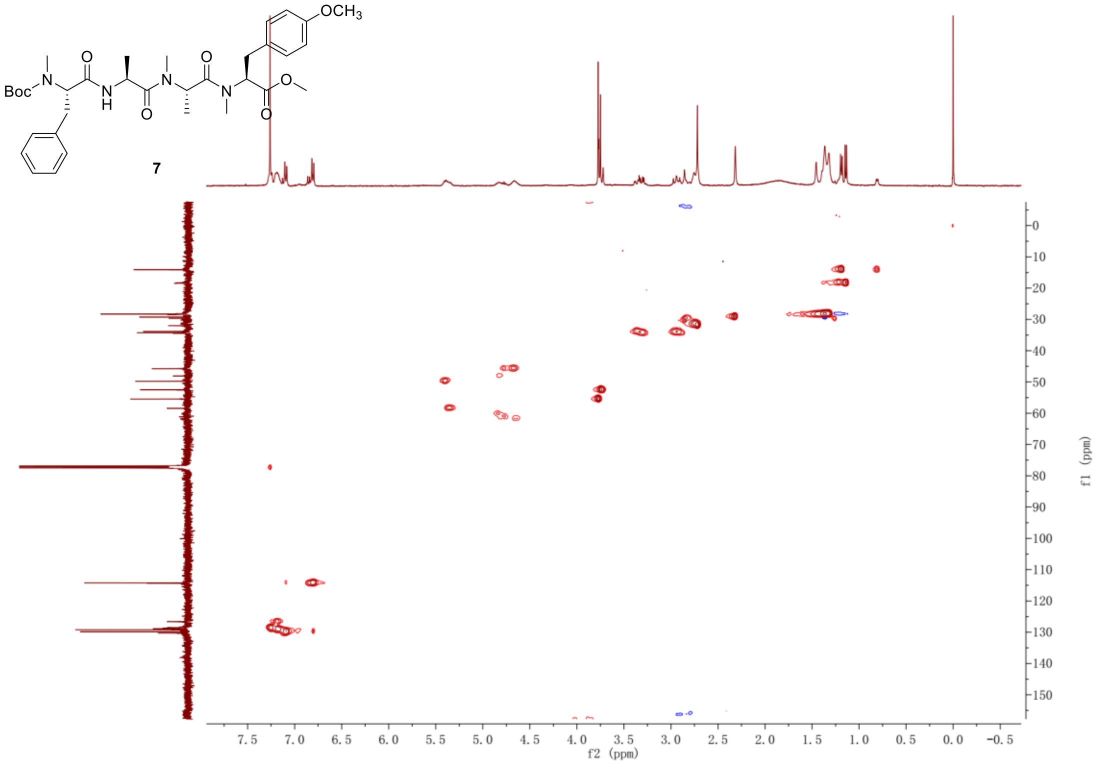

Figure S30. HSQC (^{1}H, 400 MHz, ^{13}C, 100 MHz, $CDCl_3$) spectrum of compound **7**.

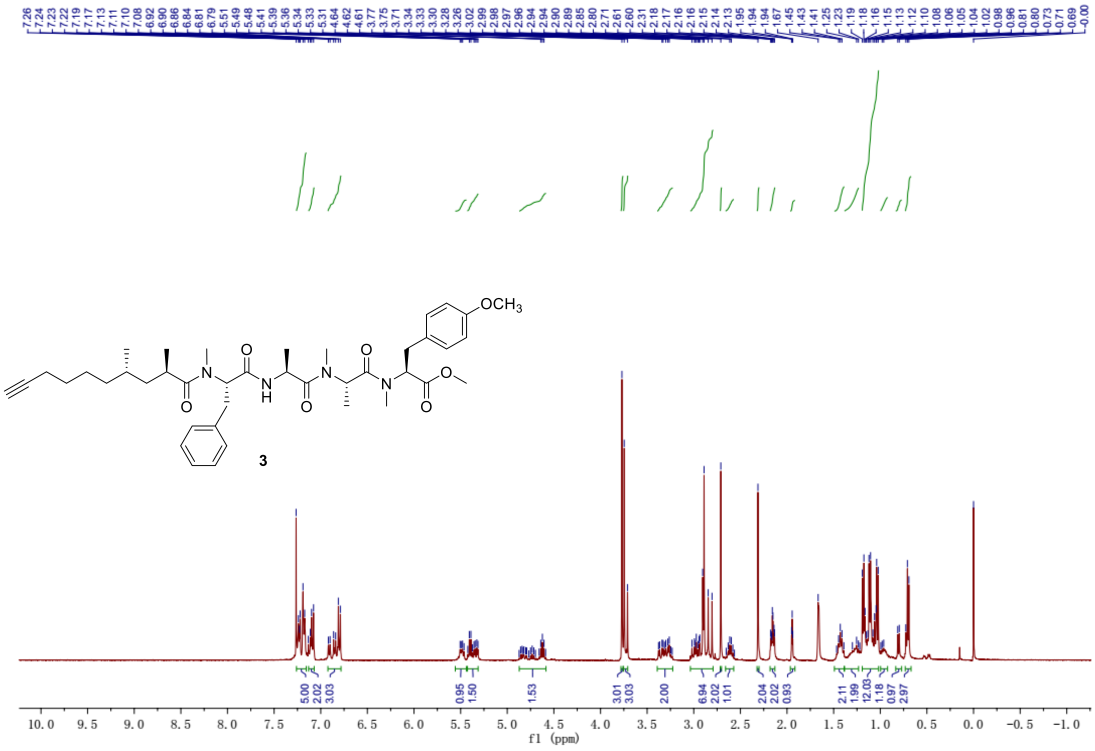

Figure S31. ^{1}H NMR (400 MHz, $CDCl_3$) spectrum of compound **3**.

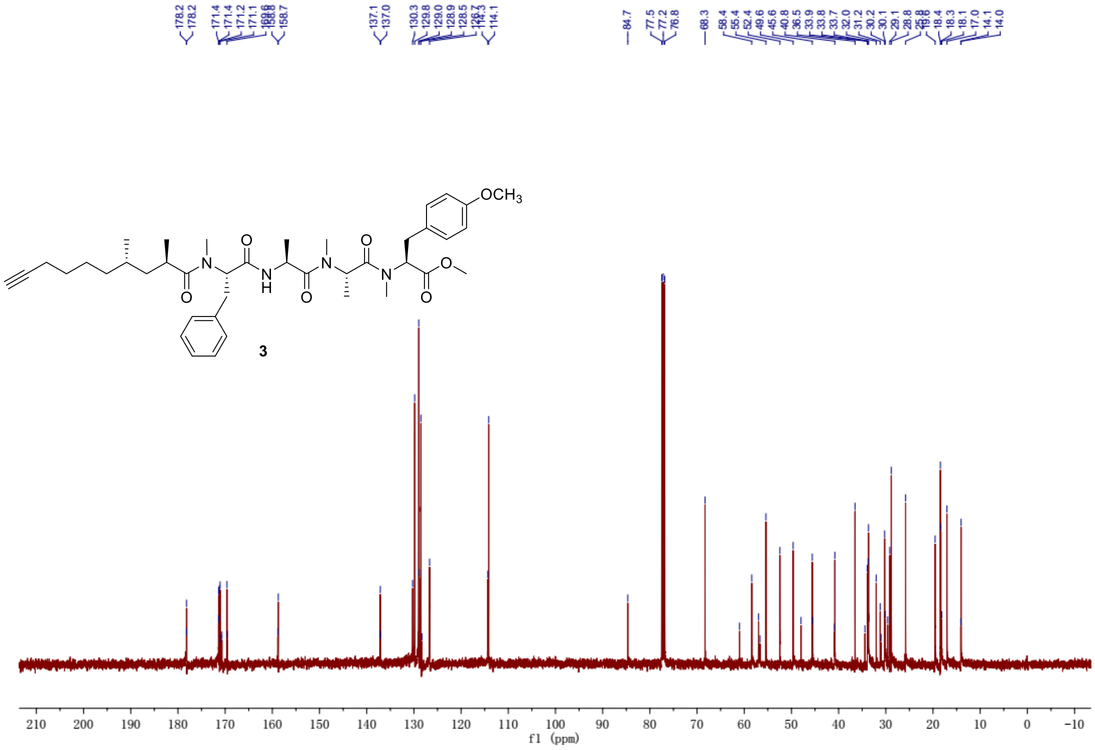

Figure S32. ^{13}C NMR (100 MHz, $CDCl_3$) spectrum of compound **3**.

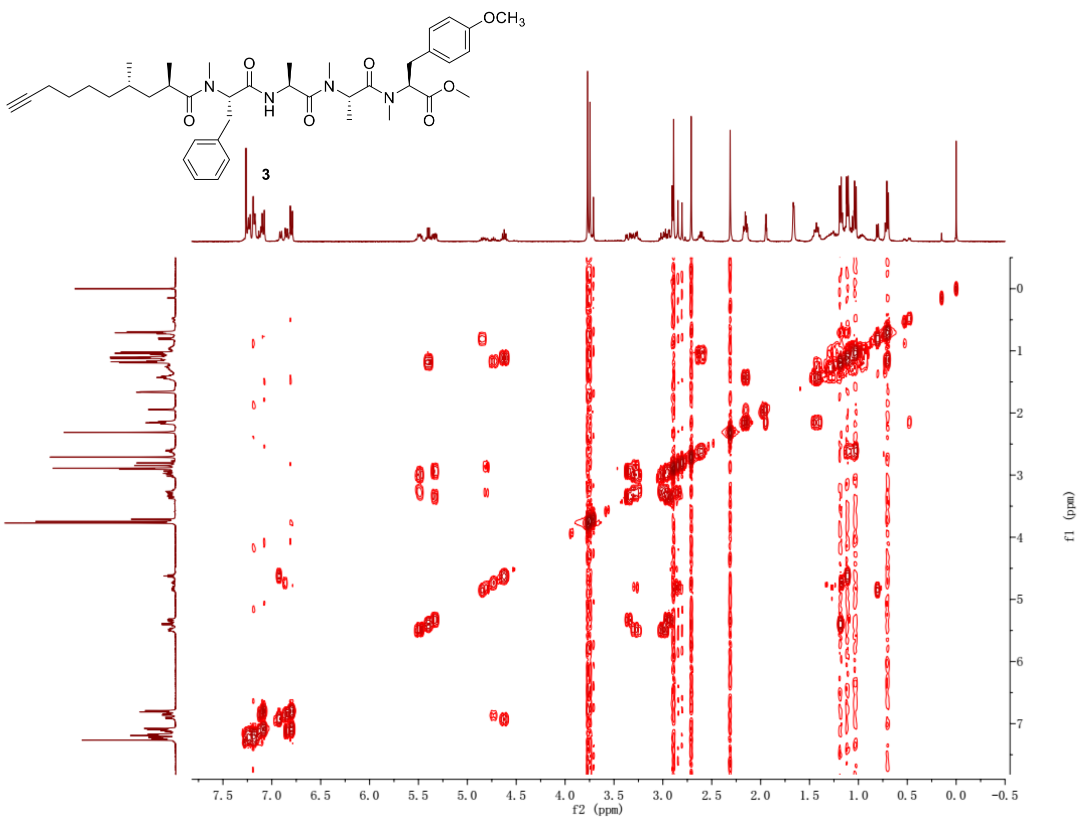

Figure S33. COSY (^{1}H, 400 MHz, $CDCl_3$) spectrum of compound **3**.

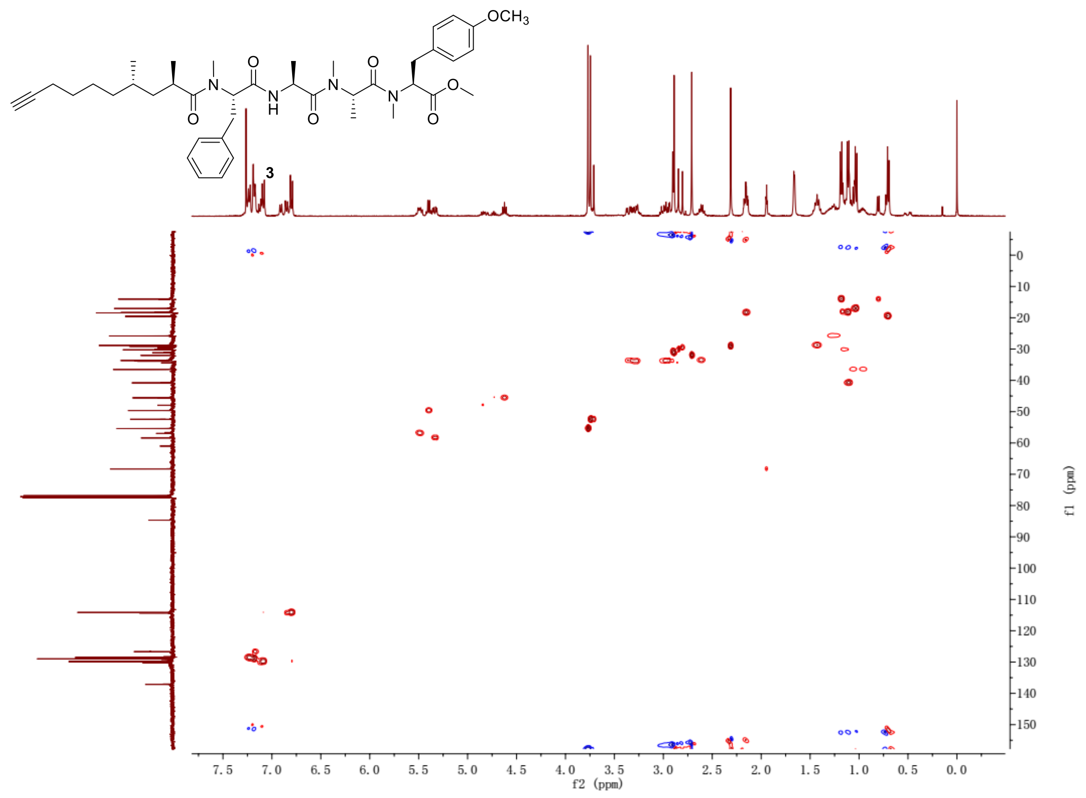

Figure S34. HSQC (^{1}H, 400 MHz, ^{13}C, 100 MHz, $CDCl_3$) spectrum of compound **3**.

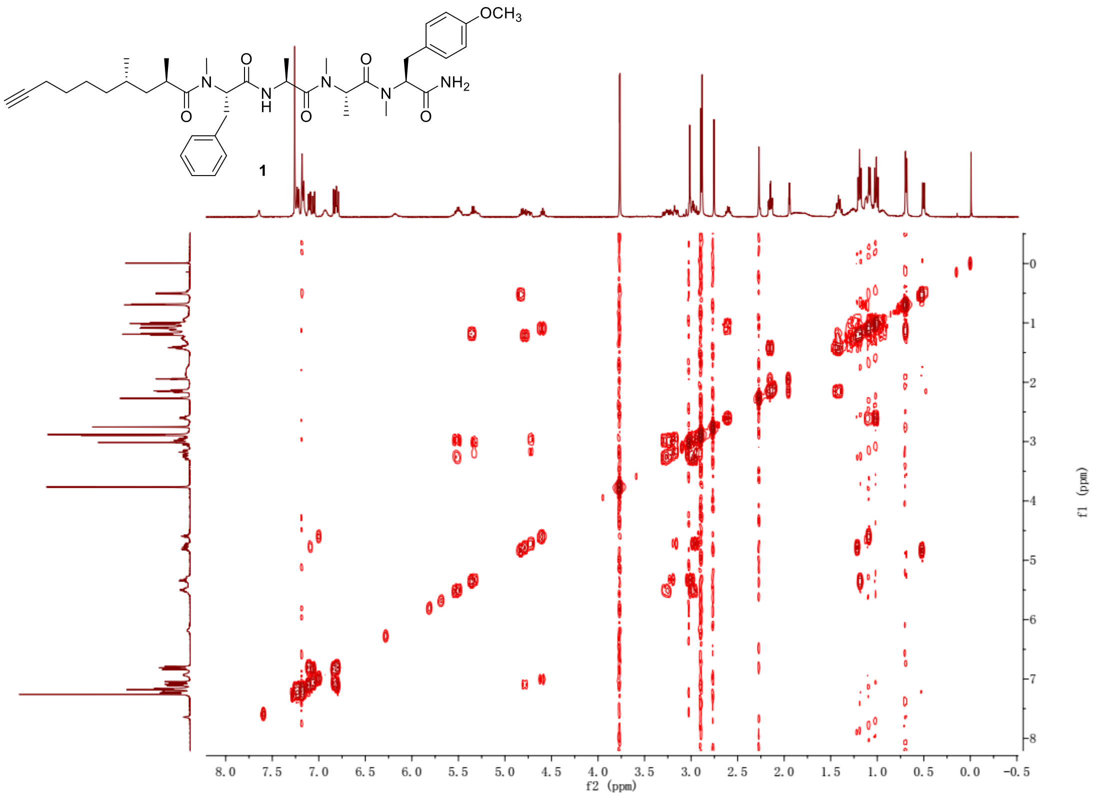

Figure S35. COSY (^{1}H, 400 MHz, $CDCl_3$) spectrum of synthetic **carmabin A (1)**.

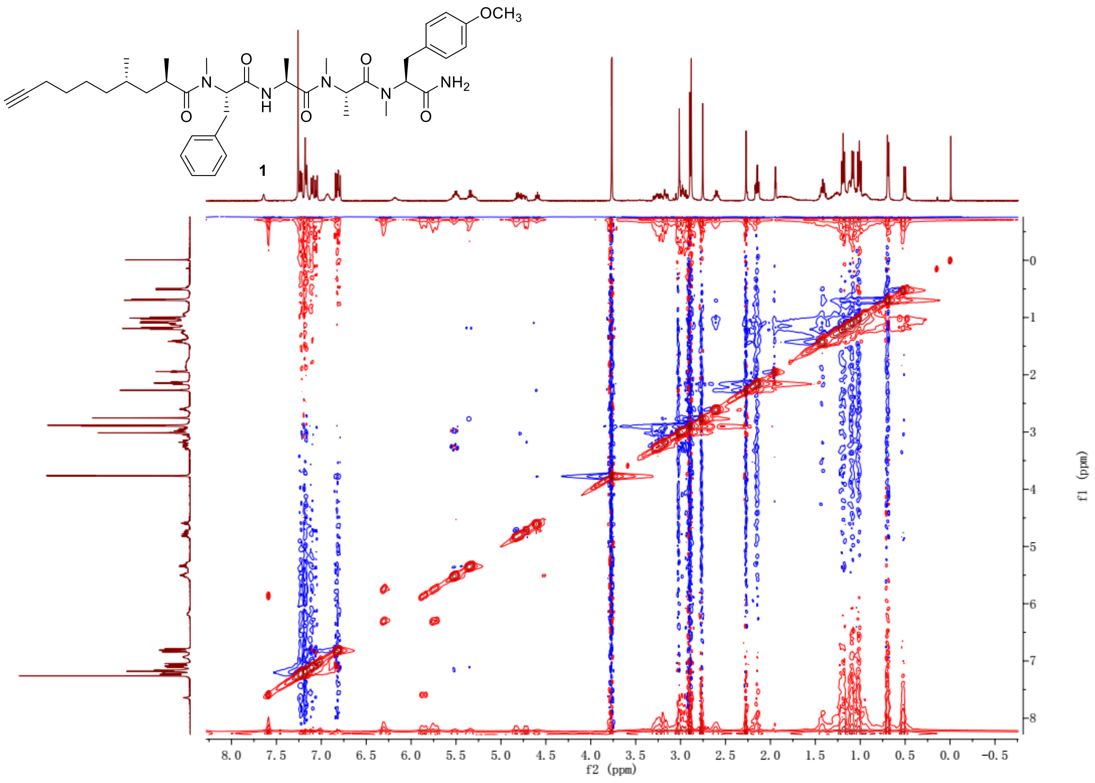

Figure S36. NOESY (^{1}H, 400 MHz, $CDCl_3$) spectrum of synthetic **carmabin A (1)**.

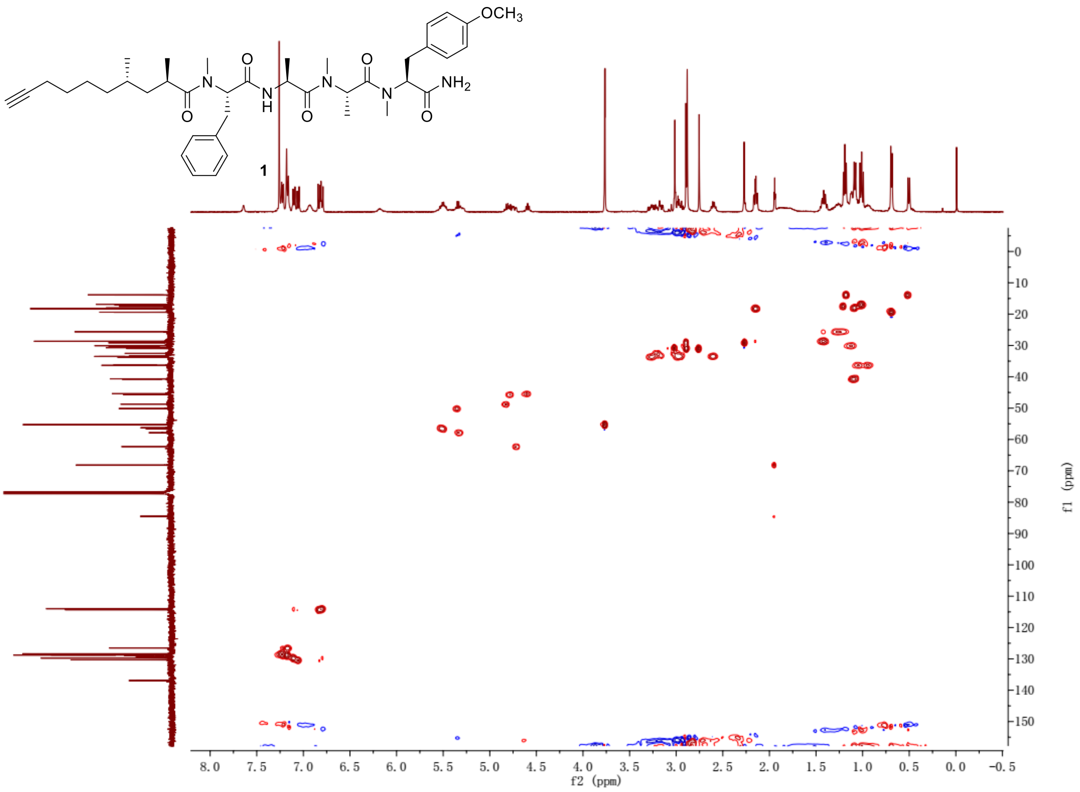

Figure S37. HSQC (^{1}H, 400 MHz, ^{13}C, 100 MHz, $CDCl_3$) spectrum of synthetic **carmabin A (1)**.

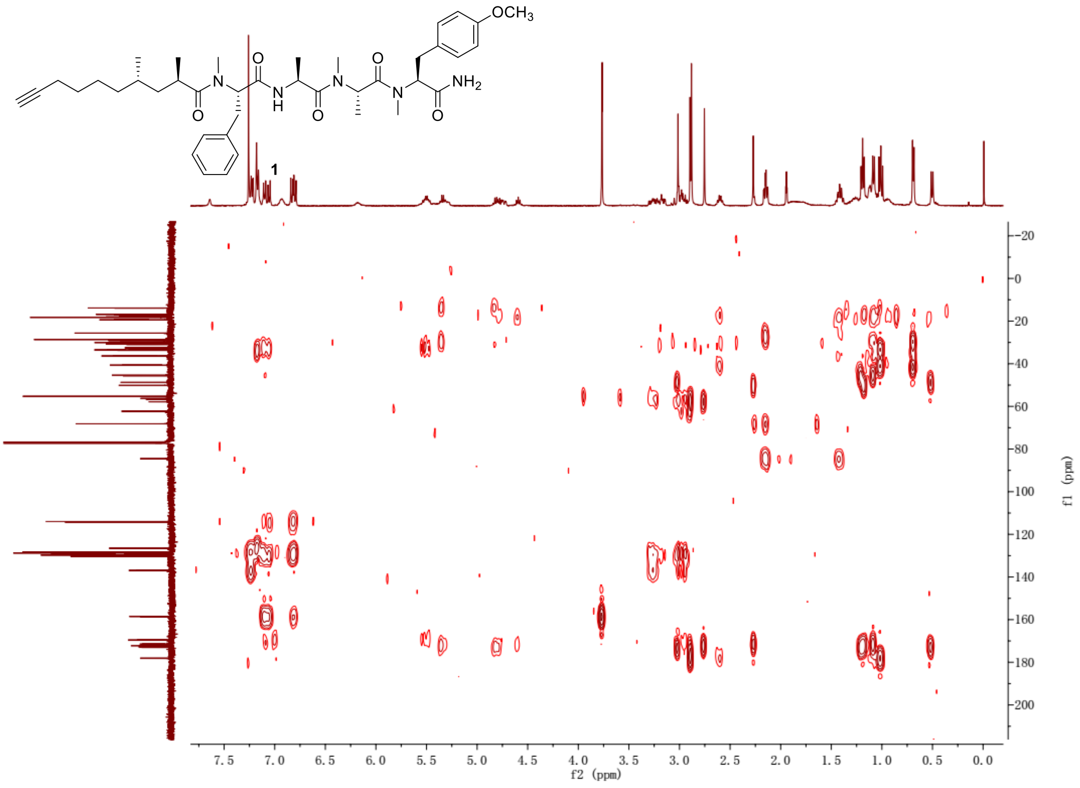

Figure S38. HMBC (^{1}H, 400 MHz, ^{13}C, 100 MHz, $CDCl_3$) spectrum of synthetic **carmabin A (1)**.

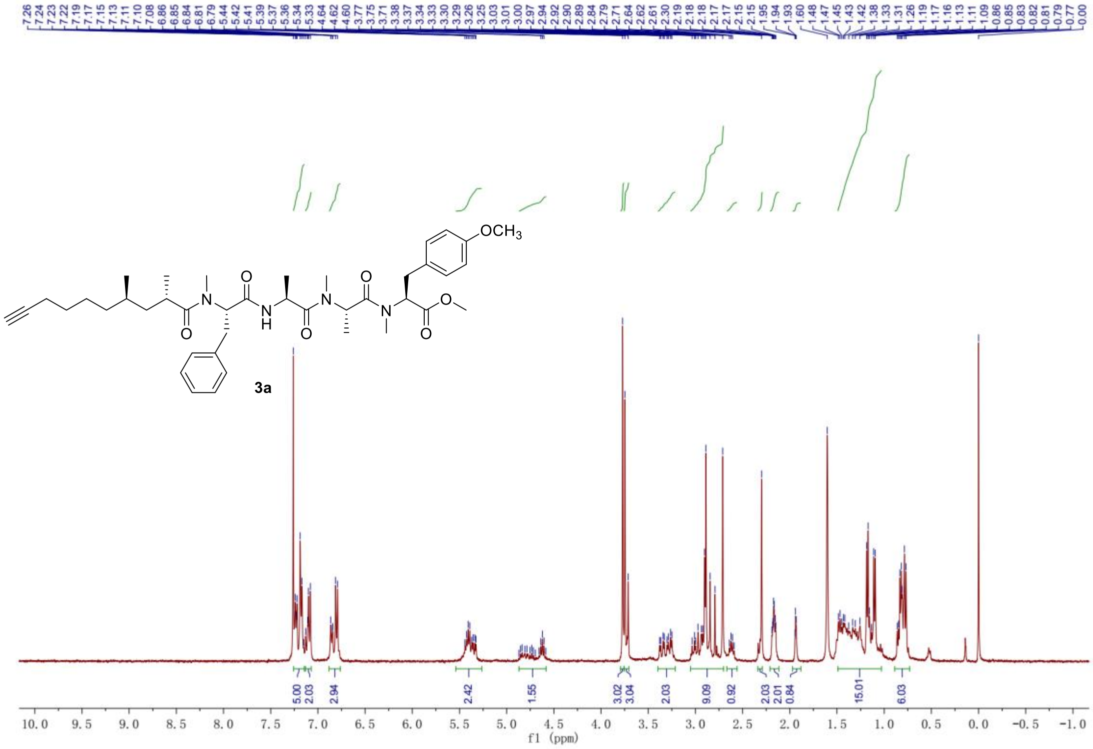

Figure S39. ^{1}H NMR (400 MHz, $CDCl_3$) spectrum of compound **3a**.

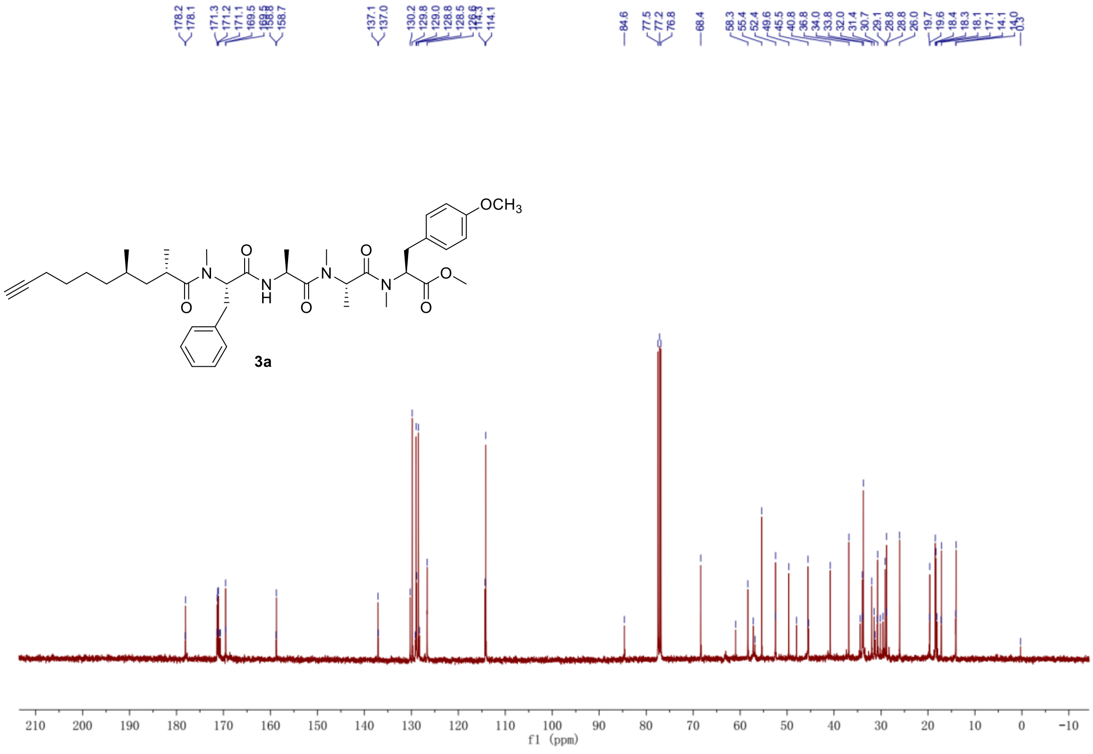

Figure S40. ^{13}C NMR (100 MHz, $CDCl_3$) spectrum of compound **3a**.

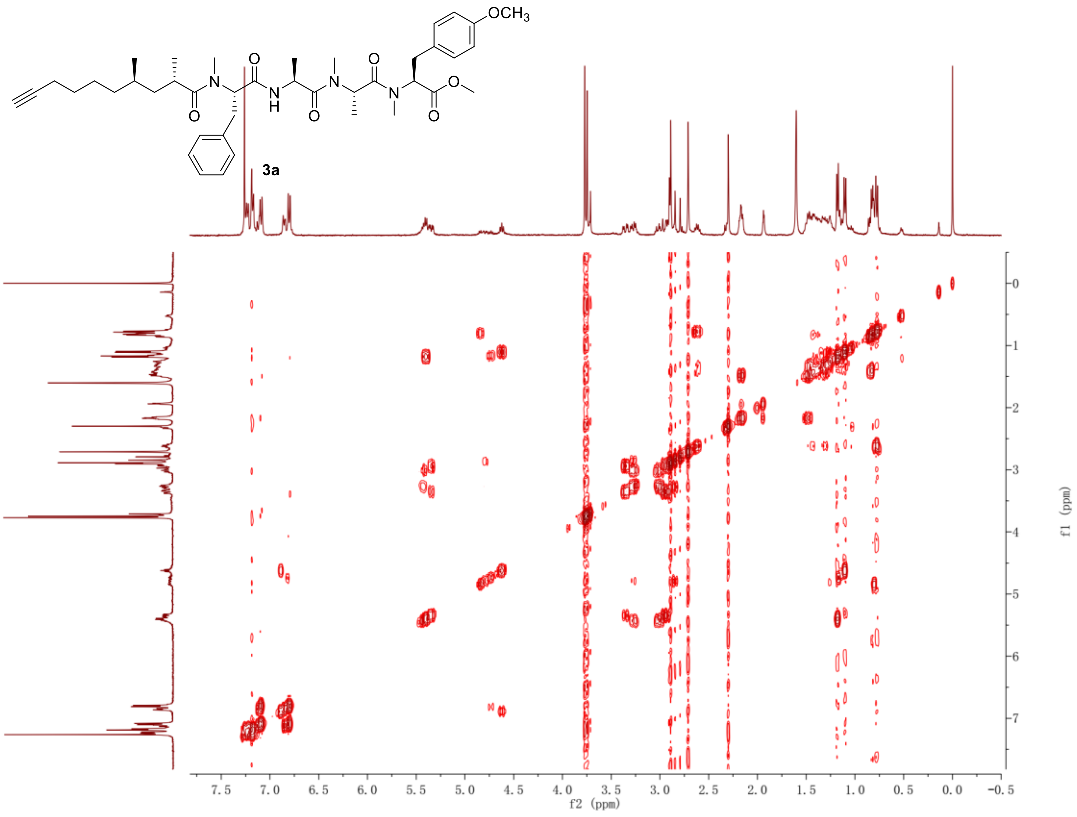

Figure S41. COSY (^{1}H, 400 MHz, $CDCl_3$) spectrum of compound **3a**.

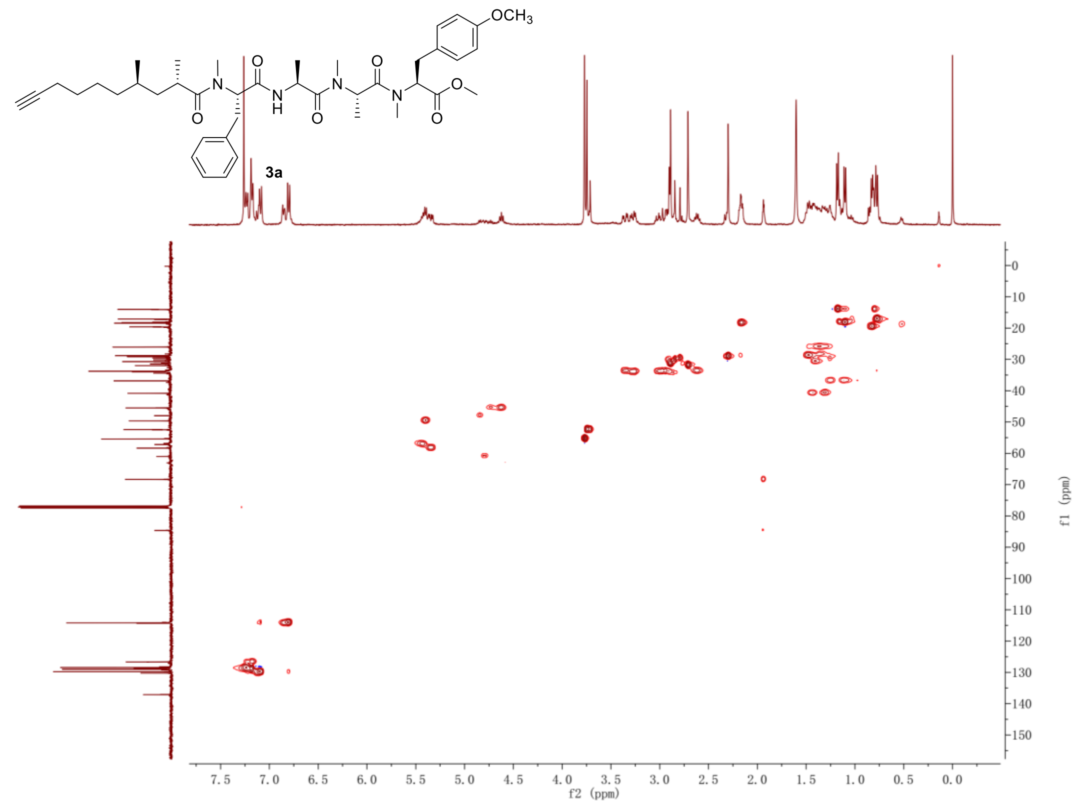

Figure S42. HSQC (^{1}H, 400 MHz, ^{13}C, 100 MHz, $CDCl_3$) spectrum of compound **3a**.

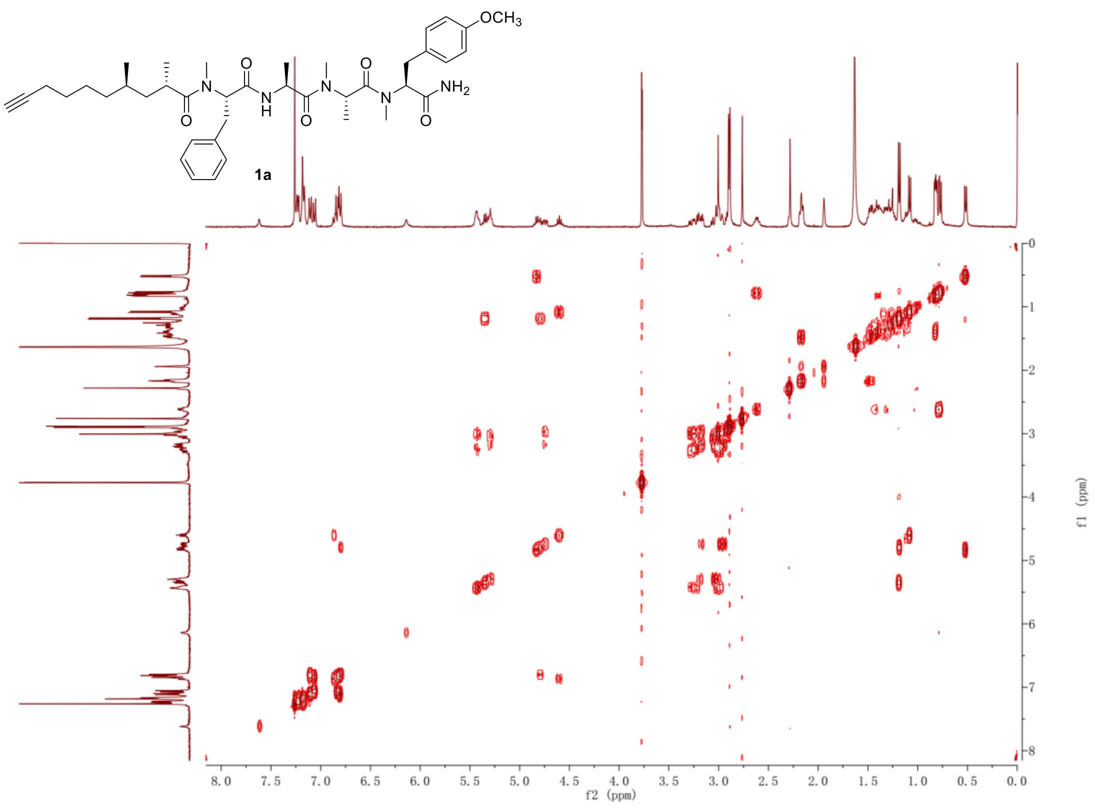

Figure S43. COSY (^{1}H, 400 MHz, $CDCl_3$) spectrum of compound **1a**.

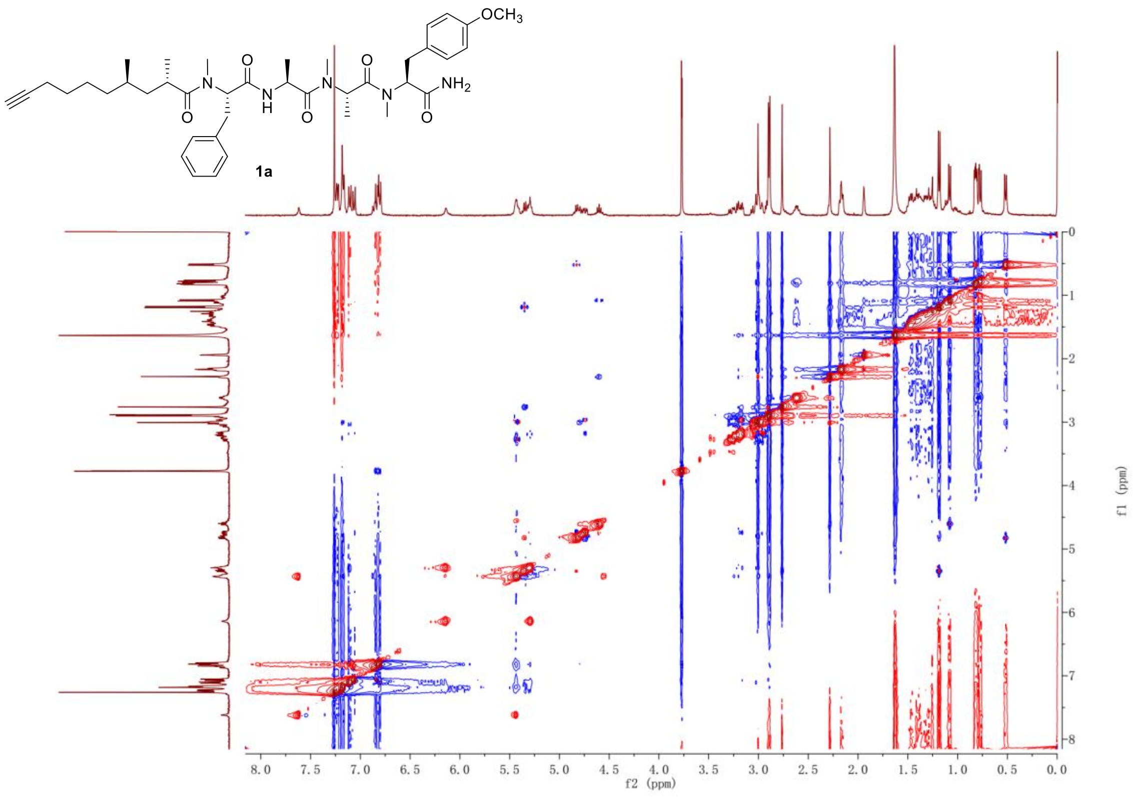

Figure S44. NOESY (^{1}H, 400 MHz, $CDCl_3$) spectrum of compound **1a**.

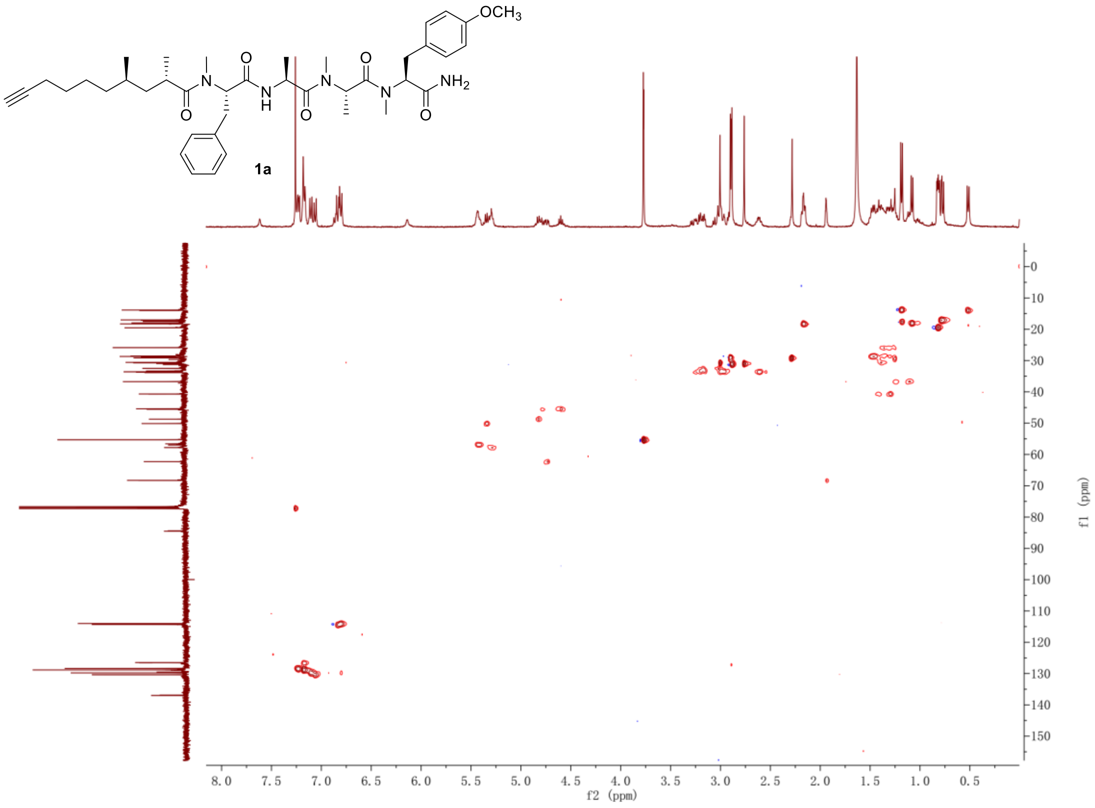

Figure S45. HSQC (^{1}H, 400 MHz, ^{13}C, 100 MHz, $CDCl_3$) spectrum of compound **1a**.

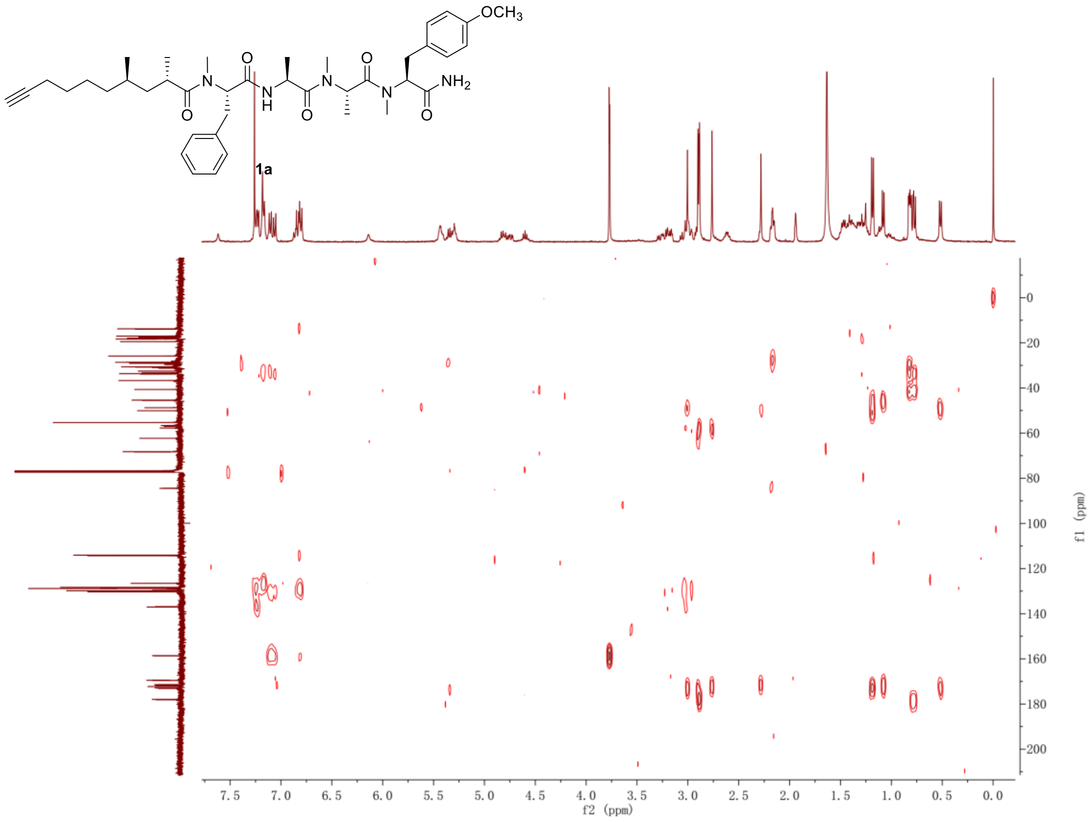

Figure S46. HMBC (^{1}H, 400 MHz, ^{13}C, 100 MHz, $CDCl_3$) spectrum of compound **1a**.

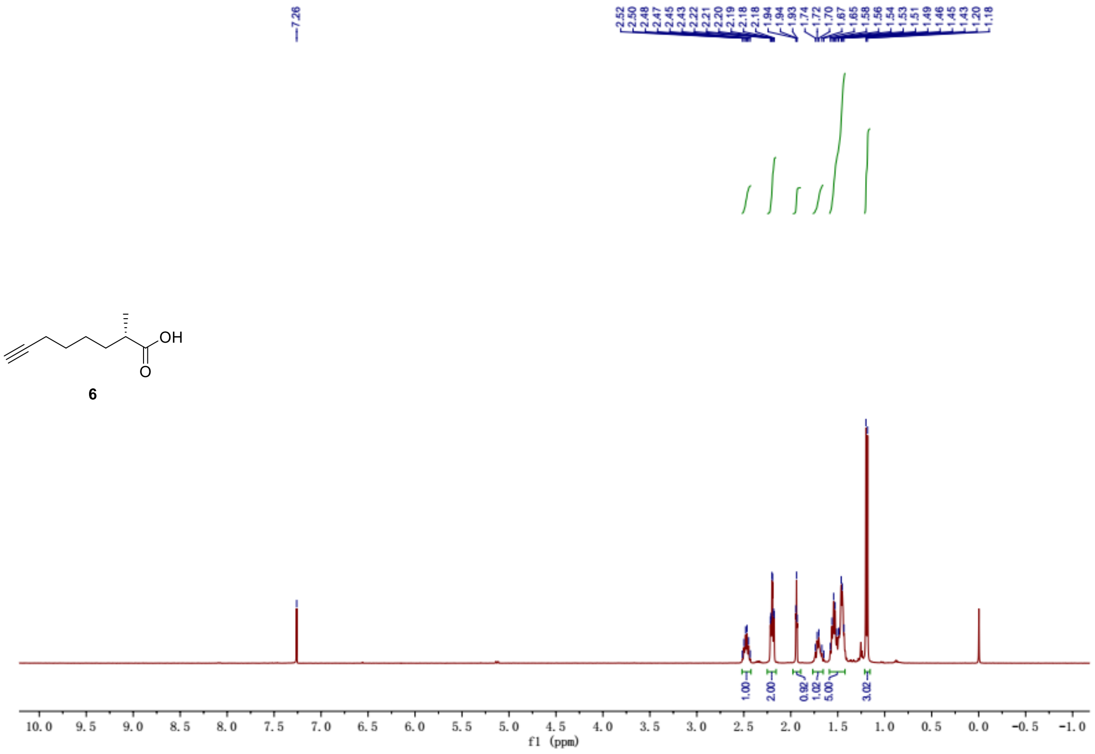

Figure S47. ^{1}H NMR (400 MHz, $CDCl_3$) spectrum of compound **6**.

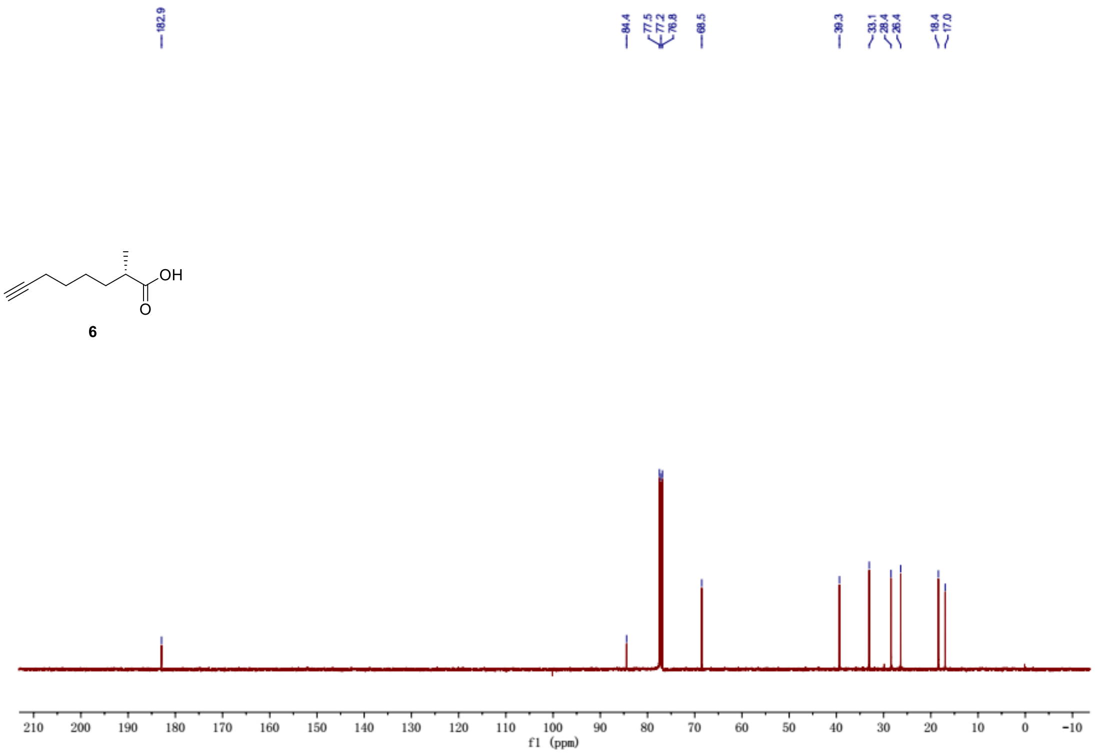

Figure S48. ^{13}C NMR (100 MHz, $CDCl_3$) spectrum of compound **6**.

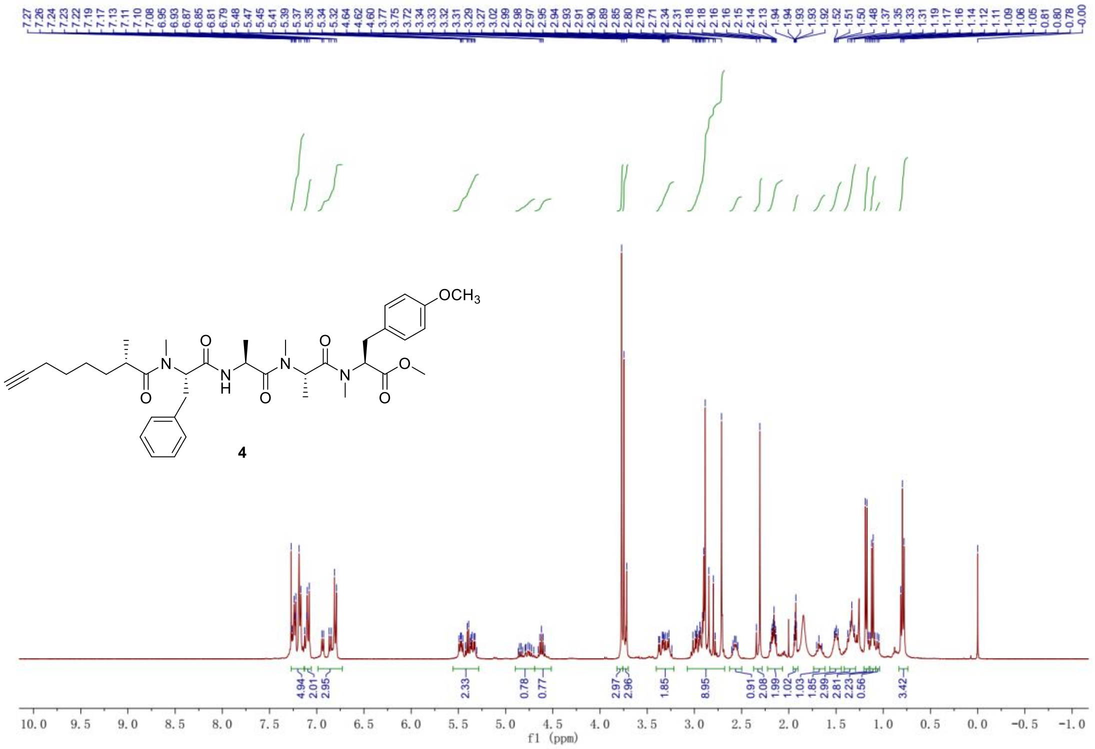

Figure S49. ^{1}H NMR (400 MHz, $CDCl_3$) spectrum of compound **4**.

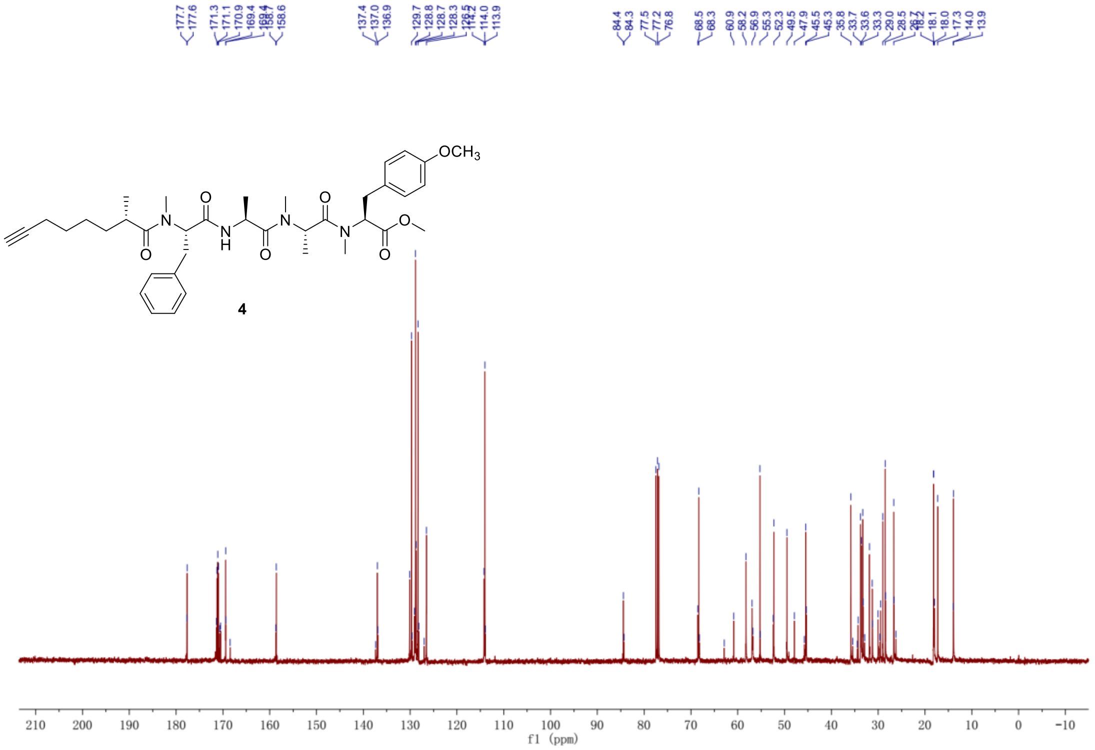

Figure S50. ^{13}C NMR (100 MHz, $CDCl_3$) spectrum of compound **4**.

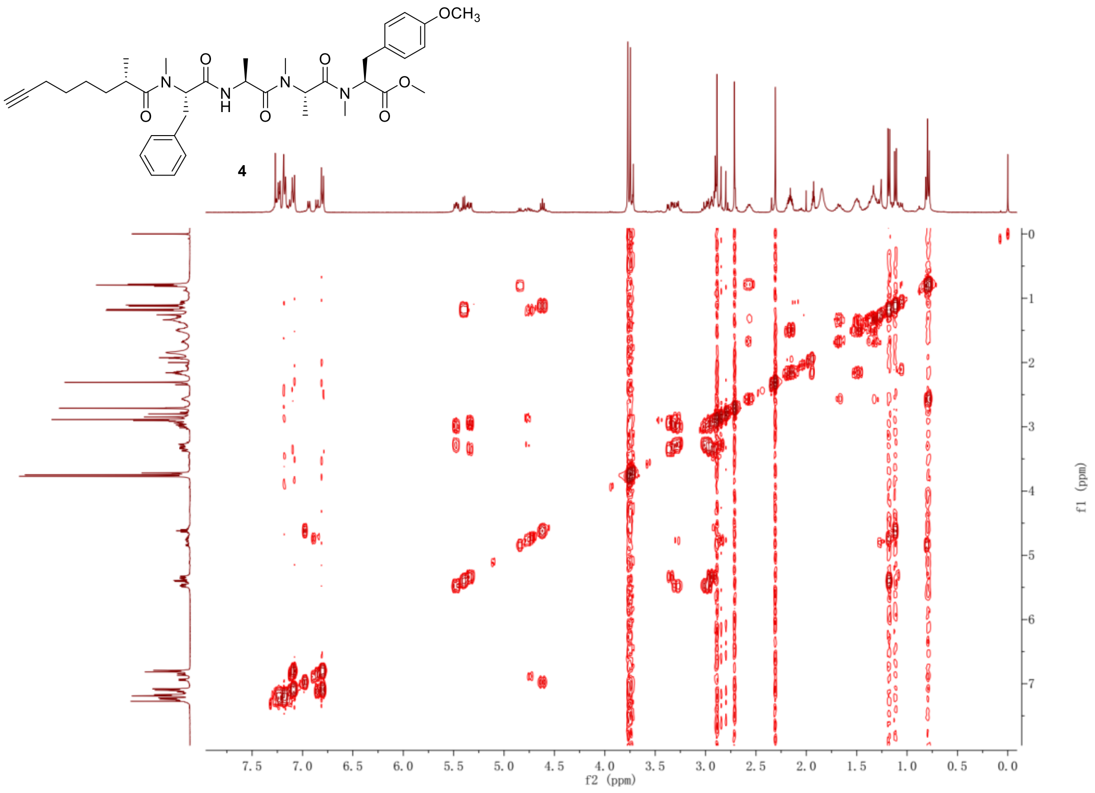

Figure S51. COSY (^1H, 400 MHz, $CDCl_3$) spectrum of compound **4**.

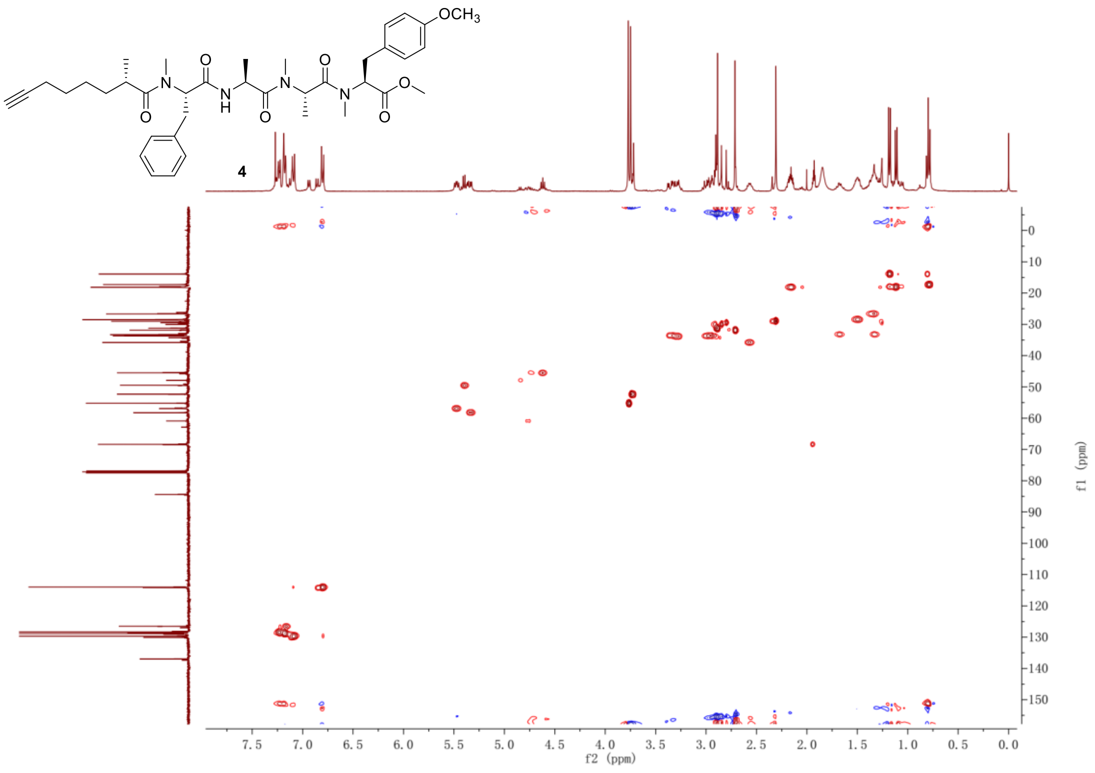

Figure S52. HSQC (^1H, 400 MHz, ^{13}C, 100 MHz, $CDCl_3$) spectrum of compound **4**.

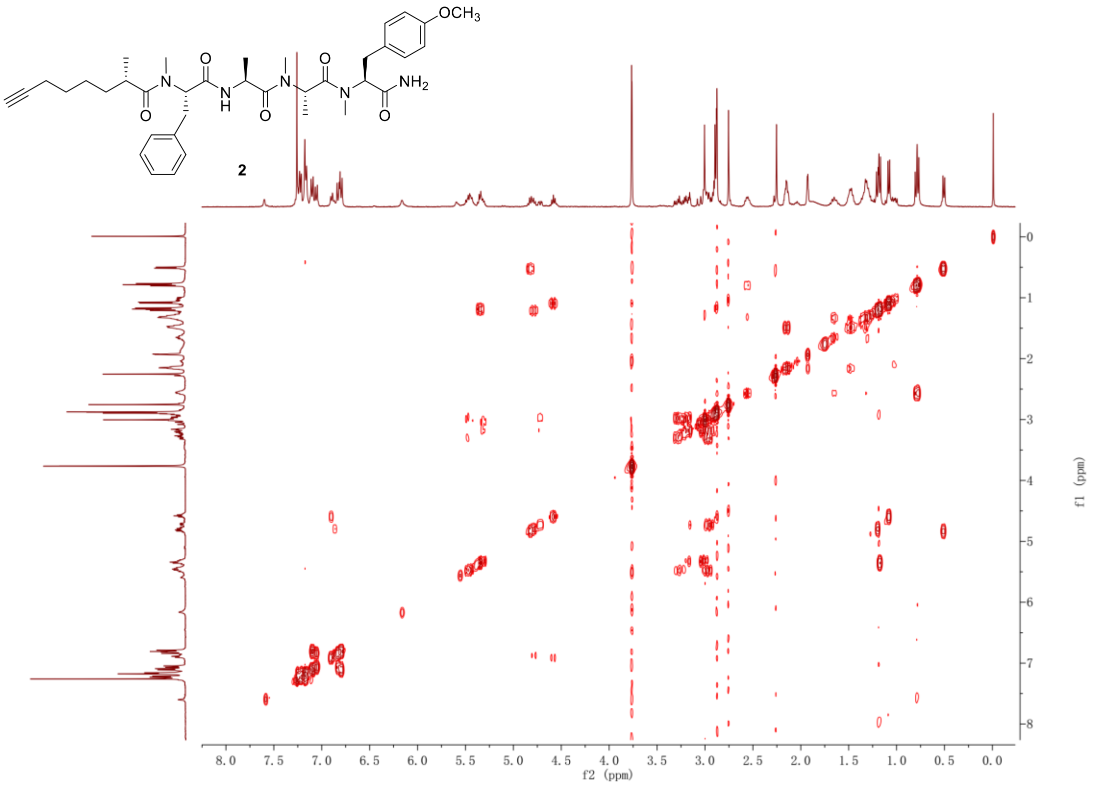

Figure S53. COSY (^1H, 400 MHz, $CDCl_3$) spectrum of compound **2**.

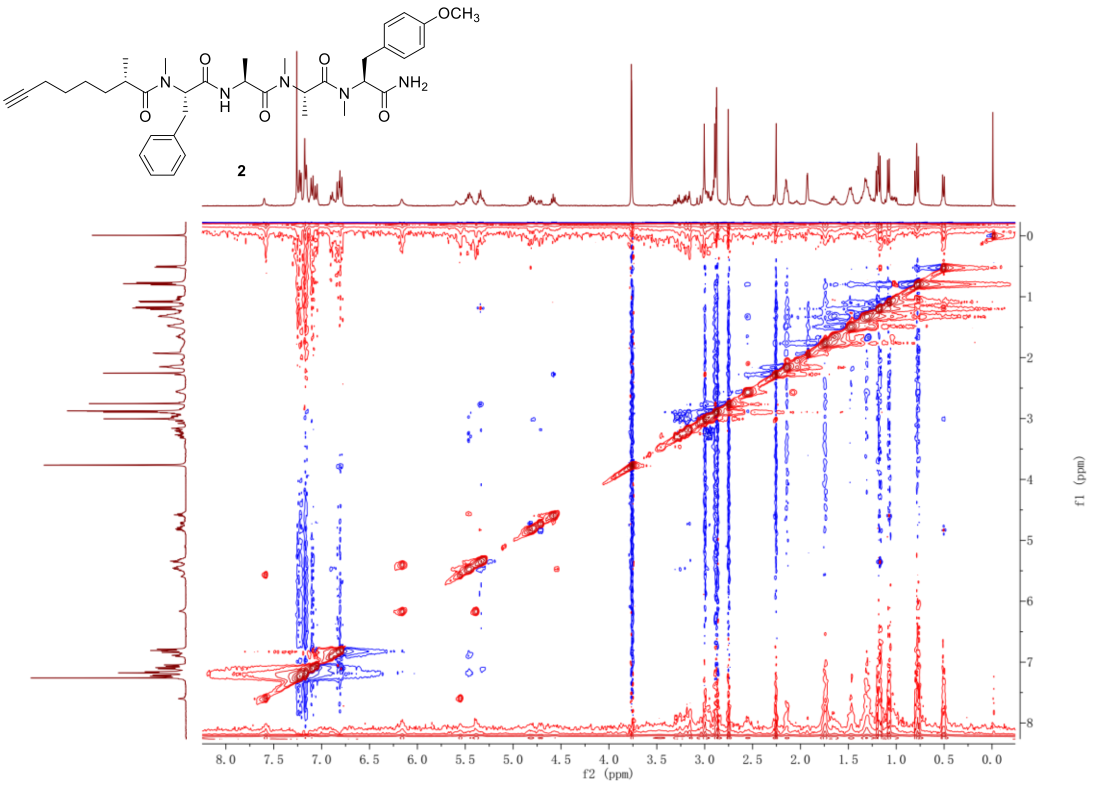

Figure S54. NOESY (^1H, 400 MHz, $CDCl_3$) spectrum of compound **2**.

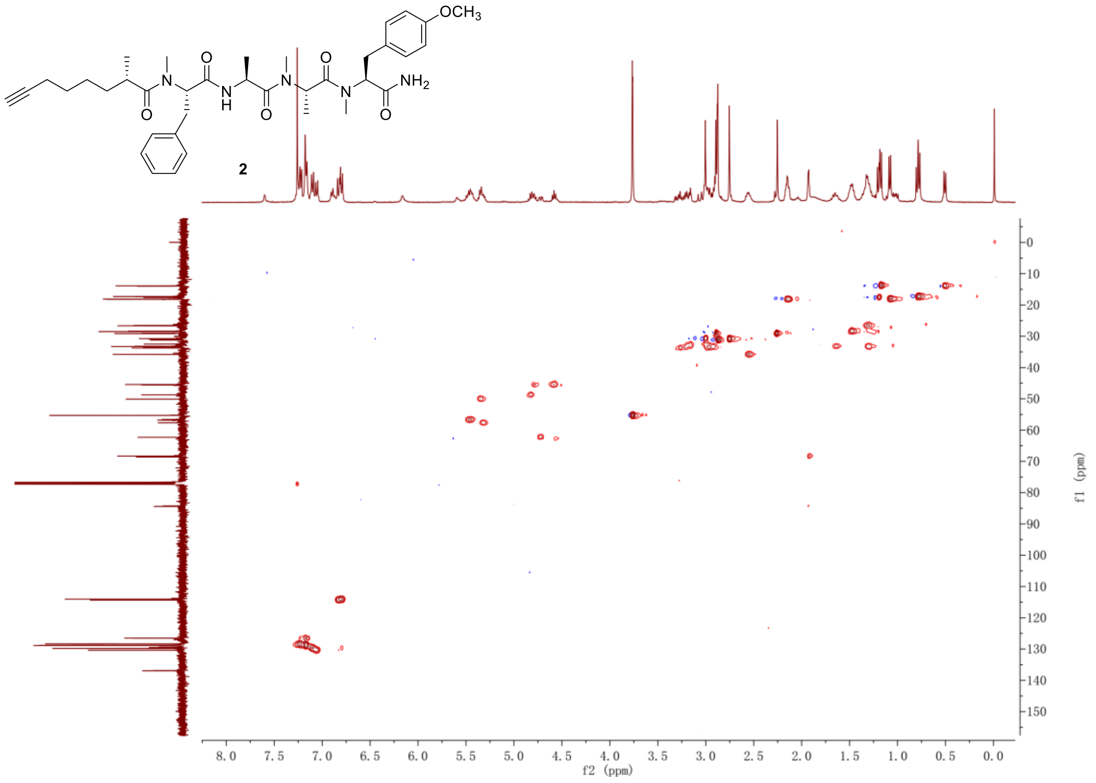

Figure S55. HSQC (^{1}H, 400 MHz, ^{13}C, 100 MHz, $CDCl_3$) spectrum of compound **2**.

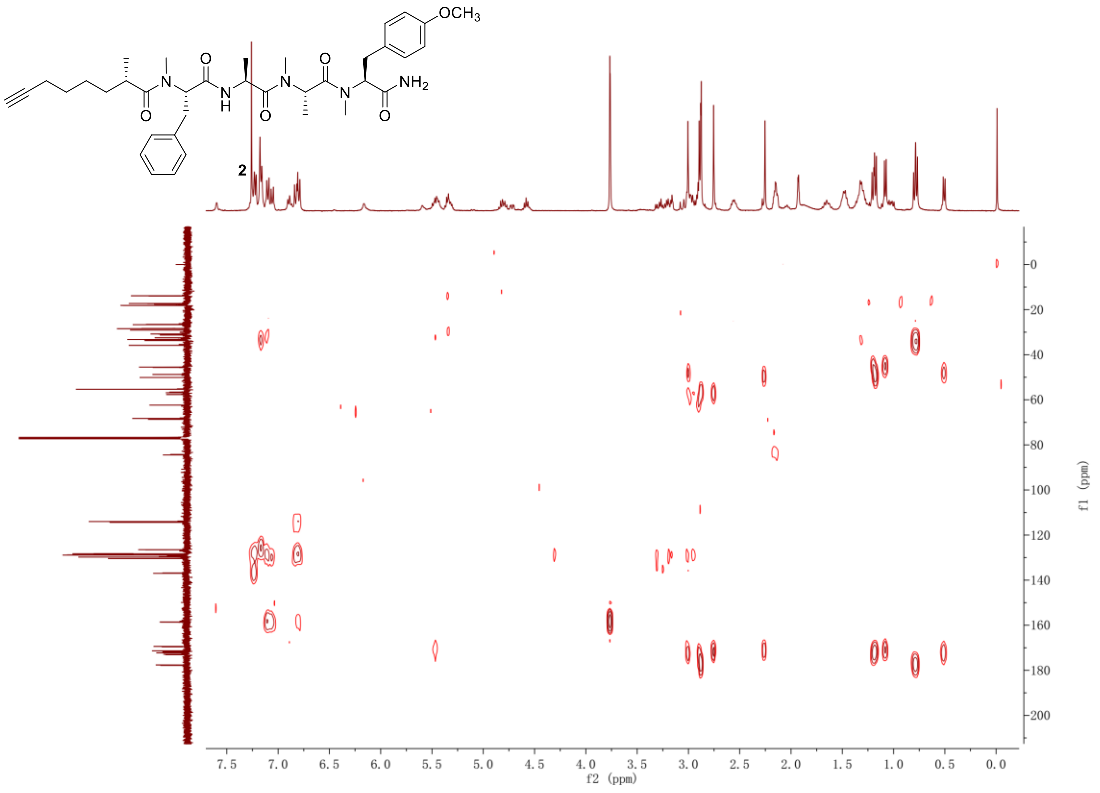

Figure S56. HMBC (^{1}H, 400 MHz, ^{13}C, 100 MHz, $CDCl_3$) spectrum of compound **2**.

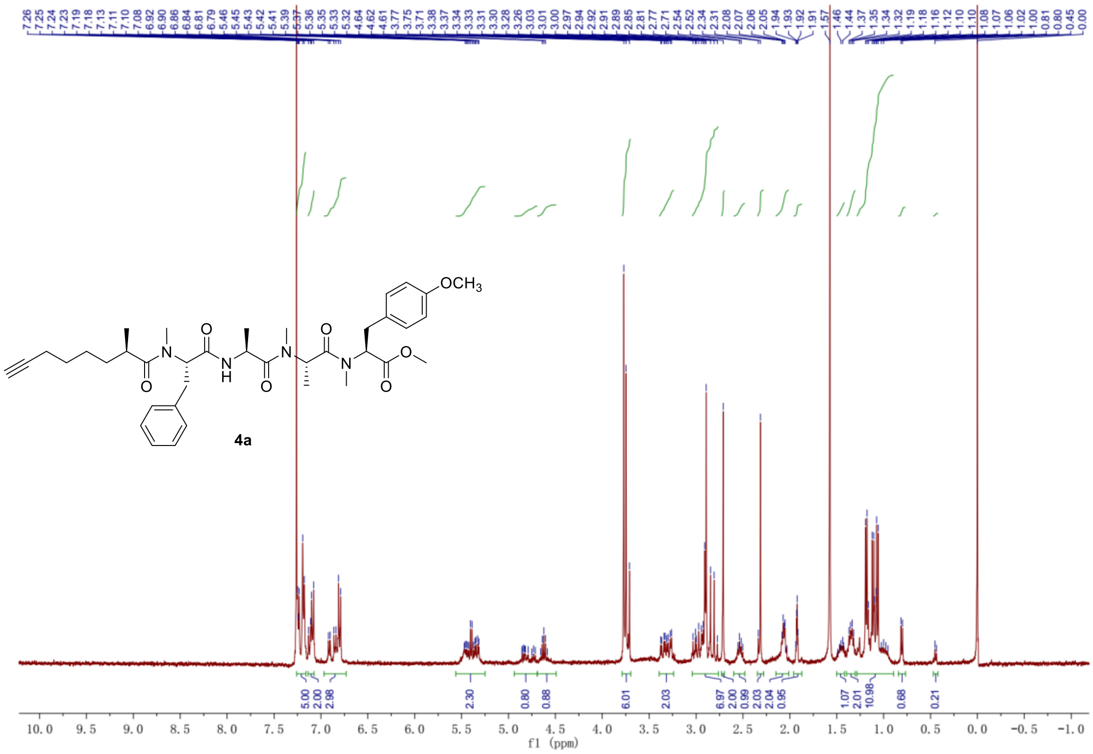

Figure S57. ^{1}H NMR (400 MHz, $CDCl_3$) spectrum of compound **4a**.

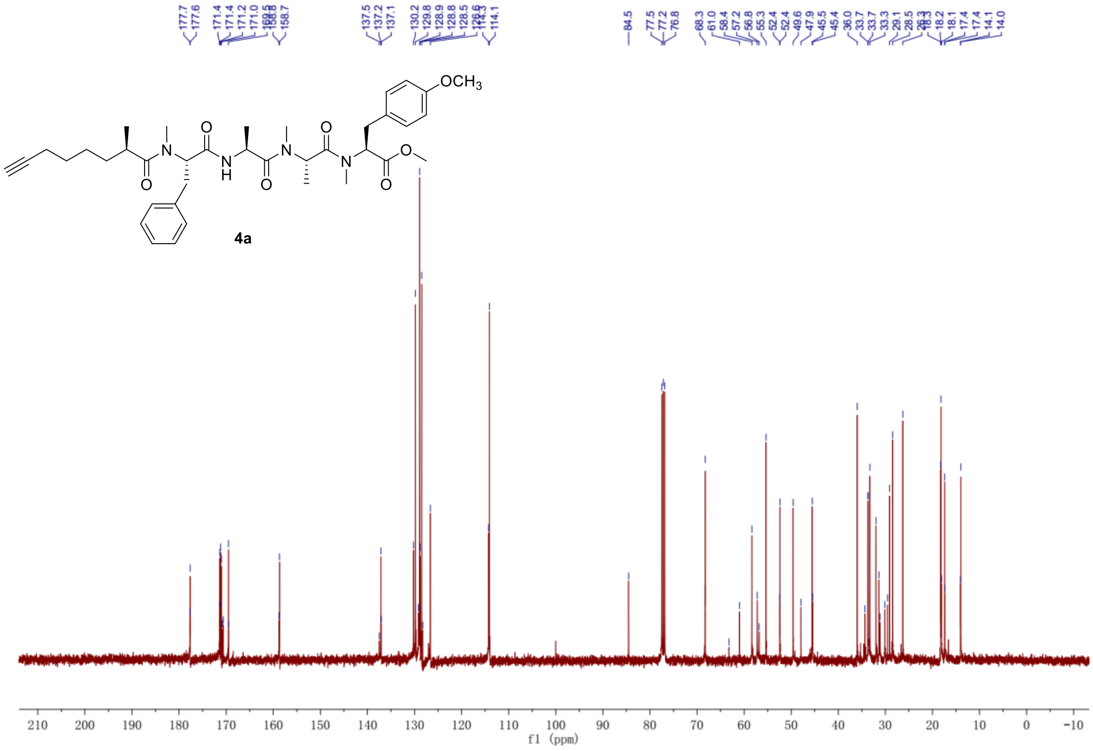

Figure S58. ^{13}C NMR (100 MHz, $CDCl_3$) spectrum of compound **4a**.

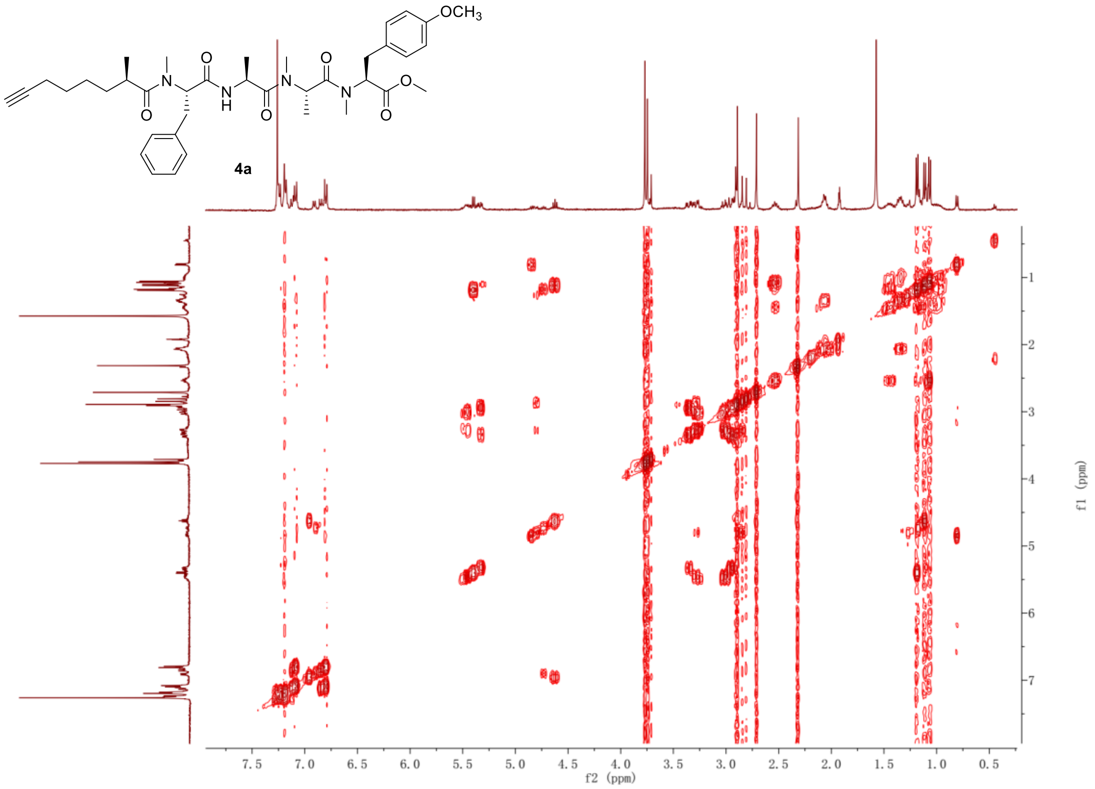

Figure S59. COSY (^{1}H, 400 MHz, $CDCl_3$) spectrum of compound **4a**.

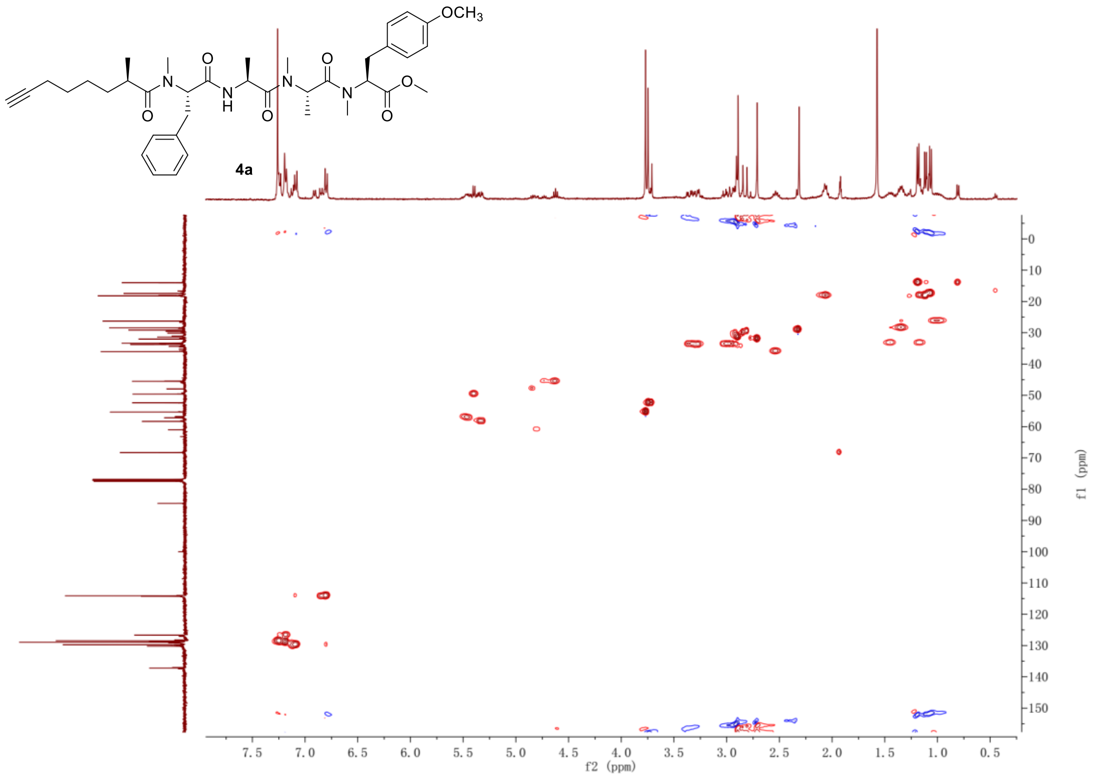

Figure S60. HSQC (^{1}H, 400 MHz, ^{13}C, 100 MHz, $CDCl_3$) spectrum of compound **4a**.

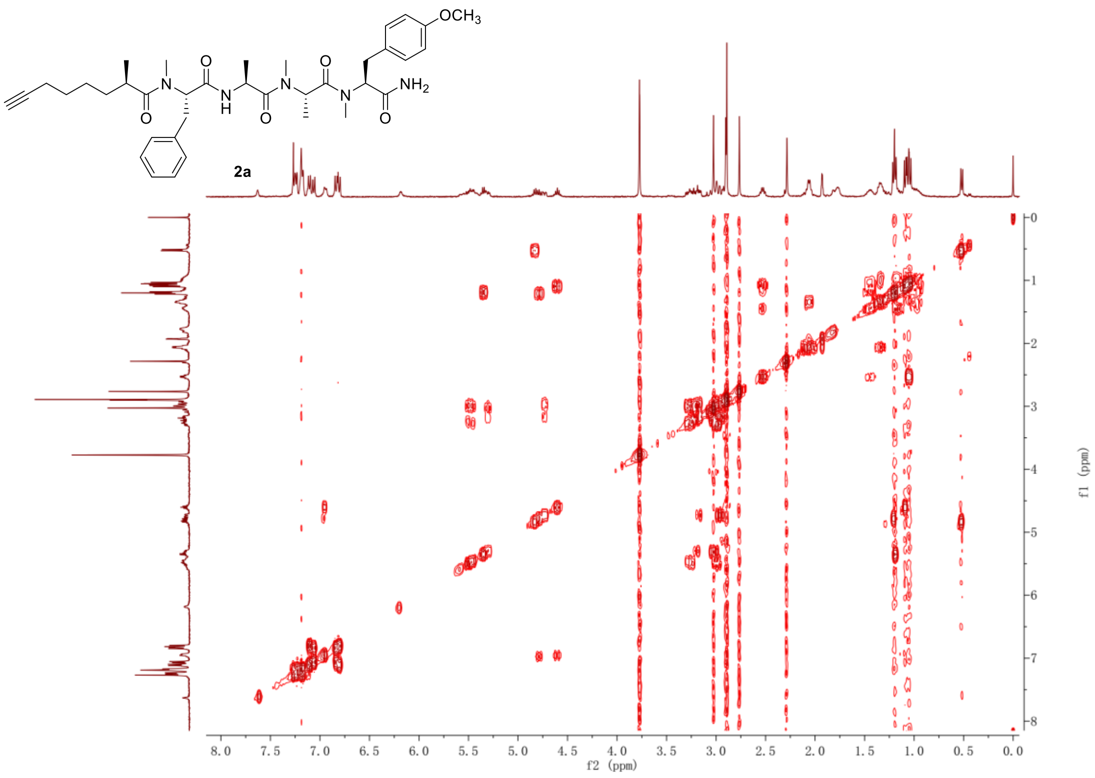

Figure S61. COSY (^{1}H, 400 MHz, $CDCl_3$) spectrum of synthetic **dragomabin (2a)**.

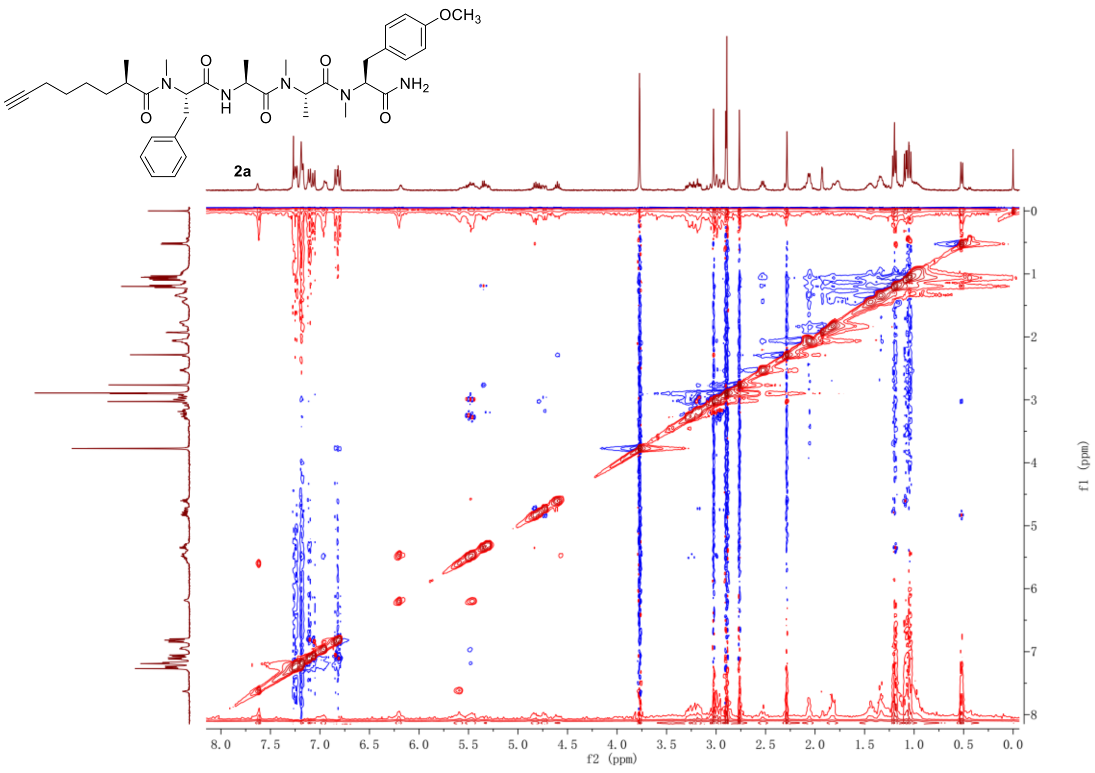

Figure S62. NOESY (^{1}H, 400 MHz, $CDCl_3$) spectrum of synthetic **dragomabin (2a)**.

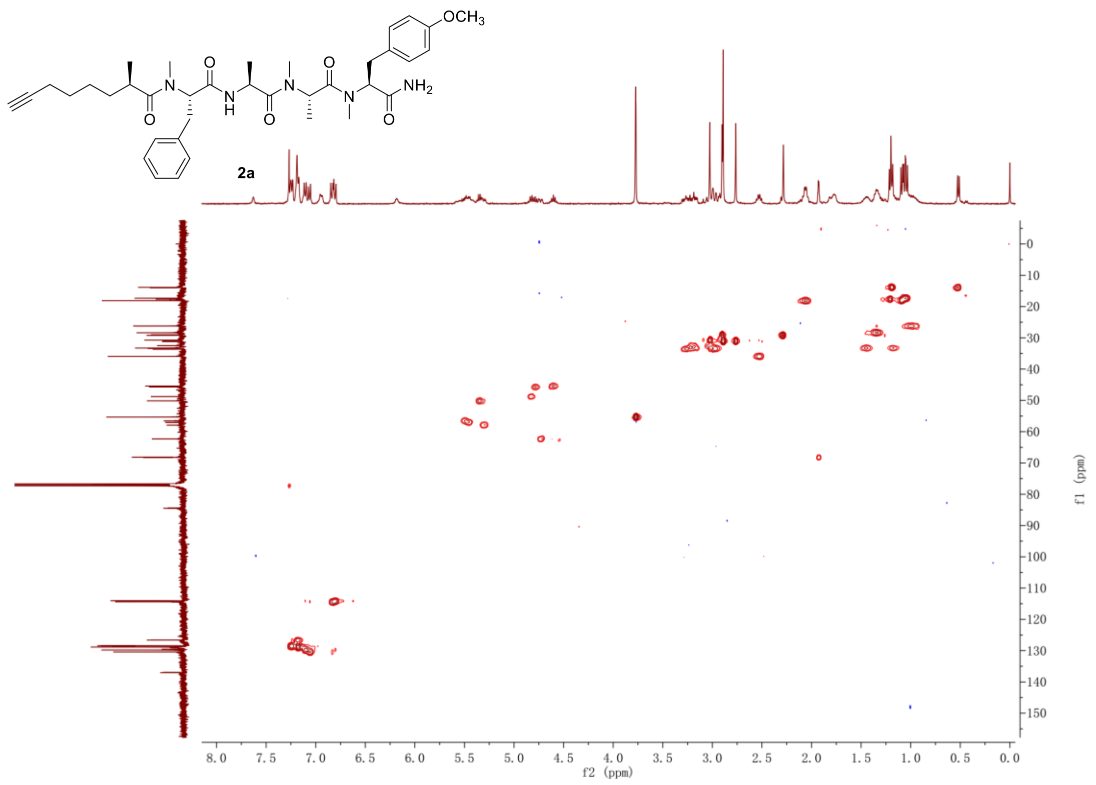

Figure S63. HSQC (^{1}H, 400 MHz, ^{13}C, 100 MHz, $CDCl_3$) spectrum of synthetic **dragomabin (2a)**.

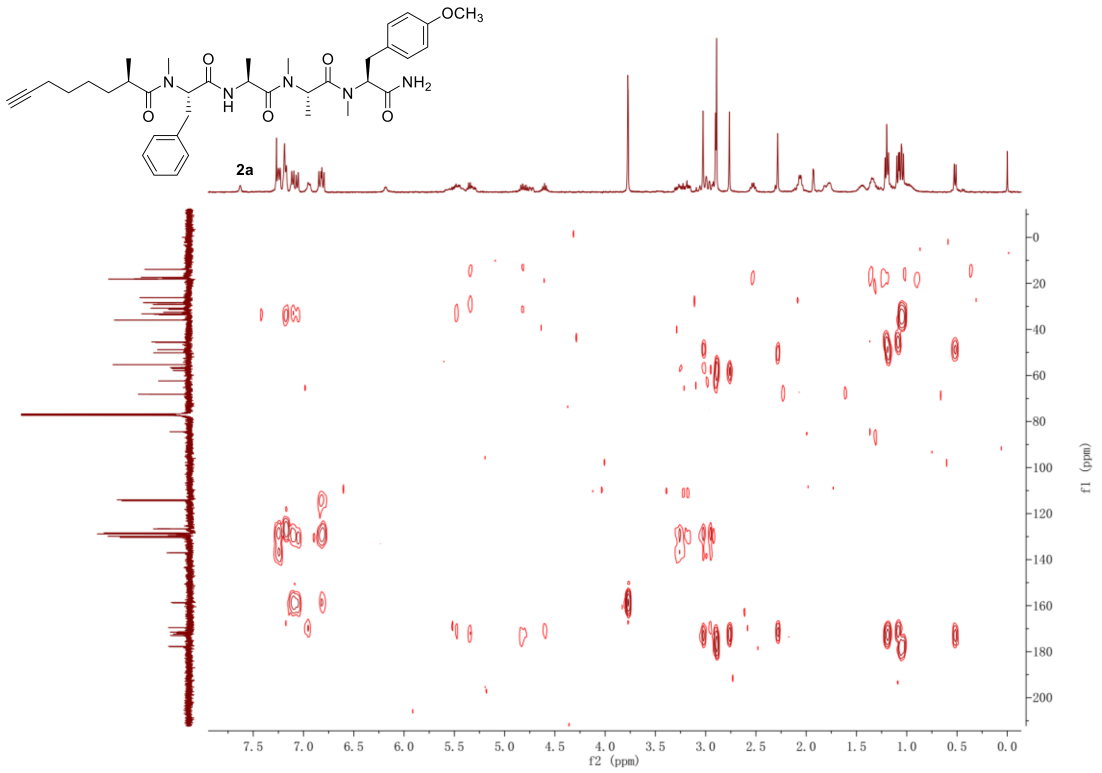

Figure S64. HMBC (^{1}H, 400 MHz, ^{13}C, 100 MHz, $CDCl_3$) spectrum of synthetic **dragomabin (2a)**.
